# Relationship between dominance hierarchy steepness and rank-relatedness of benefits in primates

**DOI:** 10.1093/beheco/arae066

**Published:** 2024-08-13

**Authors:** Pengzhen Huang, Malgorzata E Arlet, Krishna N Balasubramaniam, Brianne A Beisner, Eliza Bliss-Moreau, Lauren J N Brent, Julie Duboscq, Iván García-Nisa, Stefano S K Kaburu, Rachel Kendal, Martina Konečná, Pascal R Marty, Brenda McCowan, Jérôme Micheletta, Julia Ostner, Oliver Schülke, Gabriele Schino, Bonaventura Majolo

**Affiliations:** School of Psychology, University of Lincoln, Brayford Wharf East, Lincoln, LN5 7AY, United Kingdom; School of Ecology, Hainan University, Hainan, China; Institute of Human Biology and Evolution, Faculty of Biology, Adam Mickiewicz University, 61614 Poznań, Poland; School of Life Sciences, Faculty of Science and Engineering, Anglia Ruskin University, Cambridge CB1 1PT, United Kingdom; Animal Resources Division, Emory National Primate Research Center, Emory University, Atlanta, GA 30329, United States; Department of Psychology, University of California, Davis, CA 95616, United States; California National Primate Research Center, University of California, Davis, CA 95616, United States; Centre for Research in Animal Behaviour, University of Exeter, Exeter EX4 4QG, United Kingdom; Unité Eco‑Anthropologie (EA), UMR 7206, Muséum National d’Histoire Naturelle, CNRS, Université Paris Cité, Musée de l’Homme 17 Place du Trocadéro, 75016 Paris, France; Department of Behavioral Ecology, University of Göttingen, Göttingen, Germany; Department of Anthropology, Durham University, Durham DH1 3LE, United Kingdom; School of Animal, Rural & Environmental Sciences, Nottingham Trent University, Southwell NG25 0QF, United Kingdom; Department of Anthropology, Durham University, Durham DH1 3LE, United Kingdom; Department of Zoology, Faculty of Science, University of South Bohemia, České Budějovice, Czech Republic; Wildlife Park Goldau, Parkstrasse 38, CH-6410 Goldau, Switzerland; California National Primate Research Center, University of California, Davis, CA 95616, United States; Department of Population Health and Reproduction, School of Veterinary Medicine (SVM), University of California at Davis, Davis, CA 95616, United States; Centre for Comparative and Evolutionary Psychology, Department of Psychology, University of Portsmouth, Portsmouth PO1 2UP, United Kingdom; Department of Behavioral Ecology, University of Göttingen, Göttingen, Germany; Social Evolution in Primates Group, German Primate Center, Leibniz Institute for Primate Research, Göttingen, Germany; Leibniz ScienceCampus Primate Cognition, Göttingen, Germany; Department of Behavioral Ecology, University of Göttingen, Göttingen, Germany; Social Evolution in Primates Group, German Primate Center, Leibniz Institute for Primate Research, Göttingen, Germany; Leibniz ScienceCampus Primate Cognition, Göttingen, Germany; Istituto di Scienze e Tecnologie della Cognizione, Consiglio Nazionale delle Ricerche, Rome, Italy; School of Psychology, University of Lincoln, Brayford Wharf East, Lincoln, LN5 7AY, United Kingdom

**Keywords:** distribution, dominance rank, fitness-related benefits, hierarchy steepness, resource acquisition

## Abstract

In animal social groups, the extent to which individuals consistently win agonistic interactions and their ability to monopolize resources represent 2 core aspects of their competitive regime. However, whether these two aspects are closely correlated within groups has rarely been studied. Here, we tested the hypothesis that hierarchy steepness, which is generally used to represent power differentials between group members, predicts the variation in the distribution of fitness-related benefits (i.e. fecundity, infant survival, mating success, and feeding success) in relation to individual dominance ranks. We tested this hypothesis in primate groups using comparative phylogenetic meta-analytical techniques. Specifically, we reviewed published and unpublished studies to extract data on individual dominance ranks, their access to fitness-related benefits, and hierarchy steepness. We collected and included in our analysis a total of 153 data points, representing 27 species (including 2 chimpanzee sub-species). From these, we used 4 common methods to measure individual dominance ranks and hierarchy steepness, i.e. *D*_*ij*_-based normalized David’s scores, randomized Elo-ratings, and David’s scores and Elo-ratings estimated in Bayesian frameworks. We found that hierarchy steepness had no effect on the strength of the relationship between dominance rank and access to fitness-related benefits. Our results suggest that hierarchy steepness does not reflect between-group variation in the extent to which individual dominance affects the acquisition of fitness-related benefits in primates. Although the ability to win agonistic encounters is essential, we speculate that other behavioral strategies adopted by individuals may play crucial roles in resource acquisition in animal competitive regimes.

## Teaser

In social animals, group members compete to attain dominant positions. Dominant individuals are expected to have better access to key resources, like food or mating. In our study, we show that the strength of the dominance hierarchy does not affect the distribution of key resources in primates. The distribution of social bonds and agonistic support between group members may weaken the effect of dominance hierarchy on how key resources are accessed by dominants and subordinates.

## Introduction

In gregarious animals, individuals may compete for access to resources that affect their fitness, such as food, water, or mating opportunities, depending on the value (i.e. how much a given unit of the resource affects individual fitness) and availability (i.e. how much of the resource is accessible in a given time/space) of these resources (Harcourt 1987; Silk 1987). Resource competition may lead to agonistic interactions of varying frequency and intensity between individuals (Whitten 1983; Bercovitch and Strum 1993). Over repeated interactions among the same individuals, dominance relationships may form that reflect differences in competitive abilities or/and in resource holding potential between group members (Dunbar and Dunbar 1977; Barton and Whiten 1993). Generally, dominant, higher-ranking individuals win all or the majority of dyadic agonistic interactions with their opponents and thus might be prone to monopolizing or have priority of access to resources over subordinate, lower-ranking individuals during contest competition (Bernstein 1976; Strum 1982). For example, when a group of spotted hyenas (*Crocuta crocuta*) converge on a carcass to feed, social rank of the individuals generally determines the priority of access to food (Watts and Holekamp 2007). Occupying a high dominance status in the group is also advantageous when individual woodland caribous (*Rangifer tarandus*) compete for limited food resources (Barrette and Vandal 1986). Similarly, higher-ranking female primates have greater infant survival to one year of age, and higher-ranking male primates mate more often than lower-ranking individuals (Majolo et al. 2012; also see review in Snyder-Mackler et al. 2020). Such monopolization of resources by higher-ranking individuals leads to an uneven distribution of resources among group members (Silk 2007a; Stockley and Bro-Jørgensen 2011). However, there is still limited evidence for whether, or to what extent, inter- and intra-specific variation in dominance hierarchies is linked to the animals’ ability to monopolize resources.

In primates, the extent to which an individual’s social rank determines its access to resources or its reproductive success varies not only within a group over time and contexts but also across groups of the same species and across taxa (Fedigan 1983; Barton and Whiten 1993; Stockley and Bro-Jørgensen 2011). For example, in a wild group of chacma baboons (*Papio ursinus*), dominant males tended to obtain more food in patches characterized by higher than average feeding competition, as compared to patches with lower levels of feeding competition (King et al. 2008). In wild crested macaques (*Macaca nigra*), the yearly proportion of paternity by the alpha male varied across groups and years, ranging from 29% to 100% (Engelhardt et al. 2017). However, while the association between dominance rank and access to resources and fitness has been repeatedly investigated (e.g. Cowlishaw and Dunbar 1991; Majolo et al. 2012; Shivani et al. 2022), across-group or across-species comparative approaches that explore the variation of these effects are less common (but see Perlman et al. 2016).

Hierarchy steepness is one of the indices that can be used to analyze variation in the distribution of agonistic interactions in social species (Gammell et al. 2003; de Vries et al. 2006), and indeed, research has shown that it can vary substantially within and across species (Balasubramaniam et al. 2012a; Kaburu and Newton-Fisher 2015a). Hierarchy steepness captures the magnitude and consistency of the differences in wins and losses in dyadic agonistic encounters among group members (Gammell et al. 2003; de Vries et al. 2006). A steeper dominance hierarchy implies a greater certainty for dominants of winning agonistic contests and should therefore translate into a greater ability to monopolize contestable resources. Mathematically, hierarchy steepness is calculated as the absolute slope of the regression of the cardinal dominance ranks (i.e. the numerical values derived from the algorithms described below) of group members against their ordinal ranks (i.e. the order of the cardinal ranks) (de Vries et al. 2006). Several methods have been proposed to obtain cardinal ranks and derive the steepness of a dominance hierarchy (Sánchez-Tójar et al. 2018; Neumann and Fischer 2022). Due to these methodological advancements, the steepness of dominance hierarchies has been used to investigate how variation in the ability to win agonistic encounters translates in variation in the ability to access contested resources (Surbeck et al. 2011; Kaburu and Newton-Fisher 2015b). In particular, Perlman et al. (2016) presented comparative data on features of the dominance hierarchy and alpha male paternity in 9 primate species and concluded that hierarchy characteristics and paternity monopolization were not related. The small sample of species and the lack of any formal statistical comparative analysis make this conclusion in need of a more quantitative assessment.

The aim of this study was to test whether the variation in hierarchy steepness was related to the extent to which disparities in the distribution of fitness-related benefits are related to dominance rank. We measured 4 kinds of fitness-related benefits, i.e. fecundity, infant survival (to the first year), mating success, and feeding success (Silk 2007b; Majolo et al. 2012). Among them, individual fecundity and infant survival are direct components of individual reproductive success, while individual mating success (Alberts et al. 2006) and feeding success (Whitten 1983; Koenig 2002) are proxies of individual fitness outcomes (Whitten 1983; Arnqvist and Nilsson 2000; Whitehead and Rendell 2004). We employ meta-regression techniques in combination with phylogenetic comparative methods to quantitatively synthesize the results of available published and unpublished data and assess whether the relation between fitness-related benefits and dominance rank is modulated by the degree of hierarchy steepness. We hypothesized that the steeper the dominance hierarchy of a group is, the greater the disparities in fitness-related benefits toward dominants compared to subordinates. This is because, when resource characteristics allow monopolization, dominant individuals should be incentivized to monopolize contestable resources and to consistently win agonistic contests against subordinates, which in turn should translate into a steeper dominance hierarchy and more pronounced rank-related fitness differences between individuals. This hypothesis potentially applies to any species that lives in relatively cohesive social units. We tested it in primates, that are characterized by diverse dominance styles (de Waal 1989; Vehrencamp 1983) and are the subject of an extensive literature. The large available database, the multiple methodologies available on dominance hierarchies, and the combined use of modern meta-analytical techniques and comparative methods allowed us to run a comprehensive and robust test of the hypothesis that variations in characteristics of the dominance hierarchy are associated to variations in the ability of dominants to monopolize fitness relevant resources, gaining insights into the evolution of competitive regimes in social animals (e.g. Fedigan 1983; Cowlishaw and Dunbar 1991; Majolo et al. 2012).

## Methods

### Literature search

**Fig. 1.**
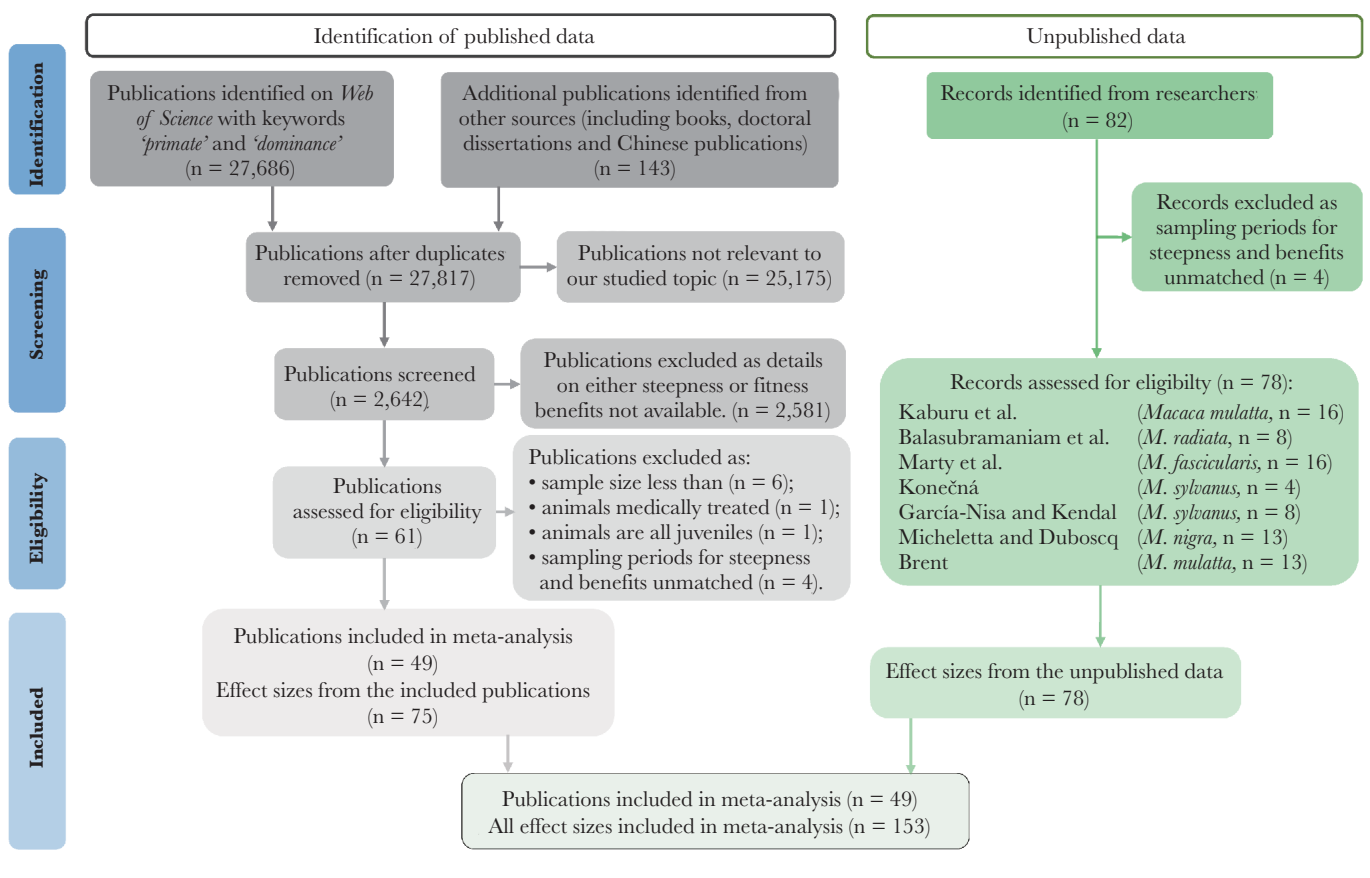
PRISMA flow diagram depicting the different steps of the literature review conducted for this study. Detailed information on the criteria used for including and excluding studies is presented in the Methods section.

For the systematic literature review, we followed the statement of preferred reporting items for systematic reviews and meta-analyses (PRISMA) (O’Dea et al. 2021). We searched all literature (articles) on the *Web of Science* using the keywords *dominance* and *primate* from January 1970 to February 2022. We also reviewed books, dissertations, and Chinese publications on group-living primates as supplementary resources. To be included in our study, the output (published paper or unpublished data) had to contain information about both hierarchy steepness and the distribution of individual fitness-related benefits across group members in relation to individual dominance ranks. These two pieces of information had to be from the same study group during the same study period (**Fig. 1**). Information on the dominance hierarchy included the matrix of aggressive and/or submissive interactions during contest competition **(see****Supplementary****raw data)**, cardinal ranks of sampled individuals, or the value of hierarchy steepness of the sampled group. We then correlated individual fitness-related benefits with dominance ranks (cardinal or ordinal). The specific and detailed definition of each fitness-related benefit is shown in **Table 1**.

**Table 1.** Summary and specific definitions of 4 fitness-related benefits investigated in this study.

Fitness-related benefit	Sex	Definition
Fecundity	Female	Mean number of births or the length of the inter-birth interval^a^^,^^b^
	Male	Number of infants sired^c^
Infant survival	Female	Number of infants surviving to the first year of life^d^
Mating success	Both	Number/frequency of copulations with ejaculation (including copulations where ejaculation could not be determined with certainty)^e^
Feeding success	Both	Number/frequency of food intake, frequency of energy intake (caloric intake per minute), time spent feeding^b^, or ponderal index (body length/body weight)^f^

^a^ Fairbanks and McGuire 1984;

^b^ Strum and Western 1982;

^c^ Majolo et al. 2012;

^d^ Fedigan 1983;

^e^ Berard et al. 1993;

^f^ Whitten 1983.

In cases where an output contained information on hierarchy steepness only, we searched other available outputs about fitness-related benefits that focused on the same study group during the same study period. Similarly, when an output tested the relationship between individual fitness-related benefits and dominance ranks only, we looked for outputs containing the information on the hierarchy steepness of the study group during the same study period. When the sampling period of a study for the hierarchy steepness did not match the period for the fitness-related benefits (e.g. Dolhinow et al. 1979), we excluded these studies from our dataset.

We excluded studies based on a sample size of less than 4 individuals and where the sampled animals contained juveniles (e.g. Lee and Oliver 1979) or were medically treated (i.e. Huber et al. 2015) because these procedures can bias differences in fitness measures across animals. To expand our dataset via the inclusion of unpublished data, we emailed researchers who are part of the MacaqueNet big-team science consortium (https://macaquenet.github.io/) and those who work on dominance rank, asking them if they were willing to share their unpublished raw data with us **(see****Supplementary Material 2**). Unpublished data had to meet the same selection criteria described above to be included in our study.

### Data extraction

From the outputs that met our selection criteria and the unpublished data provided by researchers, we extracted information on the study setting (i.e. wild, provisioned, or captive), study duration (length of the data collection, in months), species, sex of sampled animals (females, males or both: data could not be divided by sex of the animals), sample size (the number of animals tested), information for calculating hierarchy steepness (mainly the dominance matrix), the type of fitness-related benefits, and how they were measured. Among the 4 types of fitness-related benefits for which we had data (see above), we considered fecundity and infant survival as 2 direct measures of fitness, because they differently but reliably quantify reproductive success (Wilson 2000; Jones 2011). Conversely, we treated mating and feeding success as indirect fitness measures. This is because the mating frequency and the number of mating partners do not always translate into greater reproductive success (Fedigan 1983); this happens, for example, when a male mates with a female that has passed the peak of her estrous (Young et al. 2013). Moreover, having access to larger quantities of food can both improve and deteriorate health and survival (e.g. caloric food increases adipose mass), and it does not necessarily result in greater reproductive success (Janson 1988; Koenig 2002). Thus, we considered more conservative to treat mating and feeding success as 2 indirect measures of individual fitness.

All unpublished data provided by the researchers contained the raw data on the distribution of fitness-related benefits among group members. As a result, a total of 78 data points were collected from unpublished data. Regarding the selected published outputs, we tried to extract such raw data from the main text (e.g. Jones 1980; Hilpert and Jones 2005) or the supplementary materials (e.g. De la Fuente et al. 2019), whenever these data were available. When the raw data were presented as figures (mostly scatterplots with a trend line), we used the free online tool WebPlotDigitizer (https://automeris.io/WebPlotDigitizer) to extract the values of individual benefits from the graph (e.g. Reed et al. 1997). Following this procedure, among the 75 data points we collected from the published outputs, there were 59 data points from which we were able to extract raw data on the individual fitness-related benefits. If no raw data were provided, we directly recorded the type of statistical tests and statistics of the relationship between individual dominance ranks and fitness-related benefits reported in the outputs.

### Data processing and management

With the dominance matrices extracted from the selected outputs and unpublished data, we calculated individual cardinal and ordinal ranks, the steepness of the dominance hierarchy for each sampled group in the statistical environment R 4.1.2 (R Core Team 2021; Leiva and de Vries 2022). *D*_*ij*_-based normalized David’s score is a frequently used cardinal measure of individual dominance ranks: the hierarchy steepness is represented by the absolute slope of the regression of the cardinal dominance ranks of group members against their ordinal ranks (de Vries et al. 2006). We referred to this measure as *NDS*_*Dij*_ and obtained individual *NDS*_*Dij*_ (cardinal ranks) and *NDS*_*Dij*_-based steepness using *getNormDS* and *steeptest* functions of the “steepness” package, respectively (Leiva and de Vries 2022). As the reliability of *NDS*_*Dij*_-based steepness is highly dependent on data density and the amount of the dyadic unknown relationships in the dominance hierarchy (Schino and Aureli 2008; Klass and Cords 2011), we considered 3 additional measures for individual cardinal ranks and group hierarchy steepness in our analysis: *Elo*_rpt_, *Elo*_Bayes,_ and *DS*_Bayes_. Based on the original Elo-rating, which is a sequential method that allows the visualization of hierarchy dynamics (Elo 1978; Neumann et al. 2011), Sánchez-Tójar et al. (2018) proposed an amendatory measure to compute steepness. This measure translates the interaction matrix into a large number of interaction sequences and then obtains individual randomized Elo-ratings (cardinal ranks) from these simulated interaction datasets. The repeatability of the Elo-ratings across the randomizations serves as steepness through estimating the uncertainty of the inferred hierarchy (Nakagawa and Schielzeth 2010). We indicated this measure as *Elo*_rpt_ and used it as the second measure for individual cardinal ranks and group steepness. We computed individual *Elo*_rpt_ and *Elo*_rpt_-based steepness with *elo_scores* and *estimate_uncertainty_by_repeatability* functions of the “aniDom” package (Sánchez-Tójar and Farine 2021), respectively.

Neumann and Fischer (2022) recently developed a novel approach to estimate steepness based on Elo-rating scores and David’s scores (DS) in a Bayesian framework. They highlighted the differences with other available methods regarding the response to data density and demonstrated that the steepness estimation with Elo-rating in a Bayesian framework was unbiased and more reliable than *DS*-based steepness (Neumann and Fischer 2022). These two steepness algorithms additionally provide credible intervals, which help to infer the uncertainty of steepness (Neumann and Fischer 2022). We thus applied Bayesian Elo-ratings as the third measure and referred to it as *Elo*_Bayes_. We calculated individual *Elo*_Bayes_ (cardinal ranks) and *Elo*_Bayes_-based steepness using *scores* and *elo_steepness_from_matrix* functions of the “EloSteepness” package in R 4.1.2 (Neumann 2022). The Bayesian David’s score was our fourth measure and indicated as *DS*_Bayes_. We calculated individual *DS*_Bayes_ (cardinal ranks) and *DS*_Bayes_-based steepness using *scores* and *davids_steepness* functions of the “EloSteepness” package (Neumann 2022).

Based on the individual cardinal ranks evaluated by the 4 measures, i.e. *NDS*_*Dij*_, *Elo*_rpt_, *Elo*_Bayes_, and *DS*_Bayes_, we calculated the corresponding ordinal ranking and named them as *NDS*_*Dij*_-based, *Elo*_rpt_-based, *Elo*_Bayes_-based, and *DS*_Bayes_-based ordinal ranks, respectively. As calculating steepness may have little meaning in the absence of a linear hierarchy, we also calculated the modified Landau’s linearity index and its significance for each sampled group (using the *h.index* function of the “EloRating” package; de Vries 1995; de Vries et al. 2006; Neumann and Kulik 2020) so as to be able to rerun the analyses including only data on social groups that had a significantly linear dominance hierarchy.

In a few cases (Supplementary Appendix 1), there was more than one measure used to evaluate individual feeding success in a specific social group during a specific study period. If so, we calculated the mean value of the correlation coefficients calculated from the available measures to represent the overall feeding success in relation to individual social ranks. Similarly, when there was more than one matrix of aggressive and/or submissive interactions for a specific study period, during which the fitness-related benefits of a social group were measured, we used the mean value, calculated from the available matrices, to represent the overall degree of hierarchy steepness (e.g. Borries et al. 1991).

With the raw data on the fitness-related benefits, for each output we computed Pearson correlation coefficients of the relationship between individual fitness benefits (4 measures) and the 4 measures of cardinal ranks. Similarly, we calculated Pearson correlation coefficients for the relationship between individual fitness benefits and ordinal ranks. In the outputs where raw data were not available, the benefit distribution among group members in relation to their dominance ranks was represented by correlation coefficients (*n* = 12, mostly Spearman), chi-square statistics (*n* = 3), or mean ± standard deviation (M ± SD) values (*n* = 1). We transformed chi-square statistics and M ± SD values into correlation coefficients using the web-based effect size calculator (https://www.campbellcollaboration.org/escalc/). Finally, all correlation coefficients were transformed to Fisher’s *Z*_*r*_ values, which is an effect size standardizing the effect of an independent variable on a dependent variable (Nakagawa and Cuthill 2007; Borenstein et al. 2009). For each data point in our dataset, the effect size represented the relationship between individual cardinal/ordinal ranks and the individual values for the fitness-related benefits for that individual. A positive effect size indicates that dominants obtain more benefits than subordinates, whereas a negative effect size indicates the opposite pattern (subordinates gain more benefits than dominants). The sampling variance related to each effect size and output was calculated based on the sample size of each study (Borenstein et al. 2009).

**Fig. 2.**
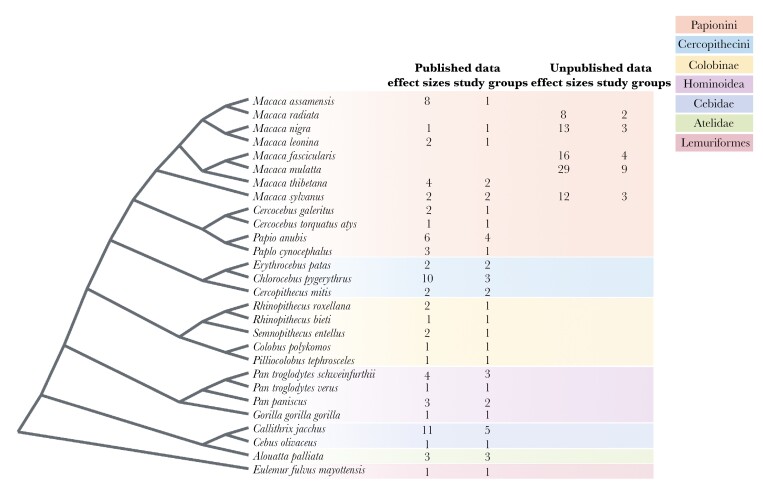
Phylogenetic tree for the species included in our study. The numbers of effect sizes and study groups, from published and unpublished data, are indicated next to the scientific name of each species.

**Fig. 3.**
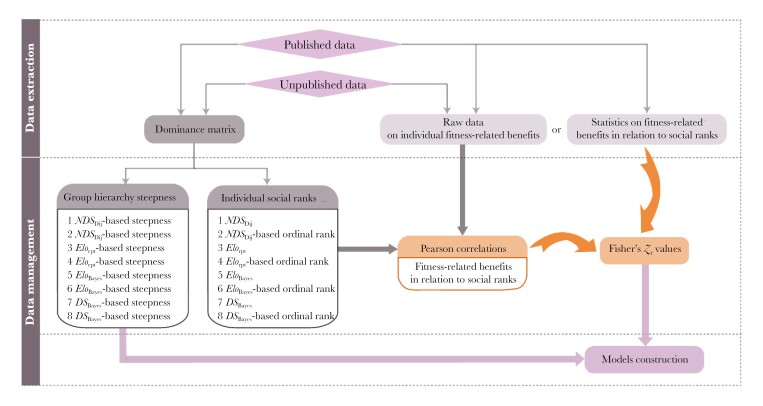
Illustrative framework of the data processing steps of this study.

Our final dataset included 153 data points (see Supplementary Appendices 1–2 in the **Supplementary Material 1**): 75 data points from 49 published outputs and 78 data points from unpublished data (**Fig. 1**) on 27 species (including 2 chimpanzee sub-species) in 64 groups (**Fig. 2**). Of the 153 data points included in our analyses, 41 were on direct fitness-related benefits (i.e. fecundity and infant survival) and 112 on indirect benefits (i.e. mating success and feeding success). An illustrative framework for the methodology of this study is shown in **Fig. 3**.

### Model construction

All data analyses were performed in the statistical environment R 4.1.2 (R Core Team 2021). We ran a series of mixed-effects meta-regression models to evaluate whether the variation in the distribution of fitness-related benefits among group members, relative to their dominance ranks, was related to the group hierarchy steepness. For each steepness measure, we examined its influence on the effect sizes calculated by the benefits individuals gained against their corresponding cardinal ranks and ordinal ranks. For example, when the *NDS*_*Dij*_-based steepness was the predictor, the response variable in the first model was the effect size represented by the relationship between the benefits that each individual obtained and their *NDS*_*Dij*_. In the second model, the effect size was the relationship between individual benefits and their *NDS*_*Dij*_-based ordinal ranks. Thus, we constructed a total of 8 full meta-regression models (2 models for each steepness measure).

In each model, we entered one of the 4 steepness measures as the predictor variable. Moreover, we included 7 moderators as control variables in our full models: (1) the category of the fitness-related benefit (categorical: direct or indirect, as stated above); (2) study duration (continuous, in months); (3) study setting (categorical: wild, provisioned, or captive); (4) sex category of sampled animals (categorical: female, male or both); (5) dispersal pattern of the study species (categorical: female philopatry, male philopatry or no sex-biased philopatry); (6) social organization of the study species (categorical: one-male group/OMG, multimale-multifemale group/MMG or multilevel society/MLS); (7) data origin (categorical: published or unpublished). We included these control variables because they are known to affect competitive regimes in primates and for their potential effects on the response variable (Fedigan 1983; Sterck et al. 1997; Koenig and Borries 2006; Majolo et al. 2012). The data on the dispersal pattern and the social organization of each species were taken from Olivier et al. (2024), Mitani et al. (2012), and Shultz et al. (2011). The corresponding control models contained only the 7 control variables, plus our 3 random factors (study group, species, and phylogeny; see below), without the predictor variable.

To detect whether the relationship between steepness and the response variable was modulated by one of the control variables, we also included an interaction effect between steepness and the tested control variable in separate models. The corresponding control models contained the same variables but excluded the 2 main effects and their interaction.

Before running these models, we standardized continuous predictors with the *scale* function and centered categorical variables (Schielzeth 2010; Mundry 2014) with the *dummy_cols* function of the “fastDummies” package (Kaplan 2020). Based on Schielzeth (2010), centering the variables before fitting regression models largely minimizes the collinearity between them, and makes interpretation of the results easier, especially when testing an interaction effect.

For all meta-regression models, we entered study group ID, nested within species ID, as 2 random factors, to control for multiple data from the same group and the same species. We did not include study ID as a random factor in the models because several studies only provided one data point. Finally, we added phylogeny as a random factor in all models. We downloaded the consensus phylogenetic tree of the species included in our dataset from the website 10kTrees (http://10ktrees.nunn-lab.org/index.html) (Arnold et al. 2010). The consensus tree represents the collective agreement of all 10,000 trees in the tree block, and the nodes on the tree are delineated with high clade credibility values (Arnold et al. 2010). The tree was then made ultrametric and branch lengths were estimated using Grafen’s method with the *compute.brlen* function of the “ape” package (Grafen 1989; Paradis and Schliep 2019). We obtained the phylogenetic variance-covariance between species via the *vcv* function, which we incorporated into our models.

In all models, each observation was weighted by the inverse of the variance of the effect size, so that those observations based on larger sample sizes were weighted more than those based on smaller sample sizes (Gurevitch and Hedges 1999). We undertook model construction processes using the *rma.mv* function implemented in the “metafor” package (Viechtbauer 2010). We used a log-likelihood ratio test to assess the fit of each full model in relation to the corresponding control model with the *anova.rma* function of the “metafor” package (Viechtbauer 2010). Following the procedure used by Cinar and colleagues (2022), we quantified the phylogenetic signal (Pagel’s λ) in the overall variance components for each full model. To detect the effect of the phylogenetic signal on a phylogenetic model, we then performed a likelihood ratio test comparing the model with the respective non-phylogenetic model where the phylogenetic signal was zero (Münkemüller et al. 2012; Kamilar and Cooper 2013).

For each full meta-regression model, an omnibus moderator test was carried out and the Q_M_ statistic with a *P* value was used to detect the significance of the moderators, excluding the intercept. The omnibus test estimates whether there is a significant contribution of a moderator to the overall heterogeneity (Borenstein et al. 2009; Viechtbauer 2010). We also performed Q_E_ tests to assess unexplained residual heterogeneity for each full model. A significant Q_E_ test indicates there is a high level of between-study variance and other moderators, not included in the model, affect the remaining residual heterogeneity (Higgins and Thompson 2002; Viechtbauer 2010).

For the published data in our dataset, we assessed the occurrence of 2 types of publication biases, i.e. small-study effect and time-lag effect. Small-study effects refer to the pattern that effect sizes from smaller studies tend to be larger (Sterne et al. 2000, 2011). Time-lag effects occur when the effect sizes of published studies change over time (Poulin 2000; Jennions and Møller 2002) because, for example, studies that significantly support a hypothesis are published earlier than those that do not support the hypothesis (Koricheva et al. 2013). To examine temporal trends in our effect sizes that could indicate the above 2 effects, we constructed meta-regression models for each of our effect sizes with either the square root of the inverse of the sample size or the standardized publication year as the only fixed factors, and the same 3 random factors as full models (Nakagawa and Santos 2012; Nakagawa et al. 2022).

## Results

**Fig. 4.**
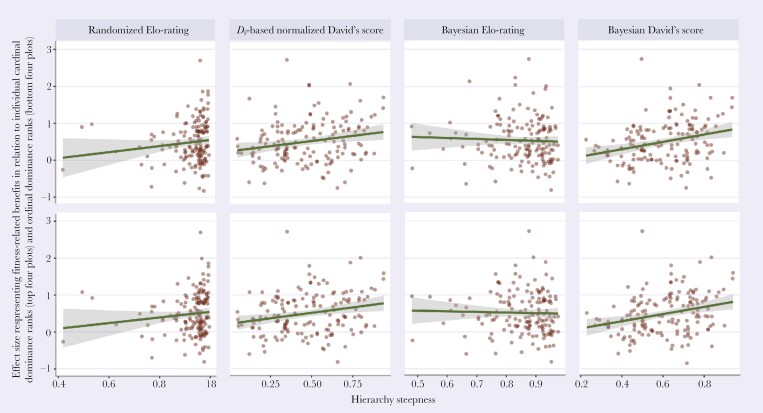
The data included in our analysis showing the relationship between hierarchy steepness and the fitness-related benefit distribution in relation to individual cardinal and ordinal social ranks (*n* = 153). The measures for hierarchy steepness and individual social ranks are indicated above the plots. Each data point is semi-transparent. The transparency value of the data points is accumulated when they are overlapped, making them appear darker. The line is fitted on the raw data with a linear model, and the shaded areas represent 95% confidence intervals.

Contrary to our hypothesis, we found no significant relationship between variation in the distribution of fitness-related benefits among group members in relation to dominance rank and the group hierarchy steepness (**Fig. 4**). None of the 8 full meta-regression models was significantly better than the corresponding control model (*χ*^2^ < 2.99, *P* > 0.08) (**Table 2**), suggesting that hierarchy steepness did not add any explanatory power to the models only including the control variables and random factors (Supplementary **Tables S1****–****S8**). The models showed extremely low phylogenetic signals (all λ < 0.01, all *P* > 0.99) (Supplementary **Tables S1****–****S8**), indicating that the variation in the detected variables was mostly independent of the phylogeny. For all full models, there was substantial unexplained heterogeneity, even after accounting for the moderators, suggesting that there were other unmeasured moderators contributing to the observed heterogeneity in the effect sizes (Supplementary **Table S9**).

**Table 2.** Test statistics of the likelihood ratio tests comparing each full model to the respective control model for the complete dataset (*n* = 153). The degrees of freedom of each full model and the respective model were 16 and 15, respectively.

Dominance rank measure	Steepness measure	Likelihood ratio test	
		*χ* ^2^	*P*
*NDS*_*Dij*_	*NDS*_*Dij*_	0.17	0.68
*NDS*_*Dij*_-based ordinal rank		0.19	0.66
*Elo*_rpt_	*Elo*_rpt_	2.99	0.08
*Elo*_rpt_-based ordinal rank		2.24	0.13
*Elo*_Bayes_	*Elo*_Bayes_	1.19	0.28
*Elo*_Bayes_-based ordinal rank		1.11	0.29
*DS*_Bayes_	*DS*_Bayes_	0.10	0.76
*DS*_Bayes_-based ordinal rank		0.10	0.75

We found some evidence of small-study effects in the published data. Studies with larger sample sizes had smaller effect sizes no matter which algorithm (i.e. *NDS*_*Dij*_, *Elo*_rpt_, *Elo*_Bayes_, or *DS*_Bayes_) the effect sizes were calculated with (all *Z* > 2.02, all *P* < 0.04) (Supplementary **Table S10**). Nonetheless, no time-lag effects were detected (all *Z* < 0.67, all *P* > 0.50) (Supplementary **Table S10**), suggesting a low probability that a time-lag bias is affecting our results.

When testing whether the effect of steepness was modulated by our control variables, we included 3 kinds of interaction effects separately in the full meta-regression models, i.e. the interactions between steepness and, respectively, the dispersal pattern of the study species, the study setting and the sex category of the study group. None of the interaction effects was significant, indicating that the relationship between the hierarchy steepness and the distribution of fitness-related benefits, in relation to the individual social ranks, was not modulated by these variables (dispersal pattern of the study species: Supplementary **Tables S11****–****S19**; study setting: Supplementary **Tables S20****–****S28**; sex category of the study group: Supplementary **Tables S29****–****S37**).

In our dataset, there are some study groups for which the dominance hierarchy were not significantly linear. Thus, we re-ran the analyses on a restricted dataset, which only contained data on social groups that had a significantly linear dominance hierarchy. Confirming the results of the model on the whole dataset, we found no evidence, in the restricted dataset, that hierarchy steepness significantly affected the distribution of fitness-related benefits in relation to individual social ranks (Supplementary **Tables S38****–****S45**). These full meta-regression models did not significantly explain more variance than their respective control models (**Table 3**).

**Table 3.** Test statistics of the likelihood ratio tests comparing each full model to the respective control model for the restricted dataset, including only groups in which the dominance hierarchy was significantly linear (*n* = 99). The degrees of freedom of each full model and the respective model were 16 and 15, respectively.

Dominance rank measure	Steepness measure	Likelihood ratio test	
		*χ* ^2^	*P*
*NDS*_*Dij*_	*NDS*_*Dij*_	0.00	1.00
*NDS*_*Dij*_-based ordinal rank		0.00	1.00
*Elo*_rpt_	*Elo*_rpt_	1.18	0.28
*Elo*_rpt_-based ordinal rank		0.64	0.42
*Elo*_Bayes_	*Elo*_Bayes_	0.52	0.47
*Elo*_Bayes_-based ordinal rank		0.33	0.57
*DS*_Bayes_	*DS*_Bayes_	0.00	1.00
*DS*_Bayes_-based ordinal rank		0.00	1.00

Finally, we excluded all control variables and built a set of simpler meta-regression models in which only the predictor (i.e. steepness) was included for both the full dataset and the restricted dataset (including only data on social groups that had a significantly linear dominance hierarchy). Again, we did not detect any significant effect of hierarchy steepness on the distribution of fitness-related benefits in relation to individual social ranks (Supplementary **Tables S46****–****S47**).

## Discussion

We tested whether the effect of individual dominance rank on the acquisition of fitness-related benefits in group-living primates is related to hierarchy steepness. We used 4 different measures to estimate individual ranks and hierarchy steepness, i.e. *D*_*ij*_-based normalized David’s scores, randomized Elo-ratings, Bayesian Elo-ratings and Bayesian David’s scores (Balasubramaniam et al. 2013; Sánchez-Tójar et al. 2018; Neumann and Fischer 2022). In contrast to our hypothesis, our analyses did not detect a significant effect of the hierarchy steepness on the disparities in the distribution of fitness-related benefits among group members in relation to their dominance rank. The significant results of the tests for heterogeneity indicated a large variation in the outcomes across different studies. These results suggest that the extent to which an individual consistently wins agonistic interactions, measured by hierarchy steepness, does not capture or reflect the variations in the extent to which dominance affects the acquisition of fitness-related benefits in primate groups. The 2 aspects of how competitive regimes are usually described, the consistency of winning agonistic interactions and the differential distribution of resources, appear thus to be only weakly related. Our results thus confirm and expand to both sexes and to other fitness measures, the weak relationship between dominance characteristics and male reproductive skew suggested by a previous study (Perlman et al. 2016). Moreover, our findings are consistent with previous research that found the influence of dominance rank on female reproductive success across macaque species is independent of their dominance styles (as evaluated by hierarchy steepness measured by David’s scores; Balasubramaniam et al. 2012b; Shivani et al. 2022). A recent agent-based model also showed that hierarchy steepness is not associated with the relationship between individual David’s score and energy intake. Instead, hierarchy steepness was more likely to be affected by whether resources were distributed heterogeneously or homogeneously (Ekanayake-Weber et al. 2024).

We found little evidence of publication bias. The small study effect that we detected seems more likely to indicate that in larger groups dominance relationships were not as pronounced as those in smaller groups. It is also important to note that most data for this study come from the genus *Macaca*, especially for the unpublished data. The restricted number of species represented in our dataset, and the fact that most species come from a few genera, means that we need to be cautious about the generalizability of our findings across the primate order. The representation of species, and of higher-level taxa, in the primatological literature is a problem that affects virtually every comparative analysis and that goes beyond the scope of our study (e.g. Schino 2001; Majolo et al. 2012). However, we obtained similar results when we ran the analyses separately on the published data (where the genus *Macaca* was not overrepresented) (Supplementary **Tables S48****–****S55**) or the unpublished data (Supplementary **Tables S56****–****S63**). Thus, our results may not be strongly affected by the presence of many macaque species.

Some components of dominance hierarchies show a strong phylogenetic signal, such as the Directional Consistency Index in nonhuman primates (Di Fiore and Rendall 1994) and the D_*ij*_ and P_*ij*_ indices of hierarchy steepness in the genus *Macaca* (de Vries et al. 2006; Balasubramaniam et al. 2012a). Conversely, we found a weak effect of phylogeny on the relationship between hierarchy steepness and the rank-relatedness of fitness benefits. One possibility is that phylogeny has a limited effect on how individual strategies (e.g. on when, how, and with whom to cooperate or compete; see below) integrate and generate group-level measures of dominance and skewness in fitness benefits (Revell et al. 2008). However, our analyses could not test for the presence of non-linear effects of phylogeny, such as Lévy process (Landis et al. 2013) or Ornstein-Uhlenbeck process (Butler and King 2004).

A possible explanation for our results is that current measures of hierarchy steepness do not in fact effectively describe power differentials between group members. For example, the normalized David’s score is known to be affected by sampling effort and sample size, yielding decreasing steepness values with lower data densities (Klass and Cords 2011; Balasubramaniam et al. 2012b) and larger group sizes (Richter et al. 2009). More recent measures, such as randomized Elo-rating (Sánchez-Tójar et al. 2018), however, control for the effect of sampling effort on the estimation of steepness; Bayesian Elo-rating is suggested to be the least biased and most robust to variation in data density among the available steepness measures (Neumann and Fischer 2022). Overall, the consistency of our results using 4 different steepness measures makes it unlikely that methodological inadequateness is at the basis of our findings. Furthermore, recent research has shown that primates can perceive small-scale variations in their cardinal ranks (as measured using normalized David’s score or randomized Elo-rating) and use this knowledge for their social decision making (Schino and Lasio 2019). Again, these observations suggest current measures of hierarchy steepness are not inadequate and do reflect significant aspects of social structure and of how animals themselves perceive it.

Given that methodological weakness does not seem to explain our results, it remains to be understood why variation in hierarchy steepness across primate groups is weakly related to variation in the ability of dominant animals to monopolize resources and gain fitness benefits. Recently, 2 studies ran individual-based evolutionary simulations of dominance behaviors and reproductive skew and found the evolution of dominance relationships and fighting behavior not to be strictly coupled to differential resource access. Ekanayake-Weber et al. (2024) found that clumped resources led to increased energy intake by dominants compared to subordinates, but did not necessarily lead to an increased steepness of the dominance hierarchy. The presence of compensating mechanisms (e.g. behavioral counter-strategies by subordinates, such as the use of alternative, lower-energy feeding sites to avoid direct conflicts by dominants) (Heesen et al. 2014) may explain the decoupling between the rank-related skew in resource access and the underlying degree of competition (Ekanayake-Weber et al. 2024). A modeling study by Leimar and Bshary (2022) also found that, although dominance relationships may change over time, dominants cannot always effectively interfere with subordinates’ resource access. The relationship between reproductive skew and dominance differentials depends both on the effectiveness of interference and on its cost for the dominants (Leimar and Bshary 2022). When the effectiveness is too low or the cost is too high, the interference trait does not evolve, meaning that dominants will not interfere with the subordinates’ access to resources even in the presence of clear dominance relationships. In addition, the models showed that the discrepancies between skew in resource access and dominance differentials are expected to be smaller among males than among females, because males generally suffer more damage and injuries in contest competition (Feder et al. 2019; Leimar and Bshary 2022). Contrary to what was suggested by these theoretical models, we did not find sex difference in the relationship between hierarchy steepness and the distribution of fitness-related benefits in relation to individual social rank.

We suggest that, besides wins and losses in agonistic interactions, the patterning and distribution of cooperative or affiliative interactions for both sexes may also influence the disparities in the distribution of fitness-related benefits. Through cooperative interactions, individuals exchange resources or services, or provide support in spite of suffering temporary costs (Axelrod and Hamilton 1981; Nowak 2006). Kin selection, mutualism, or reciprocity (Clutton-Brock 2009) can influence the acquisition of fitness-related benefits independently of dominance rank. For instance, genetic relationship between dominants and subordinates may contribute direct or indirect fitness to both (Eberhard 1975). In red howler monkeys (*Alouatta seniculus*), the alpha male father almost all offspring; however, the subordinate males, who are closely related to the alpha male, benefit indirectly and increase their inclusive fitness by forming long-term coalitions with the alpha (Pope 1990). In cooperatively breeding meerkats (*Suricata suricatta*), nonreproductive helpers may provide food items to help rear pups regardless of the helper’s dominance status (Clutton-Brock et al. 2001).

Apart from individual social rank, the patterning and distribution of affiliative interactions among group members, and emergent phenomena such as social integration and social bond strength, might, therefore, also influence the distribution of fitness benefits within groups (Ostner and Schülke 2018; Snyder-Mackler et al. 2020). For example, in yellow-bellied marmots (*Marmota flaviventris*), the female’s social ability to control their direct and indirect social relationships plays a more prominent role in reproductive skew than the traditional measure of dominance (i.e. linearity of dominance hierarchy) (Maldonado-Chaparro et al. 2024). In primates, allogrooming is a well-recognized cooperative behavior that can be traded for fitness-related resources, such as food and mating (Barrett et al. 1999; Schino 2001). Based on biological market principles, individuals experiencing high levels of within-group competition with a steep dominance hierarchy may benefit from trading grooming for access to resources (Henzi and Barrett 1999; Barrett et al. 2002). Indeed, there is some evidence that the steeper a dominance hierarchy is, the more grooming is directed up the hierarchy (Schino and Aureli 2008). Overall, however, studies linking hierarchy steepness to the relationship between grooming and fitness-related benefits have yielded different outcomes. Some studies have shown that the link between grooming and food tolerance is not associated with the degree of group hierarchy steepness (*M. sylvanus*, Roubová et al. 2015; *Pan paniscus*, Stevens et al. 2005). Conversely, in female *M. mulatta*, lower-ranking individuals are more likely to exchange grooming for drinking tolerance when the hierarchy is moderately steep (Balasubramaniam and Berman 2017). Moreover, male chimpanzees (*P. troglodytes schweinfurthii*) try to coerce mating when the hierarchy is steep, whereas they exchange grooming for mating when the hierarchy is shallow (Kaburu and Newton-Fisher 2015; see also a review in Dunayer and Berman 2016). Moreover, socioecological models predict that there is an association between coalition formation and contest competition for resources (Sterck et al. 1997; Pandit and van Schaik 2003). The formation of coalitions helps subordinate individuals, limiting the capacity of dominants to monopolize resources (Berghänel et al. 2010; Young et al. 2013); coalitions may also allow subordinates to achieve higher social ranks (Perry et al. 2008; Schülke et al. 2010; Neumann et al. 2022). Indeed, a growing literature shows that increased lifespan and reproductive success are often associated with strong cooperative exchanges independently of dominance rank (Parish 1996; Feh 1999; Brent et al. 2017; Alberts 2019).

Primates may also employ other behavioral strategies to acquire fitness-related benefits, for example, social tolerance and avoidance. In a comparative study between bonobos (*P. paniscus*) and chimpanzees, hierarchy steepness predicted the level of individual tolerance for food sharing (Jaeggi et al. 2010). Chimpanzees who formed a shallower dominance hierarchy shared food more frequently and actively than bonobos who lived in more despotic groups (Jaeggi et al. 2010). Avoidance may also be a powerful strategy if the monopolization of food resources is high (Koenig and Borries 2009). Whenever possible, subordinates may avoid feeding close to dominant individuals and choose to take a more peripheral position in the group to feed away from competitors (Janson 1985). As a result, food acquisition may not be rank-related, but subordinates face greater costs or risks by occupying a peripheral spatial position, such as greater foraging effort, distance to social partners, or predation risk (Monaghan and Metcalfe 1985; Schülke and Ostner 2012; Heesen et al. 2015). More investigations on these alternative behavioral strategies are needed to explicitly identify their role in resource distribution among group members in relation to their dominance hierarchy. It is important to determine to what extent these strategies benefit low-ranking individuals and offset the losses caused by their lack of competitiveness in resource competition. The available evidence suggests that the effects of these alternative strategies may not be strong enough to eliminate a general positive effect of dominance on resource acquisition and fitness (Majolo et al. 2012), but may weaken the relationship between hierarchy steepness and the disparities in resource acquisition and fitness (this study).

In conclusion, using 4 different algorithms to quantify dominance hierarchy, our study did not find evidence that the degree of inequality in the distribution of fitness-related benefits along the hierarchy is predicted by the steepness of the hierarchy in primate groups. We expect future studies to further assess the factors (e.g. other aspects of dominance hierarchies or social integration) that might influence individual access to fitness-related benefits in social groups.

## Supplementary Material

arae066_suppl_Supplementary_Data

arae066_suppl_Supplementary_Material_S1

arae066_suppl_Supplementary_Material_S2

## Data Availability

Analyses reported in this article can be reproduced using the data provided by Huang et al. (2024).
