# Supplementary material for: Relationship between dominance hierarchy steepness and rank-relatedness of benefits in primates
Source: Behav Ecol. 2024 Aug 13;35(5):arae066. doi: 10.1093/beheco/arae066 (PMC11347755; doi:10.1093/beheco/arae066)
Supplement: arae066_suppl_Supplementary_Material_S1 [file arae066_suppl_supplementary_material_s1.docx]

**Supplementary materials**

**List of figures and tables in supplementary materials**

**Figures S1-S2** The width of the 89% credible interval of *Elo*_Bayes_-based steepness and *DS*_Bayes_-based steepness, respectively.

**Tables S1-S8** For the complete dataset, model statistics for the full meta-regression models with no interaction effect included.

**Table S9** Test statistics for residual heterogeneity and the omnibus test of moderators for each full meta-regression model above.

**Table S10** Model statistics for publication bias tests for the published data of our dataset.

**Tables S11-S19** For the complete dataset, model statistics for the full meta-regression models with the interaction effect between each steepness measure and the dispersal pattern of the study species, and statistics of the likelihood ratio tests comparing the full model with the respective control model.

**Tables S20-S28** For the complete dataset, model statistics for the full meta-regression models with the interaction effect between each steepness measure and the study setting, and statistics of the likelihood ratio tests comparing the full model with the respective control model.

**Tables S29-S37** For the complete dataset, model statistics for the full meta-regression models with the interaction effect between each steepness measure and the sex category of the study group, and statistics of the likelihood ratio tests comparing the full model with the respective control model.

**Tables S38-S45** For the restricted dataset including only groups in which the dominance hierarchy is significantly linear, model statistics for the full meta-regression models with no interaction effect included.

**Tables S46-S47** Model statistics for the simpler models with only the predictor (i.e., steepness) included, and statistics of the likelihood ratio tests comparing the full model with the respective control model for the complete dataset and the restricted dataset including only groups in which the dominance hierarchy is significantly linear, respectively.

**Tables S48-S55** For the published data, model statistics for the full meta-regression models with no interaction effect included.

**Tables S56-S63** For the unpublished data, model statistics for the full meta-regression models with no interaction effect included.

**Appendices 1-2** The published and unpublished data included in our dataset and the corresponding measures of fitness-related benefits used for the meta-regression analysis.


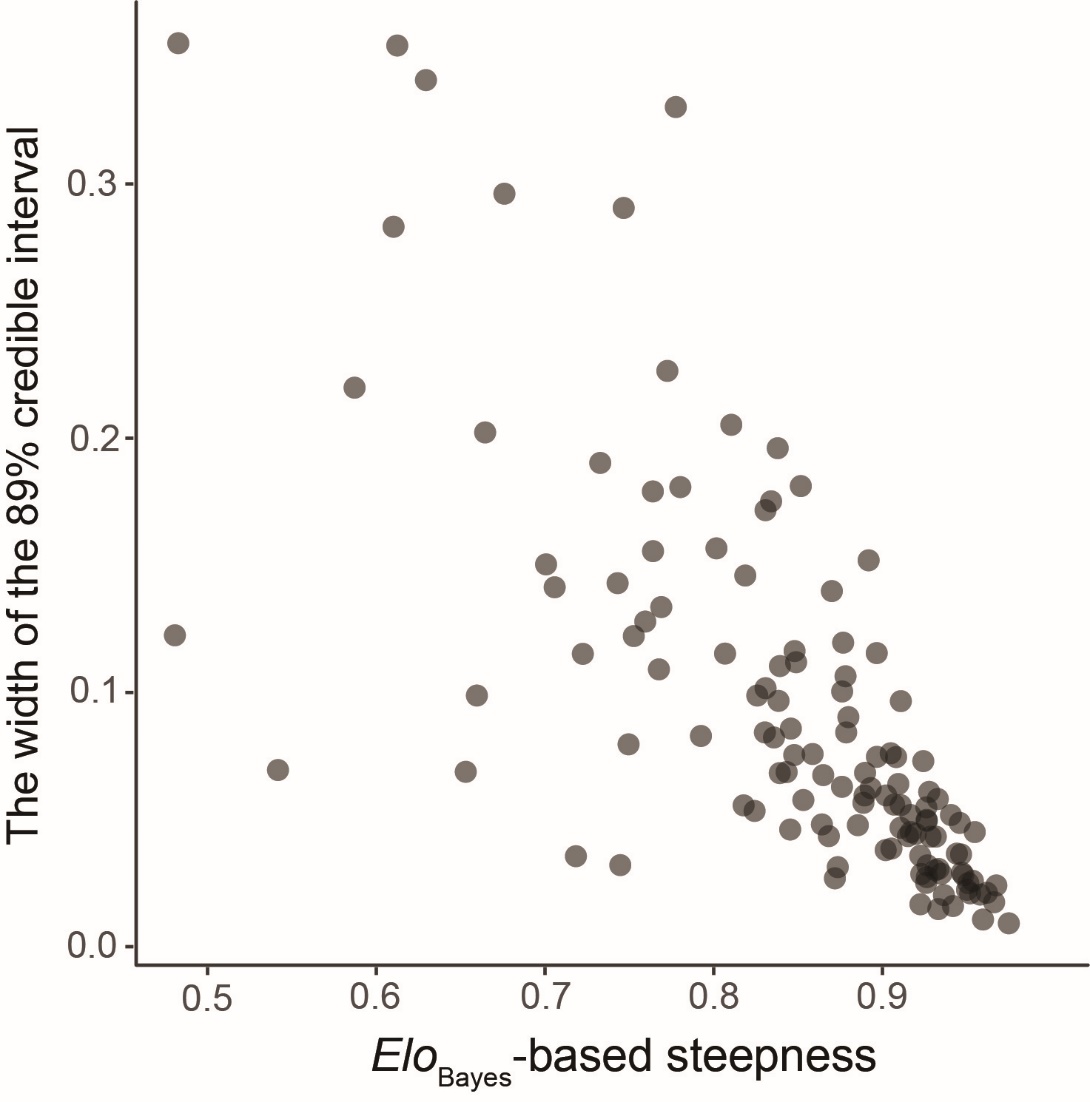


**Figure S1** The width of the 89% credible interval of *Elo*_Bayes_-based steepness we use in our dataset (n = 126). A steepness with a wide credible interval is less reliable than one with a narrow credible interval. The number of matrices in the graph is smaller than the number of data points (n = 153) because some matrices were used for more than one measure. See the details in Appendixes 1 and 2.


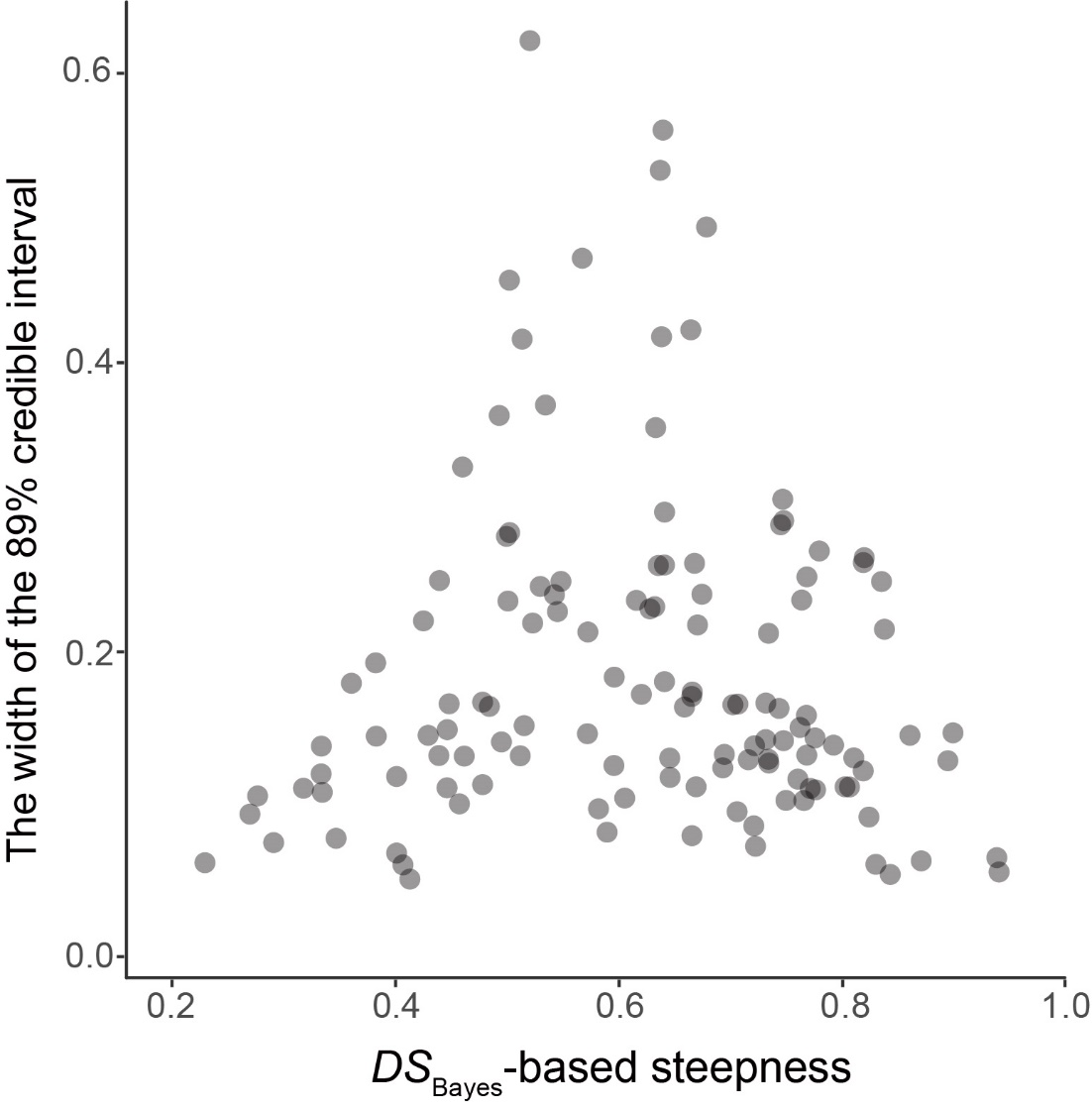


**Figure S1** The width of the 89% credible interval of *DS*_Bayes_-based steepness we use in our dataset (n = 126). A steepness with a wide credible interval is less reliable than one with a narrow credible interval. The number of matrices in the graph is smaller than the number of data points (n = 153) because some matrices were used for more than one measure. See the details in Appendixes 1 and 2.

**Table S1** Complete dataset: model statistics for the full meta-regression model showing the effect of *NDS_Dij_*-based steepness on the distribution of benefits in relation to dominance rank when the effect size is calculated using individual *NDS_Dij_* (n = 153). The phylogenetic signal (λ) of the model is less than 0.001, which is not statistically different from zero (p = 0.999).

| **Moderator** | **Estimate** | **SE** | ***Z*** | **p** | **Lower 95% CI** | **Upper 95% CI** |
| --- | --- | --- | --- | --- | --- | --- |
| intercept | 0.46 | 0.0634 | 7.2557 | <.0001*** | 0.3357 | 0.5842 |
| *NDS_Dij_*-based steepness | 0.0014 | 0.0388 | 0.0353 | 0.9719 | -0.0747 | 0.0774 |
| Study duration | -0.0238 | 0.0378 | -0.6298 | 0.5288 | -0.098 | 0.0503 |
| Benefit category_direct | 0.125 | 0.0933 | 1.3399 | 0.1803 | -0.0579 | 0.3079 |
| Dispersal pattern_FP | -0.2088 | 0.2312 | -0.903 | 0.3665 | -0.662 | 0.2444 |
| Dispersal pattern_MFP | 0.0819 | 0.283 | 0.2894 | 0.7723 | -0.4727 | 0.6365 |
| Social organization_MLS | 0.467 | 0.4288 | 1.0891 | 0.2761 | -0.3734 | 1.3075 |
| Social organization_MMG | 0.2363 | 0.221 | 1.0695 | 0.2848 | -0.1968 | 0.6695 |
| Study setting_captive | 0.3602 | 0.3903 | 0.9228 | 0.3561 | -0.4048 | 1.1251 |
| Study setting_provisioned | 0.0539 | 0.1535 | 0.3513 | 0.7254 | -0.2469 | 0.3548 |
| Sex category_female | -0.2817 | 0.0599 | -4.7058 | <.0001*** | -0.399 | -0.1644 |
| Sex category_mixed | -0.0583 | 0.1465 | -0.3981 | 0.6905 | -0.3454 | 0.2287 |
| Data origin_published | 0.4354 | 0.1755 | 2.4813 | 0.0131* | 0.0915 | 0.7792 |

**Table S2** Complete dataset: model statistics for the full meta-regression model showing the effect of *NDS_Dij_*-based steepness on the distribution of benefits in relation to dominance rank when the effect size is calculated using individual *NDS_Dij_*-based ordinal ranks (n = 153). The phylogenetic signal (λ) of the model is less than 0.001, which is not statistically different from zero (p = 0.999).

| **Moderator** | **Estimate** | **SE** | ***Z*** | **p** | **Lower 95% CI** | **Upper 95% CI** |
| --- | --- | --- | --- | --- | --- | --- |
| intercept | 0.4468 | 0.0669 | 6.6788 | <.0001*** | 0.3157 | 0.5779 |
| *NDS_Dij_*-based steepness | -0.0081 | 0.0386 | -0.2107 | 0.8331 | -0.0837 | 0.0675 |
| Study duration | -0.0205 | 0.0371 | -0.5515 | 0.5813 | -0.0932 | 0.0523 |
| Benefit category_direct | 0.1084 | 0.0919 | 1.1798 | 0.2381 | -0.0717 | 0.2886 |
| Dispersal pattern_FP | -0.2058 | 0.2347 | -0.8769 | 0.3806 | -0.6657 | 0.2542 |
| Dispersal pattern_MFP | 0.0082 | 0.2908 | 0.0281 | 0.9776 | -0.5618 | 0.5782 |
| Social organization_MLS | 0.5948 | 0.4338 | 1.3714 | 0.1703 | -0.2553 | 1.445 |
| Social organization_MMG | 0.1972 | 0.222 | 0.8883 | 0.3744 | -0.2379 | 0.6323 |
| Study setting_captive | 0.3815 | 0.3901 | 0.9779 | 0.3281 | -0.3831 | 1.1462 |
| Study setting_provisioned | 0.0784 | 0.145 | 0.5406 | 0.5888 | -0.2059 | 0.3627 |
| Sex category_female | -0.3252 | 0.0596 | -5.4578 | <.0001*** | -0.442 | -0.2084 |
| Sex category_mixed | -0.1198 | 0.1449 | -0.8267 | 0.4084 | -0.4038 | 0.1642 |
| Data origin_published | 0.4559 | 0.157 | 2.904 | 0.0037** | 0.1482 | 0.7636 |

**Table S3** Complete dataset: model statistics for the full meta-regression model showing the effect of *Elo*_rpt_-based steepness on the distribution of benefits in relation to dominance rank when the effect size is calculated using individual *Elo*_rpt_ (n = 153). The phylogenetic signal (λ) of the model is less than 0.001, which is not statistically different from zero (p = 0.999).

| **Moderator** | **Estimate** | **SE** | ***Z*** | **p** | **Lower 95% CI** | **Upper 95% CI** |
| --- | --- | --- | --- | --- | --- | --- |
| Intercept | 0.4508 | 0.0649 | 6.948 | <.0001*** | 0.3237 | 0.578 |
| *Elo*_rpt_-based steepness | 0.0643 | 0.0386 | 1.6676 | 0.0954. | -0.0113 | 0.14 |
| Study duration | -0.0134 | 0.0354 | -0.3794 | 0.7044 | -0.0828 | 0.0559 |
| Benefit category_direct | 0.1217 | 0.0904 | 1.3468 | 0.1781 | -0.0554 | 0.2988 |
| Dispersal pattern_FP | -0.2047 | 0.2346 | -0.8725 | 0.3829 | -0.6645 | 0.2551 |
| Dispersal pattern_MFP | -0.0691 | 0.2864 | -0.2414 | 0.8093 | -0.6304 | 0.4922 |
| Social organization_MLS | 0.6591 | 0.4335 | 1.5205 | 0.1284 | -0.1905 | 1.5088 |
| Social organization_MMG | 0.2081 | 0.2212 | 0.9409 | 0.3468 | -0.2254 | 0.6416 |
| Study setting_captive | 0.4779 | 0.3881 | 1.2314 | 0.2182 | -0.2828 | 1.2387 |
| Study setting_provisioned | 0.1217 | 0.1557 | 0.7818 | 0.4343 | -0.1834 | 0.4269 |
| Sex category_female | -0.3091 | 0.058 | -5.3286 | <.0001*** | -0.4228 | -0.1954 |
| Sex category_mixed | -0.0934 | 0.1452 | -0.6436 | 0.5199 | -0.378 | 0.1911 |
| Data origin_published | 0.4829 | 0.1764 | 2.7373 | 0.0062** | 0.1371 | 0.8286 |

**Table S4** Complete dataset: model statistics for the full meta-regression model showing the effect of *Elo*_rpt_-based steepness on the distribution of benefits in relation to dominance rank when the effect size is calculated using individual *Elo*_rpt_-based ordinal ranks (n = 153). The phylogenetic signal (λ) of the model is less than 0.001, which is not statistically different from zero (p = 0.999).

| **Moderator** | **Estimate** | **SE** | ***Z*** | **p** | **Lower 95% CI** | **Upper 95% CI** |
| --- | --- | --- | --- | --- | --- | --- |
| Intercept | 0.4582 | 0.0613 | 7.4814 | <.0001*** | 0.3382 | 0.5783 |
| *Elo*_rpt_-based steepness | 0.0546 | 0.0383 | 1.423 | 0.1547 | -0.0206 | 0.1297 |
| Study duration | -0.0183 | 0.0352 | -0.5198 | 0.6032 | -0.0872 | 0.0506 |
| Benefit category_direct | 0.1349 | 0.0897 | 1.5037 | 0.1326 | -0.0409 | 0.3107 |
| Dispersal pattern_FP | -0.2325 | 0.2264 | -1.027 | 0.3044 | -0.6762 | 0.2112 |
| Dispersal pattern_MFP | -0.0537 | 0.2777 | -0.1935 | 0.8465 | -0.598 | 0.4905 |
| Social organization_MLS | 0.6255 | 0.4247 | 1.473 | 0.1408 | -0.2068 | 1.4579 |
| Social organization_MMG | 0.2165 | 0.2172 | 0.9964 | 0.319 | -0.2093 | 0.6422 |
| Study setting_captive | 0.3929 | 0.384 | 1.0231 | 0.3063 | -0.3598 | 1.1455 |
| Study setting_provisioned | 0.0958 | 0.1503 | 0.6375 | 0.5238 | -0.1987 | 0.3903 |
| Sex category_female | -0.3134 | 0.0579 | -5.4129 | <.0001*** | -0.4269 | -0.1999 |
| Sex category_mixed | -0.1098 | 0.1446 | -0.7591 | 0.4478 | -0.3932 | 0.1737 |
| Data origin_published | 0.4612 | 0.1693 | 2.7235 | 0.0065** | 0.1293 | 0.7931 |

**Table S5** Complete dataset: model statistics for the full meta-regression model showing the effect of *Elo*_Bayes_-based steepness on the distribution of benefits in relation to dominance rank when the effect size is calculated using individual *Elo*_Bayes_ (n = 153). The phylogenetic signal (λ) of the model is less than 0.001, which is not statistically different from zero (p = 0.999).

| **Moderator** | **Estimate** | **SE** | ***Z*** | **p** | **Lower 95% CI** | **Upper 95% CI** |
| --- | --- | --- | --- | --- | --- | --- |
| intercept | 0.4594 | 0.0613 | 7.4882 | <.0001*** | 0.3391 | 0.5796 |
| *Elo*_Bayes_-based steepness | 0.0386 | 0.0381 | 1.0118 | 0.3117 | -0.0361 | 0.1133 |
| Study duration | -0.0234 | 0.0357 | -0.6572 | 0.511 | -0.0933 | 0.0465 |
| Benefit category_direct | 0.1136 | 0.0911 | 1.2465 | 0.2126 | -0.065 | 0.2921 |
| Dispersal pattern_FP | -0.2685 | 0.2265 | -1.1851 | 0.236 | -0.7124 | 0.1755 |
| Dispersal pattern_MFP | 0.0558 | 0.279 | 0.2001 | 0.8414 | -0.491 | 0.6027 |
| Social organization_MLS | 0.4227 | 0.4235 | 0.9982 | 0.3182 | -0.4073 | 1.2527 |
| Social organization_MMG | 0.2314 | 0.2178 | 1.0622 | 0.2882 | -0.1956 | 0.6583 |
| Study setting_captive | 0.4259 | 0.3843 | 1.1082 | 0.2678 | -0.3274 | 1.1792 |
| Study setting_provisioned | 0.0629 | 0.1505 | 0.4184 | 0.6757 | -0.232 | 0.3579 |
| Sex category_female | -0.2961 | 0.0583 | -5.082 | <.0001*** | -0.4103 | -0.1819 |
| Sex category_mixed | -0.1151 | 0.1458 | -0.7896 | 0.4298 | -0.4008 | 0.1706 |
| Data origin_published | 0.4414 | 0.1697 | 2.6008 | 0.0093** | 0.1088 | 0.774 |

**Table S6** Complete dataset: model statistics for the full meta-regression model showing the effect of *Elo*_Bayes_-based steepness on the distribution of benefits in relation to dominance rank when the effect size is calculated using individual *Elo*_Bayes_-based ordinal ranks (n = 153). The phylogenetic signal (λ) of the model is less than 0.001, which is not statistically different from zero (p = 0.999).

| **Moderator** | **Estimate** | **SE** | ***Z*** | **p** | **Lower 95% CI** | **Upper 95% CI** |
| --- | --- | --- | --- | --- | --- | --- |
| intercept | 0.4515 | 0.061 | 7.4032 | <.0001*** | 0.332 | 0.571 |
| *Elo*_Bayes_-based steepness | 0.037 | 0.0381 | 0.9708 | 0.3316 | -0.0377 | 0.1117 |
| Study duration | -0.0259 | 0.0357 | -0.7266 | 0.4675 | -0.0959 | 0.044 |
| Benefit category_direct | 0.134 | 0.0912 | 1.4702 | 0.1415 | -0.0446 | 0.3127 |
| Dispersal pattern_FP | -0.2548 | 0.2258 | -1.1288 | 0.259 | -0.6973 | 0.1876 |
| Dispersal pattern_MFP | -0.0051 | 0.2782 | -0.0185 | 0.9852 | -0.5504 | 0.5401 |
| Social organization_MLS | 0.53 | 0.4228 | 1.2535 | 0.21 | -0.2987 | 1.3587 |
| Social organization_MMG | 0.2123 | 0.2175 | 0.9757 | 0.3292 | -0.2141 | 0.6386 |
| Study setting_captive | 0.3395 | 0.3841 | 0.8839 | 0.3768 | -0.4133 | 1.0922 |
| Study setting_provisioned | 0.0896 | 0.15 | 0.5975 | 0.5502 | -0.2044 | 0.3836 |
| Sex category_female | -0.3069 | 0.0583 | -5.2671 | <.0001*** | -0.4211 | -0.1927 |
| Sex category_mixed | -0.1081 | 0.1458 | -0.7416 | 0.4583 | -0.3938 | 0.1776 |
| Data origin_published | 0.4522 | 0.1691 | 2.6742 | 0.0075** | 0.1208 | 0.7836 |

**Table S7** Complete dataset: model statistics for the full meta-regression model showing the effect of *DS*_Bayes_-based steepness on the distribution of benefits in relation to dominance rank when the effect size is calculated using individual *DS*_Bayes_ (n = 153). The phylogenetic signal (λ) of the model is less than 0.001, which is not statistically different from zero (p = 0.999).

| **Moderator** | **Estimate** | **SE** | ***Z*** | **p** | **Lower 95% CI** | **Upper 95% CI** |
| --- | --- | --- | --- | --- | --- | --- |
| intercept | 0.4533 | 0.0612 | 7.4073 | <.0001*** | 0.3334 | 0.5733 |
| *DS*_Bayes_-based steepness | 0.0018 | 0.0384 | 0.0473 | 0.9623 | -0.0734 | 0.0771 |
| Study duration | -0.0186 | 0.0377 | -0.4921 | 0.6227 | -0.0925 | 0.0554 |
| Benefit category_direct | 0.0771 | 0.0953 | 0.809 | 0.4185 | -0.1097 | 0.2639 |
| Dispersal pattern_FP | -0.2463 | 0.2263 | -1.0883 | 0.2765 | -0.6899 | 0.1973 |
| Dispersal pattern_MFP | 0.1116 | 0.2781 | 0.4012 | 0.6883 | -0.4334 | 0.6566 |
| Social organization_MLS | 0.4695 | 0.4252 | 1.1044 | 0.2694 | -0.3638 | 1.3028 |
| Social organization_MMG | 0.2745 | 0.2195 | 1.2508 | 0.211 | -0.1556 | 0.7047 |
| Study setting_captive | 0.3286 | 0.3881 | 0.8467 | 0.3972 | -0.432 | 1.0892 |
| Study setting_provisioned | 0.043 | 0.1509 | 0.2851 | 0.7756 | -0.2527 | 0.3387 |
| Sex category_female | -0.2641 | 0.0595 | -4.4412 | <.0001*** | -0.3807 | -0.1476 |
| Sex category_mixed | -0.0774 | 0.1489 | -0.52 | 0.6031 | -0.3692 | 0.2144 |
| Data origin_published | 0.4085 | 0.1711 | 2.3881 | 0.0169* | 0.0732 | 0.7438 |

**Table S8** Complete dataset: model statistics for the full meta-regression model showing the effect of *DS*_Bayes_-based steepness on the distribution of benefits in relation to dominance rank when the effect size is calculated using individual *DS*_Bayes_-based ordinal ranks (n = 153). The phylogenetic signal (λ) of the model is less than 0.001, which is not statistically different from zero (p = 0.999).

| **Moderator** | **Estimate** | **SE** | ***Z*** | **p** | **Lower 95% CI** | **Upper 95% CI** |
| --- | --- | --- | --- | --- | --- | --- |
| intercept | 0.4321 | 0.0625 | 6.9101 | <.0001*** | 0.3095 | 0.5546 |
| *DS*_Bayes_-based steepness | -0.006 | 0.0381 | -0.1578 | 0.8746 | -0.0807 | 0.0687 |
| Study duration | -0.0214 | 0.0372 | -0.5747 | 0.5655 | -0.0944 | 0.0516 |
| Benefit category_direct | 0.0899 | 0.0945 | 0.9518 | 0.3412 | -0.0953 | 0.2752 |
| Dispersal pattern_FP | -0.2694 | 0.2288 | -1.1772 | 0.2391 | -0.7178 | 0.1791 |
| Dispersal pattern_MFP | -0.021 | 0.2808 | -0.0747 | 0.9405 | -0.5712 | 0.5293 |
| Social organization_MLS | 0.611 | 0.4261 | 1.4339 | 0.1516 | -0.2242 | 1.4462 |
| Social organization_MMG | 0.1961 | 0.2198 | 0.892 | 0.3724 | -0.2347 | 0.6269 |
| Study setting_captive | 0.3517 | 0.3881 | 0.9062 | 0.3648 | -0.409 | 1.1124 |
| Study setting_provisioned | 0.0576 | 0.1521 | 0.3787 | 0.7049 | -0.2406 | 0.3558 |
| Sex category_female | -0.2922 | 0.0594 | -4.9227 | <.0001*** | -0.4085 | -0.1758 |
| Sex category_mixed | -0.1094 | 0.1487 | -0.7356 | 0.462 | -0.4009 | 0.1821 |
| Data origin_published | 0.4305 | 0.1729 | 2.4907 | 0.0128* | 0.0917 | 0.7693 |

**Table S9** Test statistics of the test for residual heterogeneity and the omnibus test of moderators for each full model assessing the influence of hierarchy steepness on the distribution of benefits among group members in relation to dominance rank (n = 153).

| **Dominance rank measure** | **Q_M_** | **p (Q_M_)** | **Q_E_^a^** | **p (Q_E_)** |
| --- | --- | --- | --- | --- |
| *NDS_Dij_* | 58.3967 | <.0001 | 215.2961 | <.0001 |
| *NDS_Dij_*-based ordinal rank | 68.1820 | <.0001 | 220.2178 | <.0001 |
| *Elo*_rpt_ | 62.5431 | <.0001 | 214.5434 | <.0001 |
| *Elo*_rpt_-based ordinal rank | 65.7086 | <.0001 | 212.2932 | <.0001 |
| *Elo*_Bayes_ | 62.7007 | <.0001 | 216.5926 | <.0001 |
| *Elo*_Bayes_-based ordinal rank | 63.8002 | <.0001 | 213.2041 | <.0001 |
| *DS*_Bayes_ | 55.8741 | <.0001 | 220.6867 | <.0001 |
| *DS*_Bayes_-based ordinal rank | 61.0724 | <.0001 | 217.1155 | <.0001 |

^a^ Test for residual heterogeneity: significant values indicating the existence of unmeasured variables contributing to the unexplained heterogeneity in the full model.

**Table S10** Model statistics for the uni-moderator meta-regression models examining the small-study effect and the time-lag effect in the published data of our dataset (n = 75).

| **Dominance rank measure** | **Moderator** | **Estimate** | **SE** | ***Z*** | **p** | **Lower 95% CI** | **Upper 95% CI** |
| --- | --- | --- | --- | --- | --- | --- | --- |
| *NDS_Dij_* | Sample size | 2.6273 | 1.0954 | 2.3984 | 0.0165* | 0.4802 | 4.7743 |
|  | Year | 0.03 | 0.0866 | 0.3465 | 0.729 | -0.1397 | 0.1996 |
| *NDS_Dij_*-based ordinal rank | Sample size | 2.109 | 1.0421 | 2.0238 | 0.043* | 0.0665 | 4.1516 |
|  | Year | 0.047 | 0.0789 | 0.5949 | 0.5519 | -0.1078 | 0.2017 |
| *Elo*_rpt_ | Sample size | 2.4059 | 1.0998 | 2.1876 | 0.0287* | 0.2503 | 4.5615 |
|  | Year | 0.0424 | 0.086 | 0.4933 | 0.6218 | -0.1262 | 0.2111 |
| *Elo*_rpt_-based ordinal rank | Sample size | 2.3801 | 1.0877 | 2.1882 | 0.0287* | 0.2482 | 4.5119 |
|  | Year | 0.0403 | 0.0837 | 0.4815 | 0.6301 | -0.1238 | 0.2045 |
| *Elo*_Bayes_ | Sample size | 2.8416 | 1.1052 | 2.5711 | 0.0101* | 0.6755 | 5.0078 |
|  | Year | 0.0255 | 0.0879 | 0.2905 | 0.7715 | -0.1468 | 0.1979 |
| *Elo*_Bayes_-based ordinal rank | Sample size | 2.4548 | 1.0848 | 2.263 | 0.0236* | 0.3287 | 4.5809 |
|  | Year | 0.0535 | 0.0837 | 0.6391 | 0.5228 | -0.1105 | 0.2175 |
| *DS*_Bayes_ | Sample size | 2.6846 | 1.0998 | 2.4411 | 0.0146* | 0.5292 | 4.8401 |
|  | Year | 0.0392 | 0.0881 | 0.4444 | 0.6568 | -0.1336 | 0.2119 |
| *DS*_Bayes_-based ordinal rank | Sample size | 2.4416 | 1.0961 | 2.2274 | 0.0259* | 0.2932 | 4.59 |
|  | Year | 0.0571 | 0.0847 | 0.6739 | 0.5004 | -0.1089 | 0.2231 |

**Table S11** Complete dataset: model statistics for the full meta-regression model showing the effect of the dispersal pattern of the study species on the relationship between *NDS_Dij_*-based steepness and the distribution of benefits in relation to dominance rank when the effect size is calculated using individual *NDS_Dij_* (n = 153). The interaction between the two variables is represented by the multiplication sign (×) between the variable names. The phylogenetic signal (λ) of the model is less than 0.001, which is not statistically different from zero (p = 0.999).

| **Moderator** | **Estimate** | **SE** | ***Z*** | **p** | **Lower 95% CI** | **Upper 95% CI** |
| --- | --- | --- | --- | --- | --- | --- |
| intercept | 0.4575 | 0.0647 | 7.0747 | <.0001*** | 0.3308 | 0.5843 |
| *NDS_Dij_*-based steepness | 0.003 | 0.0399 | 0.0758 | 0.9396 | -0.0751 | 0.0812 |
| Study duration | -0.0235 | 0.038 | -0.6178 | 0.5367 | -0.098 | 0.051 |
| Benefit category_direct | 0.124 | 0.0937 | 1.3225 | 0.186 | -0.0598 | 0.3077 |
| Dispersal pattern_FP | -0.205 | 0.2329 | -0.8803 | 0.3787 | -0.6614 | 0.2514 |
| Dispersal pattern_MFP | 0.079 | 0.2842 | 0.2781 | 0.781 | -0.478 | 0.636 |
| Social organization_MLS | 0.4696 | 0.4304 | 1.0909 | 0.2753 | -0.3741 | 1.3132 |
| Social organization_MMG | 0.2385 | 0.2221 | 1.074 | 0.2828 | -0.1968 | 0.6738 |
| Study setting_captive | 0.3449 | 0.3976 | 0.8677 | 0.3856 | -0.4342 | 1.1241 |
| Study setting_provisioned | 0.0504 | 0.1554 | 0.3242 | 0.7458 | -0.2541 | 0.3549 |
| Sex category_female | -0.2824 | 0.0599 | -4.7138 | <.0001*** | -0.3999 | -0.165 |
| Sex category_mixed | -0.0639 | 0.1482 | -0.4312 | 0.6663 | -0.3543 | 0.2265 |
| Data origin_published | 0.4351 | 0.1765 | 2.4652 | 0.0137* | 0.0892 | 0.7811 |
| *NDS_Dij_*-based steepness × Dispersal pattern_FP | -0.025 | 0.1139 | -0.2194 | 0.8263 | -0.2482 | 0.1982 |

**Table S12** Complete dataset: model statistics for the full meta-regression model showing the effect of the dispersal pattern of the study species on the relationship between *NDS_Dij_*-based steepness on the distribution of benefits in relation to dominance rank when the effect size is calculated using individual *NDS_Dij_*-based ordinal ranks (n = 153). The interaction between the two variables is represented by the multiplication sign (×) between the variable names. The phylogenetic signal (λ) of the model is less than 0.001, which is not statistically different from zero (p = 0.999).

| **Moderator** | **Estimate** | **SE** | ***Z*** | **p** | **Lower 95% CI** | **Upper 95% CI** |
| --- | --- | --- | --- | --- | --- | --- |
| intercept | 0.4467 | 0.0682 | 6.5475 | <.0001*** | 0.313 | 0.5804 |
| *NDS_Dij_*-based steepness | -0.0086 | 0.0397 | -0.2162 | 0.8288 | -0.0864 | 0.0692 |
| Study duration | -0.0205 | 0.0373 | -0.551 | 0.5816 | -0.0936 | 0.0525 |
| Benefit category_direct | 0.1085 | 0.0923 | 1.1755 | 0.2398 | -0.0724 | 0.2893 |
| Dispersal pattern_FP | -0.2061 | 0.2366 | -0.871 | 0.3837 | -0.6698 | 0.2577 |
| Dispersal pattern_MFP | 0.0071 | 0.2921 | 0.0242 | 0.9807 | -0.5655 | 0.5796 |
| Social organization_MLS | 0.5942 | 0.4354 | 1.3647 | 0.1723 | -0.2592 | 1.4475 |
| Social organization_MMG | 0.196 | 0.2231 | 0.8786 | 0.3796 | -0.2413 | 0.6333 |
| Study setting_captive | 0.3829 | 0.3964 | 0.9658 | 0.3341 | -0.3941 | 1.1599 |
| Study setting_provisioned | 0.079 | 0.1466 | 0.5385 | 0.5902 | -0.2084 | 0.3664 |
| Sex category_female | -0.3256 | 0.0596 | -5.4598 | <.0001*** | -0.4425 | -0.2087 |
| Sex category_mixed | -0.1201 | 0.1468 | -0.818 | 0.4134 | -0.4078 | 0.1676 |
| Data origin_published | 0.4566 | 0.1577 | 2.895 | 0.0038** | 0.1475 | 0.7658 |
| *NDS_Dij_*-based steepness × Dispersal pattern_FP | 0.0016 | 0.114 | 0.0143 | 0.9886 | -0.2218 | 0.2251 |

**Table S13** Complete dataset: model statistics for the full meta-regression model showing the effect of the dispersal pattern of the study species on the relationship between *Elo*_rpt_-based steepness on the distribution of benefits in relation to dominance rank when the effect size is calculated using individual *Elo*_rpt_ (n = 153). The interaction between the two variables is represented by the multiplication sign (×) between the variable names. The phylogenetic signal (λ) of the model is less than 0.001, which is not statistically different from zero (p = 0.999).

| **Moderator** | **Estimate** | **SE** | ***Z*** | **p** | **Lower 95% CI** | **Upper 95% CI** |
| --- | --- | --- | --- | --- | --- | --- |
| Intercept | 0.4569 | 0.0654 | 6.981 | <.0001*** | 0.3286 | 0.5852 |
| *Elo*_rpt_-based steepness | 0.0852 | 0.0415 | 2.0558 | 0.0398* | 0.004 | 0.1665 |
| Study duration | -0.0151 | 0.0355 | -0.4253 | 0.6706 | -0.0846 | 0.0544 |
| Benefit category_direct | 0.1151 | 0.0906 | 1.2707 | 0.2038 | -0.0624 | 0.2927 |
| Dispersal pattern_FP | -0.1458 | 0.2393 | -0.6091 | 0.5424 | -0.6149 | 0.3233 |
| Dispersal pattern_MFP | 0.0008 | 0.2919 | 0.0027 | 0.9979 | -0.5713 | 0.5729 |
| Social organization_MLS | 0.8668 | 0.4597 | 1.8856 | 0.0593 | -0.0342 | 1.7678 |
| Social organization_MMG | 0.2361 | 0.2226 | 1.0606 | 0.2889 | -0.2002 | 0.6725 |
| Study setting_captive | 0.5375 | 0.391 | 1.3747 | 0.1692 | -0.2288 | 1.3037 |
| Study setting_provisioned | 0.1519 | 0.1577 | 0.9628 | 0.3357 | -0.1573 | 0.461 |
| Sex category_female | -0.3066 | 0.058 | -5.2826 | <.0001*** | -0.4204 | -0.1929 |
| Sex category_mixed | -0.0779 | 0.1457 | -0.5348 | 0.5928 | -0.3635 | 0.2077 |
| Data origin_published | 0.5083 | 0.1781 | 2.854 | 0.0043** | 0.1592 | 0.8573 |
| *Elo*_rpt_-based steepness × Dispersal pattern_FP | -0.1892 | 0.136 | -1.3912 | 0.1642 | -0.4557 | 0.0773 |

**Table S14** Complete dataset: model statistics for the full meta-regression model showing the effect of the dispersal pattern of the study species on the relationship between *Elo*_rpt_-based steepness on the distribution of benefits in relation to dominance rank when the effect size is calculated using individual *Elo*_rpt_-based ordinal ranks (n = 153). The interaction between the two variables is represented by the multiplication sign (×) between the variable names. The phylogenetic signal (λ) of the model is less than 0.001, which is not statistically different from zero (p = 0.999).

| **Moderator** | **Estimate** | **SE** | ***Z*** | **p** | **Lower 95% CI** | **Upper 95% CI** |
| --- | --- | --- | --- | --- | --- | --- |
| Intercept | 0.4646 | 0.0619 | 7.5059 | <.0001*** | 0.3433 | 0.586 |
| *Elo*_rpt_-based steepness | 0.0777 | 0.0412 | 1.8888 | 0.0589. | -0.0029 | 0.1584 |
| Study duration | -0.0201 | 0.0352 | -0.5709 | 0.5681 | -0.0891 | 0.0489 |
| Benefit category_direct | 0.1277 | 0.09 | 1.4199 | 0.1556 | -0.0486 | 0.304 |
| Dispersal pattern_FP | -0.168 | 0.2312 | -0.7264 | 0.4676 | -0.6212 | 0.2852 |
| Dispersal pattern_MFP | 0.0223 | 0.2832 | 0.0787 | 0.9373 | -0.5328 | 0.5774 |
| Social organization_MLS | 0.855 | 0.4505 | 1.8976 | 0.0577 | -0.0281 | 1.738 |
| Social organization_MMG | 0.2469 | 0.2187 | 1.1291 | 0.2589 | -0.1817 | 0.6756 |
| Study setting_captive | 0.4572 | 0.3868 | 1.1821 | 0.2372 | -0.3009 | 1.2152 |
| Study setting_provisioned | 0.1271 | 0.1523 | 0.8346 | 0.4039 | -0.1714 | 0.4256 |
| Sex category_female | -0.3106 | 0.058 | -5.3602 | <.0001*** | -0.4242 | -0.1971 |
| Sex category_mixed | -0.0926 | 0.1451 | -0.6381 | 0.5234 | -0.3771 | 0.1919 |
| Data origin_published | 0.488 | 0.1711 | 2.8515 | 0.0044** | 0.1526 | 0.8234 |
| *Elo*_rpt_-based steepness × Dispersal pattern_FP | -0.2112 | 0.1345 | -1.57 | 0.1164 | -0.4748 | 0.0525 |

**Table S15** Complete dataset: model statistics for the full meta-regression model showing the effect of the dispersal pattern of the study species on the relationship between *Elo*_Bayes_-based steepness on the distribution of benefits in relation to dominance rank when the effect size is calculated using individual *Elo*_Bayes_ (n = 153). The interaction between the two variables is represented by the multiplication sign (×) between the variable names. The phylogenetic signal (λ) of the model is less than 0.001, which is not statistically different from zero (p = 0.999).

| **Moderator** | **Estimate** | **SE** | ***Z*** | **p** | **Lower 95% CI** | **Upper 95% CI** |
| --- | --- | --- | --- | --- | --- | --- |
| intercept | 0.4791 | 0.061 | 7.8544 | <.0001*** | 0.3596 | 0.5987 |
| *Elo*_Bayes_-based steepness | 0.0642 | 0.0425 | 1.5105 | 0.1309 | -0.0191 | 0.1475 |
| Study duration | -0.0287 | 0.036 | -0.7964 | 0.4258 | -0.0993 | 0.0419 |
| Benefit category_direct | 0.1058 | 0.0915 | 1.1555 | 0.2479 | -0.0736 | 0.2852 |
| Dispersal pattern_FP | -0.2399 | 0.2234 | -1.0735 | 0.2831 | -0.6778 | 0.1981 |
| Dispersal pattern_MFP | 0.2151 | 0.2976 | 0.723 | 0.4697 | -0.3681 | 0.7984 |
| Social organization_MLS | 0.5104 | 0.4247 | 1.2019 | 0.2294 | -0.3219 | 1.3427 |
| Social organization_MMG | 0.2977 | 0.2214 | 1.3449 | 0.1787 | -0.1361 | 0.7315 |
| Study setting_captive | 0.4021 | 0.3831 | 1.0496 | 0.2939 | -0.3488 | 1.1531 |
| Study setting_provisioned | 0.0456 | 0.1481 | 0.3079 | 0.7582 | -0.2447 | 0.3359 |
| Sex category_female | -0.2916 | 0.0584 | -4.9965 | <.0001*** | -0.406 | -0.1772 |
| Sex category_mixed | -0.1054 | 0.1458 | -0.7228 | 0.4698 | -0.3911 | 0.1803 |
| Data origin_published | 0.4315 | 0.1664 | 2.5938 | 0.0095** | 0.1054 | 0.7576 |
| *Elo*_Bayes_-based steepness × Dispersal pattern_FP | -0.1982 | 0.1487 | -1.3324 | 0.1827 | -0.4896 | 0.0933 |

**Table S16** Complete dataset: model statistics for the full meta-regression model showing the effect of the dispersal pattern of the study species on the relationship between *Elo*_Bayes_-based steepness on the distribution of benefits in relation to dominance rank when the effect size is calculated using individual *Elo*_Bayes_-based ordinal ranks (n = 153). The interaction between the two variables is represented by the multiplication sign (×) between the variable names. The phylogenetic signal (λ) of the model is less than 0.001, which is not statistically different from zero (p = 0.999).

| **Moderator** | **Estimate** | **SE** | ***Z*** | **p** | **Lower 95% CI** | **Upper 95% CI** |
| --- | --- | --- | --- | --- | --- | --- |
| intercept | 0.4693 | 0.0613 | 7.6554 | <.0001*** | 0.3492 | 0.5895 |
| *Elo*_Bayes_-based steepness | 0.0606 | 0.0425 | 1.4235 | 0.1546 | -0.0228 | 0.1439 |
| Study duration | -0.0308 | 0.0361 | -0.8552 | 0.3924 | -0.1015 | 0.0398 |
| Benefit category_direct | 0.127 | 0.0916 | 1.386 | 0.1657 | -0.0526 | 0.3065 |
| Dispersal pattern_FP | -0.2284 | 0.2241 | -1.0192 | 0.3081 | -0.6677 | 0.2109 |
| Dispersal pattern_MFP | 0.1398 | 0.2983 | 0.4686 | 0.6394 | -0.4449 | 0.7245 |
| Social organization_MLS | 0.6103 | 0.4254 | 1.4346 | 0.1514 | -0.2235 | 1.4441 |
| Social organization_MMG | 0.2721 | 0.2217 | 1.2273 | 0.2197 | -0.1624 | 0.7066 |
| Study setting_captive | 0.319 | 0.3835 | 0.8319 | 0.4055 | -0.4326 | 1.0706 |
| Study setting_provisioned | 0.0744 | 0.1486 | 0.5009 | 0.6164 | -0.2168 | 0.3657 |
| Sex category_female | -0.3029 | 0.0584 | -5.1894 | <.0001*** | -0.4173 | -0.1885 |
| Sex category_mixed | -0.0999 | 0.1458 | -0.685 | 0.4934 | -0.3857 | 0.1859 |
| Data origin_published | 0.4443 | 0.167 | 2.6611 | 0.0078** | 0.1171 | 0.7716 |
| *Elo*_Bayes_-based steepness × Dispersal pattern_FP | -0.1831 | 0.1489 | -1.2298 | 0.2188 | -0.4749 | 0.1087 |

**Table S17** Complete dataset: model statistics for the full meta-regression model showing the effect of the dispersal pattern of the study species on the relationship between *DS*_Bayes_-based steepness on the distribution of benefits in relation to dominance rank when the effect size is calculated using individual *DS*_Bayes_ (n = 153). The interaction between the two variables is represented by the multiplication sign (×) between the variable names. The phylogenetic signal (λ) of the model is less than 0.001, which is not statistically different from zero (p = 0.999).

| **Moderator** | **Estimate** | **SE** | ***Z*** | **p** | **Lower 95% CI** | **Upper 95% CI** |
| --- | --- | --- | --- | --- | --- | --- |
| intercept | 0.4446 | 0.0629 | 7.0729 | <.0001*** | 0.3214 | 0.5678 |
| *DS*_Bayes_-based steepness | 0.0125 | 0.0427 | 0.2926 | 0.7698 | -0.0711 | 0.0961 |
| Study duration | -0.0184 | 0.0379 | -0.4865 | 0.6266 | -0.0927 | 0.0559 |
| Benefit category_direct | 0.0755 | 0.0956 | 0.7897 | 0.4297 | -0.1119 | 0.263 |
| Dispersal pattern_FP | -0.2135 | 0.2326 | -0.918 | 0.3586 | -0.6693 | 0.2423 |
| Dispersal pattern_MFP | 0.1128 | 0.2773 | 0.4067 | 0.6842 | -0.4307 | 0.6562 |
| Social organization_MLS | 0.4842 | 0.4257 | 1.1375 | 0.2553 | -0.3501 | 1.3185 |
| Social organization_MMG | 0.2815 | 0.2197 | 1.281 | 0.2002 | -0.1492 | 0.7121 |
| Study setting_captive | 0.2948 | 0.3923 | 0.7516 | 0.4523 | -0.474 | 1.0637 |
| Study setting_provisioned | 0.042 | 0.1505 | 0.2792 | 0.7801 | -0.2529 | 0.337 |
| Sex category_female | -0.2629 | 0.0596 | -4.415 | <.0001*** | -0.3797 | -0.1462 |
| Sex category_mixed | -0.0874 | 0.1501 | -0.5823 | 0.5604 | -0.3816 | 0.2068 |
| Data origin_published | 0.411 | 0.1707 | 2.408 | 0.016* | 0.0765 | 0.7455 |
| *DS*_Bayes_-based steepness × Dispersal pattern_FP | -0.0811 | 0.1424 | -0.5694 | 0.5691 | -0.3601 | 0.198 |

**Table S18** Complete dataset: model statistics for the full meta-regression model showing the effect of the dispersal pattern of the study species on the relationship between *DS*_Bayes_-based steepness on the distribution of benefits in relation to dominance rank when the effect size is calculated using individual *DS*_Bayes_-based ordinal ranks (n = 153). The interaction between the two variables is represented by the multiplication sign (×) between the variable names. The phylogenetic signal (λ) of the model is less than 0.001, which is not statistically different from zero (p = 0.999).

| **Moderator** | **Estimate** | **SE** | ***Z*** | **p** | **Lower 95% CI** | **Upper 95% CI** |
| --- | --- | --- | --- | --- | --- | --- |
| intercept | 0.4304 | 0.065 | 6.6266 | <.0001*** | 0.3031 | 0.5577 |
| *DS*_Bayes_-based steepness | -0.0047 | 0.0425 | -0.1107 | 0.9118 | -0.088 | 0.0786 |
| Study duration | -0.0214 | 0.0373 | -0.5727 | 0.5669 | -0.0946 | 0.0518 |
| Benefit category_direct | 0.0895 | 0.0948 | 0.9449 | 0.3447 | -0.0962 | 0.2753 |
| Dispersal pattern_FP | -0.2642 | 0.2369 | -1.1151 | 0.2648 | -0.7286 | 0.2002 |
| Dispersal pattern_MFP | -0.022 | 0.2818 | -0.0781 | 0.9377 | -0.5743 | 0.5303 |
| Social organization_MLS | 0.6128 | 0.4281 | 1.4316 | 0.1523 | -0.2262 | 1.4518 |
| Social organization_MMG | 0.1965 | 0.2207 | 0.8907 | 0.3731 | -0.2359 | 0.629 |
| Study setting_captive | 0.3474 | 0.3926 | 0.8849 | 0.3762 | -0.4221 | 1.1169 |
| Study setting_provisioned | 0.0582 | 0.1528 | 0.3809 | 0.7033 | -0.2414 | 0.3578 |
| Sex category_female | -0.2924 | 0.0594 | -4.9194 | <.0001*** | -0.4089 | -0.1759 |
| Sex category_mixed | -0.1119 | 0.15 | -0.7459 | 0.4557 | -0.4059 | 0.1821 |
| Data origin_published | 0.4322 | 0.1739 | 2.485 | 0.013* | 0.0913 | 0.7731 |
| *DS*_Bayes_-based steepness × Dispersal pattern_FP | -0.0126 | 0.1426 | -0.0883 | 0.9296 | -0.2922 | 0.267 |

**Table S19** Test statistics of the likelihood ratio tests comparing each full model, in which the interaction effect between steepness and the dispersal pattern of the study species is included, with the respective control model. The degrees of freedom of each full model and the respective model were 17 and 13, respectively.

| **Dominance rank measure** | **Steepness measure** | **Likelihood ratio test** | |
| --- | --- | --- | --- |
|  |  | ***χ*^2^** | **p** |
| *NDS_Dij_* | *NDS_Dij_* | 4.5311 | 0.3389 |
| *NDS_Dij_*-based ordinal rank |  | 3.9740 | 0.4095 |
| *Elo*_rpt_ | *Elo*_rpt_ | 8.8933 | 0.0638 |
| *Elo*_rpt_-based ordinal rank |  | 8.9606 | 0.0621 |
| *Elo*_Bayes_ | *Elo*_Bayes_ | 7.9872 | 0.0920 |
| *Elo*_Bayes_-based ordinal rank |  | 7.0646 | 0.1325 |
| *DS*_Bayes_ | *DS*_Bayes_ | 5.4146 | 0.2473 |
| *DS*_Bayes_-based ordinal rank |  | 4.5545 | 0.3361 |

**Table S20** Complete dataset, model statistics for the full meta-regression model showing the effect of the study setting on the relationship between *NDS_Dij_*-based steepness on the distribution of benefits in relation to dominance rank when the effect size is calculated using individual *NDS_Dij_* (n = 153). The interaction between the two variables is represented by the multiplication sign (×) between the variable names. The phylogenetic signal (λ) of the model is less than 0.001, which is not statistically different from zero (p = 0.999).

| **Moderator** | **Estimate** | **SE** | ***Z*** | **p** | **Lower 95% CI** | **Upper 95% CI** |
| --- | --- | --- | --- | --- | --- | --- |
| intercept | 0.4511 | 0.0665 | 6.7849 | <.0001*** | 0.3208 | 0.5814 |
| *NDS_Dij_*-based steepness | 0.0107 | 0.0406 | 0.2646 | 0.7913 | -0.0688 | 0.0902 |
| Study duration | -0.03 | 0.0386 | -0.7777 | 0.4367 | -0.1056 | 0.0456 |
| Benefit category_direct | 0.1096 | 0.0951 | 1.1516 | 0.2495 | -0.0769 | 0.296 |
| Dispersal pattern_FP | -0.1916 | 0.238 | -0.8052 | 0.4207 | -0.6581 | 0.2748 |
| Dispersal pattern_MFP | 0.109 | 0.2915 | 0.3739 | 0.7085 | -0.4624 | 0.6804 |
| Social organization_MLS | 0.424 | 0.4374 | 0.9694 | 0.3323 | -0.4333 | 1.2813 |
| Social organization_MMG | 0.2152 | 0.2246 | 0.9579 | 0.3381 | -0.2251 | 0.6554 |
| Study setting_captive | 0.3063 | 0.3975 | 0.7706 | 0.441 | -0.4728 | 1.0853 |
| Study setting_provisioned | 0.0648 | 0.1579 | 0.4107 | 0.6813 | -0.2446 | 0.3743 |
| Sex category_female | -0.2847 | 0.06 | -4.7454 | <.0001*** | -0.4023 | -0.1671 |
| Sex category_mixed | -0.0751 | 0.1474 | -0.5095 | 0.6104 | -0.364 | 0.2138 |
| Data origin_published | 0.4609 | 0.182 | 2.5323 | 0.0113* | 0.1042 | 0.8177 |
| *NDS_Dij_*-based steepness × Study setting_provisioned | -0.0831 | 0.0799 | -1.0407 | 0.298 | -0.2397 | 0.0734 |

**Table S21** Complete dataset: model statistics for the full meta-regression model showing the effect of the study setting on the relationship between *NDS_Dij_*-based steepness on the distribution of benefits in relation to dominance rank when the effect size is calculated using individual *NDS_Dij_*-based ordinal ranks (n = 153). The interaction between the two variables is represented by the multiplication sign (×) between the variable names. The phylogenetic signal (λ) of the model is less than 0.001, which is not statistically different from zero (p = 0.999).

| **Moderator** | **Estimate** | **SE** | ***Z*** | **p** | **Lower 95% CI** | **Upper 95% CI** |
| --- | --- | --- | --- | --- | --- | --- |
| intercept | 0.4357 | 0.07 | 6.2272 | <.0001*** | 0.2985 | 0.5728 |
| *NDS_Dij_*-based steepness | 0.0049 | 0.0405 | 0.1214 | 0.9034 | -0.0745 | 0.0843 |
| Study duration | -0.0282 | 0.038 | -0.7429 | 0.4575 | -0.1026 | 0.0462 |
| Benefit category_direct | 0.0878 | 0.094 | 0.9338 | 0.3504 | -0.0965 | 0.2721 |
| Dispersal pattern_FP | -0.1826 | 0.2416 | -0.7557 | 0.4498 | -0.656 | 0.2909 |
| Dispersal pattern_MFP | 0.0478 | 0.2995 | 0.1596 | 0.8732 | -0.5392 | 0.6348 |
| Social organization_MLS | 0.5423 | 0.4428 | 1.2246 | 0.2207 | -0.3256 | 1.4102 |
| Social organization_MMG | 0.1736 | 0.2257 | 0.7691 | 0.4418 | -0.2688 | 0.6161 |
| Study setting_captive | 0.3114 | 0.3972 | 0.7839 | 0.4331 | -0.4671 | 1.0899 |
| Study setting_provisioned | 0.0918 | 0.149 | 0.6165 | 0.5375 | -0.2001 | 0.3838 |
| Sex category_female | -0.3291 | 0.0598 | -5.5054 | <.0001*** | -0.4462 | -0.2119 |
| Sex category_mixed | -0.1406 | 0.146 | -0.9629 | 0.3356 | -0.4267 | 0.1456 |
| Data origin_published | 0.4881 | 0.1624 | 3.0064 | 0.0026** | 0.1699 | 0.8063 |
| *NDS_Dij_*-based steepness × Study setting_provisioned | -0.1092 | 0.0798 | -1.3688 | 0.1711 | -0.2656 | 0.0472 |

**Table S22** Complete dataset: model statistics for the full meta-regression model showing the effect of the study setting on the relationship between *Elo*_rpt_-based steepness on the distribution of benefits in relation to dominance rank when the effect size is calculated using individual *Elo*_rpt_ (n = 153). The interaction between the two variables is represented by the multiplication sign (×) between the variable names. The phylogenetic signal (λ) of the model is less than 0.001, which is not statistically different from zero (p = 0.999).

| **Moderator** | **Estimate** | **SE** | ***Z*** | **p** | **Lower 95% CI** | **Upper 95% CI** |
| --- | --- | --- | --- | --- | --- | --- |
| Intercept | 0.4478 | 0.0651 | 6.8729 | <.0001*** | 0.3201 | 0.5754 |
| *Elo*_rpt_-based steepness | 0.0709 | 0.0393 | 1.8027 | 0.0714. | -0.0062 | 0.1479 |
| Study duration | -0.0114 | 0.0355 | -0.3221 | 0.7474 | -0.0809 | 0.0581 |
| Benefit category_direct | 0.1098 | 0.0913 | 1.2029 | 0.229 | -0.0691 | 0.2888 |
| Dispersal pattern_FP | -0.1846 | 0.2361 | -0.7816 | 0.4344 | -0.6474 | 0.2782 |
| Dispersal pattern_MFP | -0.053 | 0.2874 | -0.1843 | 0.8538 | -0.6163 | 0.5104 |
| Social organization_MLS | 0.6191 | 0.4362 | 1.4193 | 0.1558 | -0.2358 | 1.4739 |
| Social organization_MMG | 0.2088 | 0.2213 | 0.9436 | 0.3454 | -0.2249 | 0.6426 |
| Study setting_captive | 0.4986 | 0.3889 | 1.282 | 0.1998 | -0.2637 | 1.261 |
| Study setting_provisioned | 0.1285 | 0.1561 | 0.8235 | 0.4102 | -0.1774 | 0.4344 |
| Sex category_female | -0.3106 | 0.058 | -5.3532 | <.0001*** | -0.4244 | -0.1969 |
| Sex category_mixed | -0.0952 | 0.1452 | -0.6558 | 0.5119 | -0.3798 | 0.1893 |
| Data origin_published | 0.4935 | 0.1771 | 2.7872 | 0.0053** | 0.1465 | 0.8406 |
| *Elo*_rpt_-based steepness × Study setting_provisioned | -0.0699 | 0.0803 | -0.871 | 0.3838 | -0.2273 | 0.0874 |

**Table S23** Complete dataset: model statistics for the full meta-regression model showing the effect of the study setting on the relationship between *Elo*_rpt_-based steepness on the distribution of benefits in relation to dominance rank when the effect size is calculated using individual *Elo*_rpt_-based ordinal ranks (n = 153). The interaction between the two variables is represented by the multiplication sign (×) between the variable names. The phylogenetic signal (λ) of the model is less than 0.001, which is not statistically different from zero (p = 0.999).

| **Moderator** | **Estimate** | **SE** | ***Z*** | **p** | **Lower 95% CI** | **Upper 95% CI** |
| --- | --- | --- | --- | --- | --- | --- |
| Intercept | 0.4548 | 0.0617 | 7.3665 | <.0001*** | 0.3338 | 0.5758 |
| *Elo*_rpt_-based steepness | 0.0611 | 0.039 | 1.5651 | 0.1176 | -0.0154 | 0.1376 |
| Study duration | -0.0162 | 0.0352 | -0.4614 | 0.6445 | -0.0853 | 0.0528 |
| Benefit category_direct | 0.1223 | 0.0907 | 1.3485 | 0.1775 | -0.0555 | 0.3001 |
| Dispersal pattern_FP | -0.2119 | 0.2284 | -0.9276 | 0.3536 | -0.6595 | 0.2358 |
| Dispersal pattern_MFP | -0.038 | 0.2792 | -0.1359 | 0.8919 | -0.5852 | 0.5093 |
| Social organization_MLS | 0.5832 | 0.4278 | 1.3632 | 0.1728 | -0.2553 | 1.4218 |
| Social organization_MMG | 0.2164 | 0.2176 | 0.9945 | 0.32 | -0.2101 | 0.6428 |
| Study setting_captive | 0.4145 | 0.3851 | 1.0765 | 0.2817 | -0.3402 | 1.1692 |
| Study setting_provisioned | 0.1038 | 0.151 | 0.6871 | 0.492 | -0.1922 | 0.3997 |
| Sex category_female | -0.3152 | 0.0579 | -5.4404 | <.0001*** | -0.4288 | -0.2016 |
| Sex category_mixed | -0.1121 | 0.1447 | -0.7752 | 0.4382 | -0.3957 | 0.1714 |
| Data origin_published | 0.4726 | 0.1704 | 2.7731 | 0.0056** | 0.1386 | 0.8067 |
| *Elo*_rpt_-based steepness × Study setting_provisioned | -0.0729 | 0.0797 | -0.9141 | 0.3606 | -0.2291 | 0.0834 |

**Table S24** Complete dataset: model statistics for the full meta-regression model showing the effect of the study setting on the relationship between *Elo*_Bayes_-based steepness on the distribution of benefits in relation to dominance rank when the effect size is calculated using individual *Elo*_Bayes_ (n = 153). The interaction between the two variables is represented by the multiplication sign (×) between the variable names. The phylogenetic signal (λ) of the model is less than 0.001, which is not statistically different from zero (p = 0.999).

| **Moderator** | **Estimate** | **SE** | ***Z*** | **p** | **Lower 95% CI** | **Upper 95% CI** |
| --- | --- | --- | --- | --- | --- | --- |
| intercept | 0.4591 | 0.0612 | 7.5079 | <.0001*** | 0.3393 | 0.579 |
| *Elo*_Bayes_-based steepness | 0.0506 | 0.0408 | 1.2406 | 0.2148 | -0.0293 | 0.1305 |
| Study duration | -0.0229 | 0.0356 | -0.6444 | 0.5193 | -0.0927 | 0.0468 |
| Benefit category_direct | 0.1027 | 0.0918 | 1.1185 | 0.2633 | -0.0773 | 0.2827 |
| Dispersal pattern_FP | -0.2579 | 0.2264 | -1.1389 | 0.2548 | -0.7016 | 0.1859 |
| Dispersal pattern_MFP | 0.0915 | 0.2818 | 0.3247 | 0.7454 | -0.4608 | 0.6438 |
| Social organization_MLS | 0.3734 | 0.4271 | 0.8741 | 0.3821 | -0.4638 | 1.2105 |
| Social organization_MMG | 0.2267 | 0.2176 | 1.0421 | 0.2974 | -0.1997 | 0.6532 |
| Study setting_captive | 0.4251 | 0.384 | 1.1073 | 0.2682 | -0.3274 | 1.1777 |
| Study setting_provisioned | 0.0669 | 0.1502 | 0.4456 | 0.6559 | -0.2274 | 0.3612 |
| Sex category_female | -0.2971 | 0.0583 | -5.0986 | <.0001*** | -0.4113 | -0.1829 |
| Sex category_mixed | -0.1147 | 0.1457 | -0.7873 | 0.4311 | -0.4003 | 0.1708 |
| Data origin_published | 0.4454 | 0.1693 | 2.6304 | 0.0085** | 0.1135 | 0.7772 |
| *Elo*_Bayes_-based steepness × Study setting_provisioned | -0.0668 | 0.0818 | -0.8166 | 0.4141 | -0.2271 | 0.0935 |

**Table S25** Complete dataset: model statistics for the full meta-regression model showing the effect of the study setting on the relationship between *Elo*_Bayes_-based steepness on the distribution of benefits in relation to dominance rank when the effect size is calculated using individual *Elo*_Bayes_-based ordinal ranks (n = 153). The interaction between the two variables is represented by the multiplication sign (×) between the variable names. The phylogenetic signal (λ) of the model is less than 0.001, which is not statistically different from zero (p = 0.999).

| **Moderator** | **Estimate** | **SE** | ***Z*** | **p** | **Lower 95% CI** | **Upper 95% CI** |
| --- | --- | --- | --- | --- | --- | --- |
| intercept | 0.4513 | 0.0605 | 7.4599 | <.0001*** | 0.3327 | 0.5699 |
| *Elo*_Bayes_-based steepness | 0.0523 | 0.0407 | 1.2861 | 0.1984 | -0.0274 | 0.1321 |
| Study duration | -0.0252 | 0.0356 | -0.7086 | 0.4786 | -0.0949 | 0.0445 |
| Benefit category_direct | 0.1201 | 0.0918 | 1.3087 | 0.1906 | -0.0598 | 0.3001 |
| Dispersal pattern_FP | -0.2413 | 0.225 | -1.0725 | 0.2835 | -0.6822 | 0.1997 |
| Dispersal pattern_MFP | 0.041 | 0.2803 | 0.1462 | 0.8838 | -0.5083 | 0.5903 |
| Social organization_MLS | 0.4675 | 0.4257 | 1.0981 | 0.2722 | -0.3669 | 1.3019 |
| Social organization_MMG | 0.2066 | 0.2169 | 0.9525 | 0.3408 | -0.2186 | 0.6318 |
| Study setting_captive | 0.3381 | 0.3833 | 0.8821 | 0.3777 | -0.4131 | 1.0893 |
| Study setting_provisioned | 0.0943 | 0.1492 | 0.6318 | 0.5275 | -0.1982 | 0.3867 |
| Sex category_female | -0.308 | 0.0583 | -5.2874 | <.0001*** | -0.4222 | -0.1938 |
| Sex category_mixed | -0.1073 | 0.1456 | -0.7372 | 0.461 | -0.3927 | 0.178 |
| Data origin_published | 0.4567 | 0.1681 | 2.7174 | 0.0066** | 0.1273 | 0.7861 |
| *Elo*_Bayes_-based steepness × Study setting_provisioned | -0.0851 | 0.0817 | -1.0421 | 0.2973 | -0.2452 | 0.075 |

**Table S26** Complete dataset: model statistics for the full meta-regression model showing the effect of the study setting on the relationship between *DS*_Bayes_-based steepness on the distribution of benefits in relation to dominance rank when the effect size is calculated using individual *DS*_Bayes_ (n = 153). The interaction between the two variables is represented by the multiplication sign (×) between the variable names. The phylogenetic signal (λ) of the model is less than 0.001, which is not statistically different from zero (p = 0.999).

| **Moderator** | **Estimate** | **SE** | ***Z*** | **p** | **Lower 95% CI** | **Upper 95% CI** |
| --- | --- | --- | --- | --- | --- | --- |
| intercept | 0.441 | 0.0656 | 6.7228 | <.0001*** | 0.3124 | 0.5696 |
| *DS*_Bayes_-based steepness | 0.0219 | 0.0428 | 0.5114 | 0.609 | -0.062 | 0.1058 |
| Study duration | -0.0285 | 0.039 | -0.7301 | 0.4653 | -0.105 | 0.048 |
| Benefit category_direct | 0.0581 | 0.0975 | 0.5959 | 0.5512 | -0.1331 | 0.2493 |
| Dispersal pattern_FP | -0.203 | 0.2377 | -0.8537 | 0.3932 | -0.6689 | 0.263 |
| Dispersal pattern_MFP | 0.148 | 0.2901 | 0.5102 | 0.6099 | -0.4205 | 0.7165 |
| Social organization_MLS | 0.4178 | 0.4371 | 0.9558 | 0.3392 | -0.4389 | 1.2745 |
| Social organization_MMG | 0.2442 | 0.2249 | 1.0856 | 0.2777 | -0.1967 | 0.6851 |
| Study setting_captive | 0.2793 | 0.3956 | 0.706 | 0.4802 | -0.4961 | 1.0546 |
| Study setting_provisioned | 0.0664 | 0.1579 | 0.4209 | 0.6739 | -0.243 | 0.3759 |
| Sex category_female | -0.2632 | 0.0598 | -4.4027 | <.0001*** | -0.3803 | -0.146 |
| Sex category_mixed | -0.1034 | 0.1503 | -0.6876 | 0.4917 | -0.398 | 0.1913 |
| Data origin_published | 0.4447 | 0.1807 | 2.4614 | 0.0138* | 0.0906 | 0.7989 |
| *DS*_Bayes_-based steepness × Study setting_provisioned | -0.1099 | 0.0853 | -1.2882 | 0.1977 | -0.2771 | 0.0573 |

**Table S27** Complete dataset: model statistics for the full meta-regression model showing the effect of the study setting on the relationship between *DS*_Bayes_-based steepness on the distribution of benefits in relation to dominance rank when the effect size is calculated using individual *DS*_Bayes_-based ordinal ranks (n = 153). The interaction between the two variables is represented by the multiplication sign (×) between the variable names. The phylogenetic signal (λ) of the model is less than 0.001, which is not statistically different from zero (p = 0.999).

| **Moderator** | **Estimate** | **SE** | ***Z*** | **p** | **Lower 95% CI** | **Upper 95% CI** |
| --- | --- | --- | --- | --- | --- | --- |
| intercept | 0.4189 | 0.0665 | 6.2984 | <.0001*** | 0.2886 | 0.5493 |
| *DS*_Bayes_-based steepness | 0.0167 | 0.0427 | 0.3905 | 0.6961 | -0.067 | 0.1003 |
| Study duration | -0.0321 | 0.0387 | -0.8304 | 0.4063 | -0.1079 | 0.0437 |
| Benefit category_direct | 0.0695 | 0.097 | 0.7166 | 0.4736 | -0.1206 | 0.2596 |
| Dispersal pattern_FP | -0.2211 | 0.2394 | -0.9234 | 0.3558 | -0.6904 | 0.2482 |
| Dispersal pattern_MFP | 0.0249 | 0.2919 | 0.0851 | 0.9322 | -0.5473 | 0.597 |
| Social organization_MLS | 0.5555 | 0.4377 | 1.2692 | 0.2044 | -0.3024 | 1.4134 |
| Social organization_MMG | 0.1661 | 0.2251 | 0.7378 | 0.4606 | -0.2751 | 0.6073 |
| Study setting_captive | 0.2948 | 0.3955 | 0.7454 | 0.4561 | -0.4803 | 1.0699 |
| Study setting_provisioned | 0.0827 | 0.1586 | 0.5216 | 0.602 | -0.2282 | 0.3937 |
| Sex category_female | -0.291 | 0.0597 | -4.8755 | <.0001*** | -0.408 | -0.174 |
| Sex category_mixed | -0.1364 | 0.1502 | -0.9077 | 0.364 | -0.4308 | 0.1581 |
| Data origin_published | 0.4709 | 0.1818 | 2.5908 | 0.0096** | 0.1147 | 0.8272 |
| *DS*_Bayes_-based steepness × Study setting_provisioned | -0.1225 | 0.0852 | -1.4381 | 0.1504 | -0.2894 | 0.0445 |

**Table S28** Test statistics of the likelihood ratio tests comparing each full model, in which the interaction effect between steepness and the study setting is included, with the respective control model. The degrees of freedom of each full model and the respective model were 17 and 13, respectively.

| **Dominance rank measure** | **Steepness measure** | **Likelihood ratio test** | |
| --- | --- | --- | --- |
|  |  | ***χ*^2^** | **p** |
| *NDS_Dij_* | *NDS_Dij_* | 3.9485 | 0.4130 |
| *NDS_Dij_*-based ordinal rank |  | 4.8686 | 0.3011 |
| *Elo*_rpt_ | *Elo*_rpt_ | 7.3017 | 0.1208 |
| *Elo*_rpt_-based ordinal rank |  | 6.0140 | 0.1981 |
| *Elo*_Bayes_ | *Elo*_Bayes_ | 5.0427 | 0.2829 |
| *Elo*_Bayes_-based ordinal rank |  | 5.0648 | 0.2807 |
| *DS*_Bayes_ | *DS*_Bayes_ | 4.2613 | 0.3718 |
| *DS*_Bayes_-based ordinal rank |  | 4.7665 | 0.3121 |

**Table S29** Complete dataset: model statistics for the full meta-regression model showing the effect of the sex category of the study group on the relationship between *NDS_Dij_*-based steepness on the distribution of benefits in relation to dominance rank when the effect size is calculated using individual *NDS_Dij_* (n = 153). The interaction between the two variables is represented by the multiplication sign (×) between the variable names. The phylogenetic signal (λ) of the model is less than 0.001, which is not statistically different from zero (p = 0.999).

| **Moderator** | **Estimate** | **SE** | ***Z*** | **p** | **Lower 95% CI** | **Upper 95% CI** |
| --- | --- | --- | --- | --- | --- | --- |
| intercept | 0.4593 | 0.0637 | 7.214 | <.0001*** | 0.3345 | 0.5841 |
| *NDS_Dij_*-based steepness | 0.0008 | 0.039 | 0.0209 | 0.9834 | -0.0756 | 0.0772 |
| Study duration | -0.0205 | 0.0401 | -0.511 | 0.6094 | -0.0991 | 0.0581 |
| Benefit category_direct | 0.122 | 0.0946 | 1.2905 | 0.1969 | -0.0633 | 0.3074 |
| Dispersal pattern_FP | -0.2041 | 0.2324 | -0.8781 | 0.3799 | -0.6596 | 0.2514 |
| Dispersal pattern_MFP | 0.0866 | 0.2843 | 0.3045 | 0.7608 | -0.4707 | 0.6438 |
| Social organization_MLS | 0.4711 | 0.4302 | 1.0951 | 0.2735 | -0.372 | 1.3142 |
| Social organization_MMG | 0.2318 | 0.222 | 1.0443 | 0.2963 | -0.2032 | 0.6668 |
| Study setting_captive | 0.351 | 0.3924 | 0.8944 | 0.3711 | -0.4182 | 1.1201 |
| Study setting_provisioned | 0.0482 | 0.1555 | 0.3096 | 0.7568 | -0.2567 | 0.353 |
| Sex category_female | -0.2892 | 0.0654 | -4.4203 | <.0001*** | -0.4174 | -0.1609 |
| Sex category_mixed | -0.0519 | 0.1489 | -0.3487 | 0.7273 | -0.3437 | 0.2398 |
| Data origin_published | 0.4287 | 0.1779 | 2.41 | 0.016* | 0.08 | 0.7773 |
| *NDS_Dij_*-based steepness × Sex category_female | -0.0158 | 0.0589 | -0.2684 | 0.7884 | -0.1312 | 0.0996 |

**Table S30** Complete dataset: model statistics for the full meta-regression model showing the effect of the sex category of the study group on the relationship between *NDS_Dij_*-based steepness on the distribution of benefits in relation to dominance rank when the effect size is calculated using individual *NDS_Dij_*-based ordinal ranks (n = 153). The interaction between the two variables is represented by the multiplication sign (×) between the variable names. The phylogenetic signal (λ) of the model is less than 0.001, which is not statistically different from zero (p = 0.999).

| **Moderator** | **Estimate** | **SE** | ***Z*** | **p** | **Lower 95% CI** | **Upper 95% CI** |
| --- | --- | --- | --- | --- | --- | --- |
| intercept | 0.4465 | 0.0671 | 6.6549 | <.0001*** | 0.315 | 0.578 |
| *NDS_Dij_*-based steepness | -0.0085 | 0.0387 | -0.2193 | 0.8264 | -0.0843 | 0.0674 |
| Study duration | -0.0188 | 0.0393 | -0.4799 | 0.6313 | -0.0958 | 0.0581 |
| Benefit category_direct | 0.1072 | 0.093 | 1.1521 | 0.2493 | -0.0752 | 0.2896 |
| Dispersal pattern_FP | -0.2032 | 0.2357 | -0.8619 | 0.3887 | -0.6652 | 0.2588 |
| Dispersal pattern_MFP | 0.0104 | 0.292 | 0.0356 | 0.9716 | -0.5619 | 0.5827 |
| Social organization_MLS | 0.597 | 0.4348 | 1.3731 | 0.1697 | -0.2552 | 1.4491 |
| Social organization_MMG | 0.195 | 0.2229 | 0.875 | 0.3816 | -0.2418 | 0.6318 |
| Study setting_captive | 0.3774 | 0.392 | 0.9628 | 0.3357 | -0.3909 | 1.1458 |
| Study setting_provisioned | 0.0757 | 0.1468 | 0.5155 | 0.6062 | -0.2121 | 0.3634 |
| Sex category_female | -0.329 | 0.0651 | -5.0569 | <.0001*** | -0.4566 | -0.2015 |
| Sex category_mixed | -0.1168 | 0.1472 | -0.7937 | 0.4273 | -0.4054 | 0.1717 |
| Data origin_published | 0.4528 | 0.1592 | 2.8437 | 0.0045** | 0.1407 | 0.7649 |
| *NDS_Dij_*-based steepness ×Sex category_female | -0.0076 | 0.0586 | -0.1303 | 0.8964 | -0.1224 | 0.1071 |

**Table S31** Complete dataset: model statistics for the full meta-regression model showing the effect of the sex category of the study group on the relationship between *Elo*_rpt_-based steepness on the distribution of benefits in relation to dominance rank when the effect size is calculated using individual *Elo*_rpt_ (n = 153). The interaction between the two variables is represented by the multiplication sign (×) between the variable names. The phylogenetic signal (λ) of the model is less than 0.001, which is not statistically different from zero (p = 0.999).

| **Moderator** | **Estimate** | **SE** | ***Z*** | **p** | **Lower 95% CI** | **Upper 95% CI** |
| --- | --- | --- | --- | --- | --- | --- |
| Intercept | 0.4519 | 0.0652 | 6.927 | <.0001*** | 0.324 | 0.5798 |
| *Elo*_rpt_-based steepness | 0.0631 | 0.039 | 1.619 | 0.1055 | -0.0133 | 0.1394 |
| Study duration | -0.0126 | 0.0357 | -0.3522 | 0.7247 | -0.0825 | 0.0574 |
| Benefit category_direct | 0.1194 | 0.0912 | 1.31 | 0.1902 | -0.0593 | 0.2981 |
| Dispersal pattern_FP | -0.2001 | 0.2358 | -0.8485 | 0.3961 | -0.6624 | 0.2621 |
| Dispersal pattern_MFP | -0.0659 | 0.2873 | -0.2295 | 0.8185 | -0.6291 | 0.4972 |
| Social organization_MLS | 0.6698 | 0.4367 | 1.534 | 0.125 | -0.186 | 1.5256 |
| Social organization_MMG | 0.2085 | 0.2216 | 0.9409 | 0.3467 | -0.2258 | 0.6427 |
| Study setting_captive | 0.4778 | 0.3885 | 1.2297 | 0.2188 | -0.2837 | 1.2392 |
| Study setting_provisioned | 0.1209 | 0.1561 | 0.774 | 0.4389 | -0.1852 | 0.4269 |
| Sex category_female | -0.3094 | 0.058 | -5.333 | <.0001*** | -0.4232 | -0.1957 |
| Sex category_mixed | -0.0936 | 0.1453 | -0.6441 | 0.5195 | -0.3783 | 0.1911 |
| Data origin_published | 0.4835 | 0.1769 | 2.7333 | 0.0063** | 0.1368 | 0.8302 |
| *Elo*_rpt_-based steepness ×Sex category_female | -0.017 | 0.0713 | -0.2384 | 0.8116 | -0.1568 | 0.1228 |

**Table S32** Complete dataset: model statistics for the full meta-regression model showing the effect of the sex category of the study group on the relationship between *Elo*_rpt_-based steepness on the distribution of benefits in relation to dominance rank when the effect size is calculated using individual *Elo*_rpt_-based ordinal ranks (n = 153). The interaction between the two variables is represented by the multiplication sign (×) between the variable names. The phylogenetic signal (λ) of the model is less than 0.001, which is not statistically different from zero (p = 0.999).

| **Moderator** | **Estimate** | **SE** | ***Z*** | **p** | **Lower 95% CI** | **Upper 95% CI** |
| --- | --- | --- | --- | --- | --- | --- |
| Intercept | 0.4583 | 0.0615 | 7.4554 | <.0001*** | 0.3378 | 0.5788 |
| *Elo*_rpt_-based steepness | 0.0545 | 0.0387 | 1.4088 | 0.1589 | -0.0213 | 0.1304 |
| Study duration | -0.0183 | 0.0354 | -0.517 | 0.6052 | -0.0878 | 0.0511 |
| Benefit category_direct | 0.1351 | 0.0905 | 1.4932 | 0.1354 | -0.0422 | 0.3124 |
| Dispersal pattern_FP | -0.2322 | 0.2273 | -1.0216 | 0.307 | -0.6778 | 0.2133 |
| Dispersal pattern_MFP | -0.0538 | 0.2783 | -0.1932 | 0.8468 | -0.5993 | 0.4918 |
| Social organization_MLS | 0.6257 | 0.4274 | 1.4639 | 0.1432 | -0.212 | 1.4634 |
| Social organization_MMG | 0.2163 | 0.2174 | 0.9948 | 0.3198 | -0.2098 | 0.6424 |
| Study setting_captive | 0.3929 | 0.3842 | 1.0226 | 0.3065 | -0.3602 | 1.146 |
| Study setting_provisioned | 0.0957 | 0.1505 | 0.6358 | 0.5249 | -0.1993 | 0.3907 |
| Sex category_female | -0.3136 | 0.0579 | -5.4144 | <.0001*** | -0.4271 | -0.2001 |
| Sex category_mixed | -0.1099 | 0.1447 | -0.7599 | 0.4473 | -0.3935 | 0.1736 |
| Data origin_published | 0.4611 | 0.1695 | 2.7197 | 0.0065** | 0.1288 | 0.7934 |
| *Elo*_rpt_-based steepness ×Sex category_female | -0.0003 | 0.0712 | -0.0042 | 0.9967 | -0.1398 | 0.1392 |

**Table S33** Complete dataset, model statistics for the full meta-regression model showing the effect of the sex category of the study group on the relationship between *Elo*_Bayes_-based steepness on the distribution of benefits in relation to dominance rank when the effect size is calculated using individual *Elo*_Bayes_ (n = 153). The interaction between the two variables is represented by the multiplication sign (×) between the variable names. The phylogenetic signal (λ) of the model is less than 0.001, which is not statistically different from zero (p = 0.999).

| **Moderator** | **Estimate** | **SE** | ***Z*** | **p** | **Lower 95% CI** | **Upper 95% CI** |
| --- | --- | --- | --- | --- | --- | --- |
| intercept | 0.4614 | 0.0615 | 7.4967 | <.0001*** | 0.3407 | 0.582 |
| *Elo*_Bayes_-based steepness | 0.0378 | 0.0382 | 0.9883 | 0.323 | -0.0371 | 0.1127 |
| Study duration | -0.0214 | 0.0362 | -0.5913 | 0.5543 | -0.0923 | 0.0495 |
| Benefit category_direct | 0.1074 | 0.0927 | 1.1585 | 0.2466 | -0.0743 | 0.2892 |
| Dispersal pattern_FP | -0.2619 | 0.2272 | -1.1528 | 0.249 | -0.7072 | 0.1834 |
| Dispersal pattern_MFP | 0.0612 | 0.2796 | 0.2188 | 0.8268 | -0.4868 | 0.6091 |
| Social organization_MLS | 0.4436 | 0.4271 | 1.0388 | 0.2989 | -0.3934 | 1.2806 |
| Social organization_MMG | 0.2357 | 0.2185 | 1.0788 | 0.2807 | -0.1925 | 0.6639 |
| Study setting_captive | 0.4228 | 0.3849 | 1.0985 | 0.272 | -0.3315 | 1.1771 |
| Study setting_provisioned | 0.0603 | 0.1509 | 0.3995 | 0.6895 | -0.2354 | 0.3559 |
| Sex category_female | -0.2931 | 0.0589 | -4.9795 | <.0001*** | -0.4084 | -0.1777 |
| Sex category_mixed | -0.1152 | 0.1459 | -0.7895 | 0.4298 | -0.4012 | 0.1708 |
| Data origin_published | 0.4395 | 0.1701 | 2.5838 | 0.0098** | 0.1061 | 0.7728 |
| *Elo*_Bayes_-based steepness ×Sex category_female | -0.0289 | 0.0672 | -0.4302 | 0.667 | -0.1607 | 0.1028 |

**Table S34** Complete dataset: model statistics for the full meta-regression model showing the effect of the sex category of the study group on the relationship between *Elo*_Bayes_-based steepness on the distribution of benefits in relation to dominance rank when the effect size is calculated using individual *Elo*_Bayes_-based ordinal ranks (n = 153). The interaction between the two variables is represented by the multiplication sign (×) between the variable names. The phylogenetic signal (λ) of the model is less than 0.001, which is not statistically different from zero (p = 0.999).

| **Moderator** | **Estimate** | **SE** | ***Z*** | **p** | **Lower 95% CI** | **Upper 95% CI** |
| --- | --- | --- | --- | --- | --- | --- |
| intercept | 0.4531 | 0.0612 | 7.4007 | <.0001*** | 0.3331 | 0.5731 |
| *Elo*_Bayes_-based steepness | 0.0363 | 0.0382 | 0.9501 | 0.342 | -0.0386 | 0.1112 |
| Study duration | -0.0243 | 0.0362 | -0.672 | 0.5016 | -0.0953 | 0.0466 |
| Benefit category_direct | 0.1293 | 0.0928 | 1.3933 | 0.1635 | -0.0526 | 0.3111 |
| Dispersal pattern_FP | -0.2496 | 0.2265 | -1.1017 | 0.2706 | -0.6936 | 0.1944 |
| Dispersal pattern_MFP | -0.0011 | 0.2788 | -0.0041 | 0.9967 | -0.5477 | 0.5454 |
| Social organization_MLS | 0.5468 | 0.4265 | 1.2822 | 0.1998 | -0.289 | 1.3827 |
| Social organization_MMG | 0.2156 | 0.2182 | 0.9882 | 0.3231 | -0.2121 | 0.6433 |
| Study setting_captive | 0.3371 | 0.3846 | 0.8765 | 0.3807 | -0.4167 | 1.0909 |
| Study setting_provisioned | 0.0876 | 0.1504 | 0.5822 | 0.5604 | -0.2073 | 0.3824 |
| Sex category_female | -0.3045 | 0.0589 | -5.1745 | <.0001*** | -0.4199 | -0.1892 |
| Sex category_mixed | -0.1082 | 0.1459 | -0.7416 | 0.4584 | -0.3942 | 0.1778 |
| Data origin_published | 0.4507 | 0.1695 | 2.6587 | 0.0078** | 0.1185 | 0.783 |
| *Elo*_Bayes_-based steepness ×Sex category_female | -0.0232 | 0.0672 | -0.3449 | 0.7302 | -0.1549 | 0.1086 |

**Table S35** Complete dataset, model statistics for the full meta-regression model showing the effect of the sex category of the study group on the relationship between *DS*_Bayes_-based steepness on the distribution of benefits in relation to dominance rank when the effect size is calculated using individual *DS*_Bayes_ (n = 153). The interaction between the two variables is represented by the multiplication sign (×) between the variable names. The phylogenetic signal (λ) of the model is less than 0.001, which is not statistically different from zero (p = 0.999).

| **Moderator** | **Estimate** | **SE** | ***Z*** | **p** | **Lower 95% CI** | **Upper 95% CI** |
| --- | --- | --- | --- | --- | --- | --- |
| intercept | 0.4525 | 0.0614 | 7.366 | <.0001*** | 0.3321 | 0.5729 |
| *DS*_Bayes_-based steepness | 0.001 | 0.0386 | 0.0271 | 0.9784 | -0.0746 | 0.0767 |
| Study duration | -0.0143 | 0.0397 | -0.3593 | 0.7194 | -0.0921 | 0.0636 |
| Benefit category_direct | 0.0725 | 0.0965 | 0.7509 | 0.4527 | -0.1167 | 0.2617 |
| Dispersal pattern_FP | -0.2381 | 0.2278 | -1.0451 | 0.296 | -0.6847 | 0.2085 |
| Dispersal pattern_MFP | 0.1198 | 0.2796 | 0.4283 | 0.6684 | -0.4283 | 0.6678 |
| Social organization_MLS | 0.4733 | 0.4265 | 1.1098 | 0.2671 | -0.3626 | 1.3092 |
| Social organization_MMG | 0.2681 | 0.2206 | 1.2153 | 0.2243 | -0.1643 | 0.7005 |
| Study setting_captive | 0.3156 | 0.3903 | 0.8085 | 0.4188 | -0.4494 | 1.0806 |
| Study setting_provisioned | 0.0367 | 0.1523 | 0.2413 | 0.8093 | -0.2617 | 0.3352 |
| Sex category_female | -0.275 | 0.0657 | -4.1878 | <.0001*** | -0.4037 | -0.1463 |
| Sex category_mixed | -0.0651 | 0.1529 | -0.4258 | 0.6702 | -0.3649 | 0.2346 |
| Data origin_published | 0.4001 | 0.1731 | 2.3113 | 0.0208* | 0.0608 | 0.7395 |
| *DS*_Bayes_-based steepness × Sex category_female | -0.0207 | 0.0555 | -0.3727 | 0.7094 | -0.1294 | 0.0881 |

**Table S36** Complete dataset: model statistics for the full meta-regression model showing the effect of the sex category of the study group on the relationship between *DS*_Bayes_-based steepness on the distribution of benefits in relation to dominance rank when the effect size is calculated using individual *DS*_Bayes_-based ordinal ranks (n = 153). The interaction between the two variables is represented by the multiplication sign (×) between the variable names. The phylogenetic signal (λ) of the model is less than 0.001, which is not statistically different from zero (p = 0.999).

| **Moderator** | **Estimate** | **SE** | ***Z*** | **p** | **Lower 95% CI** | **Upper 95% CI** |
| --- | --- | --- | --- | --- | --- | --- |
| intercept | 0.4319 | 0.0627 | 6.8934 | <.0001*** | 0.3091 | 0.5548 |
| *DS*_Bayes_-based steepness | -0.0063 | 0.0382 | -0.1656 | 0.8685 | -0.0813 | 0.0686 |
| Study duration | -0.0205 | 0.0391 | -0.5245 | 0.5999 | -0.0971 | 0.0561 |
| Benefit category_direct | 0.0892 | 0.0956 | 0.9333 | 0.3507 | -0.0981 | 0.2765 |
| Dispersal pattern_FP | -0.2675 | 0.23 | -1.163 | 0.2448 | -0.7184 | 0.1833 |
| Dispersal pattern_MFP | -0.0194 | 0.282 | -0.0688 | 0.9451 | -0.5722 | 0.5334 |
| Social organization_MLS | 0.6121 | 0.4269 | 1.4339 | 0.1516 | -0.2246 | 1.4488 |
| Social organization_MMG | 0.1947 | 0.2207 | 0.8824 | 0.3776 | -0.2378 | 0.6273 |
| Study setting_captive | 0.3493 | 0.3901 | 0.8955 | 0.3705 | -0.4152 | 1.1138 |
| Study setting_provisioned | 0.0562 | 0.1533 | 0.3667 | 0.7138 | -0.2442 | 0.3566 |
| Sex category_female | -0.2946 | 0.0654 | -4.5039 | <.0001*** | -0.4229 | -0.1664 |
| Sex category_mixed | -0.1071 | 0.1528 | -0.7014 | 0.483 | -0.4065 | 0.1922 |
| Data origin_published | 0.4287 | 0.1747 | 2.4546 | 0.0141* | 0.0864 | 0.7711 |
| *DS*_Bayes_-based steepness × Sex category_female | -0.0042 | 0.0553 | -0.0756 | 0.9398 | -0.1125 | 0.1041 |

**Table S37** Test statistics of the likelihood ratio tests comparing each full model, in which the interaction effect between steepness and the sex category of the study group is included, with the respective control model. The degrees of freedom of each full model and the respective model were 17 and 13, respectively.

| **Dominance rank measure** | **Steepness measure** | **Likelihood ratio test** | |
| --- | --- | --- | --- |
|  |  | ***χ*^2^** | **p** |
| *NDS_Dij_* | *NDS_Dij_* | 22.8726 | 0.0001 |
| *NDS_Dij_*-based ordinal rank |  | 29.6922 | <.0001 |
| *Elo*_rpt_ | *Elo*_rpt_ | 29.6024 | <.0001 |
| *Elo*_rpt_-based ordinal rank |  | 29.5877 | <.0001 |
| *Elo*_Bayes_ | *Elo*_Bayes_ | 25.2812 | <.0001 |
| *Elo*_Bayes_-based ordinal rank |  | 27.1719 | <.0001 |
| *DS*_Bayes_ | *DS*_Bayes_ | 19.5720 | 0.0006 |
| *DS*_Bayes_-based ordinal rank |  | 23.3121 | 0.0001 |

**Table S38** Restricted dataset including only groups in which the dominance hierarchy is significantly linear: model statistics for the full meta-regression model showing the effect of *NDS_Dij_*-based steepness on the distribution of benefits in relation to dominance rank when the effect size is calculated using individual *NDS_Dij_* (n = 99). The phylogenetic signal (λ) of the model is less than 0.001, which is not statistically different from zero (p = 0.999).

| **Moderator** | **Estimate** | **SE** | ***Z*** | **p** | **Lower 95% CI** | **Upper 95% CI** |
| --- | --- | --- | --- | --- | --- | --- |
| intercept | 0.3787 | 0.058 | 6.5302 | <.0001*** | 0.2651 | 0.4924 |
| *NDS_Dij_*-based steepness | 0.0123 | 0.0436 | 0.282 | 0.778 | -0.0731 | 0.0977 |
| Study duration | -0.0328 | 0.0491 | -0.6678 | 0.5043 | -0.129 | 0.0634 |
| Benefit category_direct | 0.1467 | 0.1076 | 1.3629 | 0.1729 | -0.0643 | 0.3577 |
| Dispersal pattern_FP | -0.1542 | 0.2227 | -0.6923 | 0.4887 | -0.5906 | 0.2823 |
| Dispersal pattern_MFP | -0.0321 | 0.3753 | -0.0855 | 0.9319 | -0.7677 | 0.7036 |
| Social organization_MLS | 0.3531 | 0.4705 | 0.7505 | 0.4529 | -0.5691 | 1.2754 |
| Social organization_MMG | -0.0222 | 0.2958 | -0.0751 | 0.9402 | -0.602 | 0.5576 |
| Study setting_captive | 1.0419 | 0.5204 | 2.0022 | 0.0453* | 0.022 | 2.0619 |
| Study setting_provisioned | 0.0981 | 0.1506 | 0.6515 | 0.5147 | -0.1971 | 0.3933 |
| Sex category_female | -0.2635 | 0.0685 | -3.8459 | 0.0001*** | -0.3977 | -0.1292 |
| Sex category_mixed | 0.0021 | 0.158 | 0.0132 | 0.9895 | -0.3076 | 0.3117 |
| Data origin_published | 0.4117 | 0.1726 | 2.3851 | 0.0171* | 0.0734 | 0.7501 |

**Table S39** Restricted dataset including only groups in which the dominance hierarchy is significantly linear: model statistics for the full meta-regression model showing the effect of *NDS_Dij_*-based steepness on the distribution of benefits in relation to dominance rank when the effect size is calculated using individual *NDS_Dij_*-based ordinal ranks (n = 99). The phylogenetic signal (λ) of the model is less than 0.001, which is not statistically different from zero (p = 0.999).

| **Moderator** | **Estimate** | **SE** | ***Z*** | **p** | **Lower 95% CI** | **Upper 95% CI** |
| --- | --- | --- | --- | --- | --- | --- |
| intercept | 0.3681 | 0.0642 | 5.7363 | <.0001*** | 0.2424 | 0.4939 |
| *NDS_Dij_*-based steepness | -0.0044 | 0.043 | -0.1018 | 0.9189 | -0.0886 | 0.0799 |
| Study duration | -0.0275 | 0.0473 | -0.5826 | 0.5601 | -0.1202 | 0.0651 |
| Benefit category_direct | 0.1397 | 0.1054 | 1.3257 | 0.1849 | -0.0668 | 0.3463 |
| Dispersal pattern_FP | -0.1569 | 0.2276 | -0.6893 | 0.4906 | -0.603 | 0.2892 |
| Dispersal pattern_MFP | -0.1295 | 0.3823 | -0.3388 | 0.7348 | -0.8789 | 0.6198 |
| Social organization_MLS | 0.4736 | 0.4737 | 0.9997 | 0.3174 | -0.4549 | 1.4021 |
| Social organization_MMG | -0.0796 | 0.2954 | -0.2695 | 0.7876 | -0.6586 | 0.4994 |
| Study setting_captive | 0.9514 | 0.5202 | 1.8288 | 0.0674. | -0.0682 | 1.9711 |
| Study setting_provisioned | 0.1191 | 0.146 | 0.8158 | 0.4146 | -0.1671 | 0.4054 |
| Sex category_female | -0.2969 | 0.0677 | -4.3819 | <.0001*** | -0.4296 | -0.1641 |
| Sex category_mixed | -0.067 | 0.1568 | -0.4273 | 0.6692 | -0.3744 | 0.2404 |
| Data origin_published | 0.4564 | 0.1587 | 2.8763 | 0.004** | 0.1454 | 0.7675 |

**Table S40** Restricted dataset including only groups in which the dominance hierarchy is significantly linear: model statistics for the full meta-regression model showing the effect of *Elo*_rpt_-based steepness on the distribution of benefits in relation to dominance rank when the effect size is calculated using individual *Elo*_rpt_ (n = 99). The phylogenetic signal (λ) of the model is less than 0.001, which is not statistically different from zero (p = 0.999).

| **Moderator** | **Estimate** | **SE** | ***Z*** | **p** | **Lower 95% CI** | **Upper 95% CI** |
| --- | --- | --- | --- | --- | --- | --- |
| Intercept | 0.3761 | 0.0593 | 6.3385 | <.0001*** | 0.2598 | 0.4924 |
| *Elo*_rpt_-based steepness | 0.0481 | 0.0415 | 1.1578 | 0.2469 | -0.0333 | 0.1294 |
| Study duration | -0.0199 | 0.0451 | -0.4418 | 0.6586 | -0.1082 | 0.0684 |
| Benefit category_direct | 0.1442 | 0.103 | 1.4 | 0.1615 | -0.0577 | 0.346 |
| Dispersal pattern_FP | -0.2026 | 0.2233 | -0.9074 | 0.3642 | -0.6403 | 0.2351 |
| Dispersal pattern_MFP | -0.1806 | 0.3748 | -0.482 | 0.6298 | -0.9153 | 0.554 |
| Social organization_MLS | 0.5383 | 0.4756 | 1.132 | 0.2577 | -0.3938 | 1.4704 |
| Social organization_MMG | -0.0773 | 0.2958 | -0.2613 | 0.7939 | -0.657 | 0.5024 |
| Study setting_captive | 1.0622 | 0.5121 | 2.0742 | 0.0381* | 0.0585 | 2.0658 |
| Study setting_provisioned | 0.1148 | 0.1521 | 0.7551 | 0.4502 | -0.1832 | 0.4129 |
| Sex category_female | -0.281 | 0.0654 | -4.297 | <.0001*** | -0.4091 | -0.1528 |
| Sex category_mixed | -0.0655 | 0.1549 | -0.4227 | 0.6725 | -0.369 | 0.238 |
| Data origin_published | 0.4083 | 0.1729 | 2.3607 | 0.0182* | 0.0693 | 0.7472 |

**Table S41** Restricted dataset including only groups in which the dominance hierarchy is significantly linear: model statistics for the full meta-regression model showing the effect of *Elo*_rpt_-based steepness on the distribution of benefits in relation to dominance rank when the effect size is calculated using individual *Elo*_rpt_-based ordinal ranks (n = 99). The phylogenetic signal (λ) of the model is less than 0.001, which is not statistically different from zero (p = 0.999).

| **Moderator** | **Estimate** | **SE** | ***Z*** | **p** | **Lower 95% CI** | **Upper 95% CI** |
| --- | --- | --- | --- | --- | --- | --- |
| Intercept | 0.3783 | 0.0572 | 6.6192 | <.0001*** | 0.2663 | 0.4903 |
| *Elo*_rpt_-based steepness | 0.037 | 0.0415 | 0.8916 | 0.3726 | -0.0443 | 0.1182 |
| Study duration | -0.0224 | 0.0451 | -0.496 | 0.6199 | -0.1108 | 0.066 |
| Benefit category_direct | 0.1461 | 0.1028 | 1.4214 | 0.1552 | -0.0553 | 0.3474 |
| Dispersal pattern_FP | -0.2338 | 0.2193 | -1.0662 | 0.2863 | -0.6636 | 0.196 |
| Dispersal pattern_MFP | -0.1726 | 0.3712 | -0.4649 | 0.642 | -0.9002 | 0.555 |
| Social organization_MLS | 0.4804 | 0.4725 | 1.0166 | 0.3093 | -0.4457 | 1.4065 |
| Social organization_MMG | -0.0531 | 0.2943 | -0.1804 | 0.8569 | -0.63 | 0.5238 |
| Study setting_captive | 0.8808 | 0.5097 | 1.728 | 0.084. | -0.1182 | 1.8798 |
| Study setting_provisioned | 0.1124 | 0.1485 | 0.7568 | 0.4492 | -0.1787 | 0.4036 |
| Sex category_female | -0.2861 | 0.0654 | -4.3768 | <.0001*** | -0.4142 | -0.158 |
| Sex category_mixed | -0.0603 | 0.1545 | -0.3902 | 0.6964 | -0.3631 | 0.2426 |
| Data origin_published | 0.4112 | 0.1692 | 2.4303 | 0.0151* | 0.0796 | 0.7428 |

**Table S42** Restricted dataset including only groups in which the dominance hierarchy is significantly linear: model statistics for the full meta-regression model showing the effect of *Elo*_Bayes_-based steepness on the distribution of benefits in relation to dominance rank when the effect size is calculated using individual *Elo*_Bayes_ (n = 99). The phylogenetic signal (λ) of the model is less than 0.001, which is not statistically different from zero (p = 0.999).

| **Moderator** | **Estimate** | **SE** | ***Z*** | **p** | **Lower 95% CI** | **Upper 95% CI** |
| --- | --- | --- | --- | --- | --- | --- |
| intercept | 0.3764 | 0.0579 | 6.5 | <.0001*** | 0.2629 | 0.4899 |
| *Elo*_Bayes_-based steepness | 0.035 | 0.0383 | 0.9138 | 0.3608 | -0.0401 | 0.1101 |
| Study duration | -0.0292 | 0.0458 | -0.6374 | 0.5238 | -0.119 | 0.0606 |
| Benefit category_direct | 0.1451 | 0.1049 | 1.3827 | 0.1668 | -0.0606 | 0.3508 |
| Dispersal pattern_FP | -0.2388 | 0.222 | -1.0758 | 0.282 | -0.674 | 0.1963 |
| Dispersal pattern_MFP | -0.0675 | 0.3734 | -0.1807 | 0.8566 | -0.7993 | 0.6643 |
| Social organization_MLS | 0.3494 | 0.4712 | 0.7415 | 0.4584 | -0.5742 | 1.273 |
| Social organization_MMG | -0.0056 | 0.2954 | -0.0189 | 0.9849 | -0.5846 | 0.5734 |
| Study setting_captive | 1.1046 | 0.512 | 2.1575 | 0.031* | 0.1011 | 2.1081 |
| Study setting_provisioned | 0.1064 | 0.1502 | 0.708 | 0.4789 | -0.1881 | 0.4008 |
| Sex category_female | -0.2661 | 0.0655 | -4.061 | <.0001*** | -0.3945 | -0.1377 |
| Sex category_mixed | -0.0632 | 0.1553 | -0.4067 | 0.6842 | -0.3675 | 0.2412 |
| Data origin_published | 0.412 | 0.171 | 2.4093 | 0.016* | 0.0768 | 0.7471 |

**Table S43** Restricted dataset including only groups in which the dominance hierarchy is significantly linear: model statistics for the full meta-regression model showing the effect of *Elo*_Bayes_-based steepness on the distribution of benefits in relation to dominance rank when the effect size is calculated using individual *Elo*_Bayes_-based ordinal ranks (n = 99). The phylogenetic signal (λ) of the model is less than 0.001, which is not statistically different from zero (p = 0.999).

| **Moderator** | **Estimate** | **SE** | ***Z*** | **p** | **Lower 95% CI** | **Upper 95% CI** |
| --- | --- | --- | --- | --- | --- | --- |
| intercept | 0.3711 | 0.0569 | 6.5241 | <.0001*** | 0.2596 | 0.4826 |
| *Elo*_Bayes_-based steepness | 0.0307 | 0.0382 | 0.8032 | 0.4219 | -0.0441 | 0.1054 |
| Study duration | -0.0302 | 0.0455 | -0.6625 | 0.5076 | -0.1194 | 0.0591 |
| Benefit category_direct | 0.1549 | 0.1042 | 1.487 | 0.137 | -0.0493 | 0.359 |
| Dispersal pattern_FP | -0.2517 | 0.2198 | -1.1451 | 0.2522 | -0.6825 | 0.1791 |
| Dispersal pattern_MFP | -0.1629 | 0.3709 | -0.4393 | 0.6604 | -0.8899 | 0.564 |
| Social organization_MLS | 0.4477 | 0.4684 | 0.9557 | 0.3392 | -0.4704 | 1.3658 |
| Social organization_MMG | -0.0652 | 0.2939 | -0.2219 | 0.8244 | -0.6413 | 0.5109 |
| Study setting_captive | 0.8629 | 0.5097 | 1.6929 | 0.0905. | -0.1361 | 1.8619 |
| Study setting_provisioned | 0.0975 | 0.1482 | 0.6579 | 0.5106 | -0.193 | 0.388 |
| Sex category_female | -0.2757 | 0.0654 | -4.2134 | <.0001*** | -0.4039 | -0.1474 |
| Sex category_mixed | -0.0704 | 0.1549 | -0.4544 | 0.6496 | -0.374 | 0.2332 |
| Data origin_published | 0.4035 | 0.1688 | 2.3907 | 0.0168* | 0.0727 | 0.7343 |

**Table S44** Restricted dataset including only groups in which the dominance hierarchy is significantly linear: model statistics for the full meta-regression model showing the effect of *DS*_Bayes_-based steepness on the distribution of benefits in relation to dominance rank when the effect size is calculated using individual *DS*_Bayes_ (n = 99). The phylogenetic signal (λ) of the model is less than 0.001, which is not statistically different from zero (p = 0.999).

| **Moderator** | **Estimate** | **SE** | ***Z*** | **p** | **Lower 95% CI** | **Upper 95% CI** |
| --- | --- | --- | --- | --- | --- | --- |
| intercept | 0.3727 | 0.0594 | 6.2768 | <.0001*** | 0.2563 | 0.4891 |
| *DS*_Bayes_-based steepness | 0.009 | 0.0439 | 0.2048 | 0.8377 | -0.0771 | 0.0951 |
| Study duration | -0.0307 | 0.0484 | -0.6346 | 0.5257 | -0.1257 | 0.0642 |
| Benefit category_direct | 0.1297 | 0.1092 | 1.1874 | 0.2351 | -0.0844 | 0.3437 |
| Dispersal pattern_FP | -0.1815 | 0.2241 | -0.8097 | 0.4181 | -0.6207 | 0.2578 |
| Dispersal pattern_MFP | -0.0352 | 0.3773 | -0.0933 | 0.9256 | -0.7747 | 0.7043 |
| Social organization_MLS | 0.3568 | 0.4735 | 0.7536 | 0.4511 | -0.5712 | 1.2849 |
| Social organization_MMG | -0.0018 | 0.2972 | -0.006 | 0.9952 | -0.5843 | 0.5807 |
| Study setting_captive | 1.0144 | 0.522 | 1.9435 | 0.052. | -0.0086 | 2.0374 |
| Study setting_provisioned | 0.1069 | 0.1532 | 0.6979 | 0.4852 | -0.1934 | 0.4073 |
| Sex category_female | -0.2464 | 0.0676 | -3.6439 | 0.0003*** | -0.3789 | -0.1139 |
| Sex category_mixed | -0.0143 | 0.1609 | -0.0888 | 0.9292 | -0.3297 | 0.3011 |
| Data origin_published | 0.4157 | 0.1749 | 2.3762 | 0.0175* | 0.0728 | 0.7585 |

**Table S45** Restricted dataset including only groups in which the dominance hierarchy is significantly linear: model statistics for the full meta-regression model showing the effect of *DS*_Bayes_-based steepness on the distribution of benefits in relation to dominance rank when the effect size is calculated using individual *DS*_Bayes_-based ordinal ranks (n = 99). The phylogenetic signal (λ) of the model is less than 0.001, which is not statistically different from zero (p = 0.999).

| **Moderator** | **Estimate** | **SE** | ***Z*** | **p** | **Lower 95% CI** | **Upper 95% CI** |
| --- | --- | --- | --- | --- | --- | --- |
| intercept | 0.3585 | 0.0622 | 5.7655 | <.0001*** | 0.2366 | 0.4804 |
| *DS*_Bayes_-based steepness | -0.0061 | 0.0431 | -0.1405 | 0.8883 | -0.0906 | 0.0785 |
| Study duration | -0.0302 | 0.0468 | -0.6448 | 0.5191 | -0.122 | 0.0616 |
| Benefit category_direct | 0.1253 | 0.1071 | 1.1705 | 0.2418 | -0.0845 | 0.3352 |
| Dispersal pattern_FP | -0.219 | 0.228 | -0.9607 | 0.3367 | -0.6659 | 0.2278 |
| Dispersal pattern_MFP | -0.1463 | 0.3788 | -0.3862 | 0.6993 | -0.8888 | 0.5962 |
| Social organization_MLS | 0.4796 | 0.472 | 1.0162 | 0.3096 | -0.4455 | 1.4047 |
| Social organization_MMG | -0.0856 | 0.296 | -0.2892 | 0.7724 | -0.6656 | 0.4945 |
| Study setting_captive | 0.9234 | 0.5199 | 1.7762 | 0.0757. | -0.0955 | 1.9423 |
| Study setting_provisioned | 0.0851 | 0.1563 | 0.5445 | 0.5861 | -0.2212 | 0.3914 |
| Sex category_female | -0.2708 | 0.0671 | -4.0364 | <.0001*** | -0.4023 | -0.1393 |
| Sex category_mixed | -0.0693 | 0.1605 | -0.4318 | 0.6659 | -0.3838 | 0.2452 |
| Data origin_published | 0.4258 | 0.178 | 2.3922 | 0.0167* | 0.0769 | 0.7747 |

**Table S46** Complete dataset: model statistics for the simpler meta-regression models in which only the predictor (i.e., steepness) is included, and statistics of the likelihood ratio test comparing the full model with the respective control model (n = 153). The degrees of freedom of each full model and the respective model were 5 and 4, respectively.

| **Dominance rank measure** | **Predictor** | **Estimate** | **SE** | ***Z*** | **p** | **Lower 95% CI** | **Upper 95% CI** | **Likelihood ratio test** | |
| --- | --- | --- | --- | --- | --- | --- | --- | --- | --- |
|  |  |  |  |  |  |  |  | ***χ*^2^** | **p** |
| *NDS_Dij_* | *NDS_Dij_*-based steepness | 0.0384 | 0.0356 | 1.0798 | 0.2802 | -0.0313 | 0.1082 | 1.2047 | 0.2724 |
| *NDS_Dij_*-based ordinal rank |  | 0.0482 | 0.0356 | 1.3551 | 0.1754 | -0.0215 | 0.118 | 1.9046 | 0.1676 |
| *Elo*_rpt_ | *Elo*_rpt_-based steepness | 0.0317 | 0.0382 | 0.8304 | 0.4063 | -0.0431 | 0.1065 | 0.9953 | 0.3185 |
| *Elo*_rpt_-based ordinal rank |  | 0.022 | 0.0379 | 0.5807 | 0.5615 | -0.0522 | 0.0962 | 0.6348 | 0.4256 |
| *Elo*_Bayes_ | *Elo*_Bayes_-based steepness | -0.0083 | 0.0375 | -0.2218 | 0.8245 | -0.0818 | 0.0651 | 0.3281 | 0.5668 |
| *Elo*_Bayes_-based ordinal rank |  | -0.0121 | 0.0374 | -0.3247 | 0.7454 | -0.0854 | 0.0611 | 0.3759 | 0.5398 |
| *DS*_Bayes_ | *DS*_Bayes_-based steepness | 0.0318 | 0.0348 | 0.914 | 0.3607 | -0.0364 | 0.1001 | 0.8710 | 0.3507 |
| *DS*_Bayes_-based ordinal rank |  | 0.0281 | 0.0341 | 0.8249 | 0.4094 | -0.0387 | 0.0949 | 0.6927 | 0.4052 |

**Table S47** Restricted dataset including only groups in which the dominance hierarchy is significantly linear: model statistics for the simpler meta-regression models in which only the predictor (i.e., steepness) is included, and statistics of the likelihood ratio test comparing the full model with the respective control model (n = 99). The degrees of freedom of each full model and the respective model were 5 and 4, respectively.

| **Dominance rank measure** | **Predictor** | **Estimate** | **SE** | ***Z*** | **p** | **Lower 95% CI** | **Upper 95% CI** | **Likelihood ratio test** | |
| --- | --- | --- | --- | --- | --- | --- | --- | --- | --- |
|  |  |  |  |  |  |  |  | ***χ*^2^** | **p** |
| *NDS_Dij_* | *NDS_Dij_*-based steepness | 0.0715 | 0.0367 | 1.9482 | 0.0514. | -0.0004 | 0.1435 | 2.7237 | 0.0989. |
| *NDS_Dij_*-based ordinal rank |  | 0.0694 | 0.0364 | 1.9063 | 0.0566. | -0.002 | 0.1407 | 2.9000 | 0.0886. |
| *Elo*_rpt_ | *Elo*_rpt_-based steepness | 0.0316 | 0.0395 | 0.8013 | 0.423 | -0.0458 | 0.109 | 0.5485 | 0.4589 |
| *Elo*_rpt_-based ordinal rank |  | 0.0211 | 0.0393 | 0.5367 | 0.5915 | -0.056 | 0.0981 | 0.2063 | 0.6497 |
| *Elo*_Bayes_ | *Elo*_Bayes_-based steepness | 0.0176 | 0.0362 | 0.4861 | 0.6269 | -0.0534 | 0.0887 | 0.0000 | 1.0000 |
| *Elo*_Bayes_-based ordinal rank |  | 0.0093 | 0.0359 | 0.2602 | 0.7947 | -0.061 | 0.0797 | 0.0000 | 1.0000 |
| *DS*_Bayes_ | *DS*_Bayes_-based steepness | 0.0562 | 0.0368 | 1.5297 | 0.1261 | -0.0158 | 0.1283 | 1.3888 | 0.2386 |
| *DS*_Bayes_-based ordinal rank |  | 0.0384 | 0.0353 | 1.0902 | 0.2756 | -0.0307 | 0.1076 | 0.4426 | 0.5059 |

**Table S48** Published dataset: model statistics for the full meta-regression model showing the effect of *NDS_Dij_*-based steepness on the distribution of benefits in relation to dominance rank when the effect size is calculated using individual *NDS_Dij_* (n = 75). The phylogenetic signal (λ) of the model is less than 0.001, which is not statistically different from zero (p = 0.999).

| **Moderator** | **Estimate** | **SE** | ***Z*** | **p** | **Lower 95% CI** | **Upper 95% CI** |
| --- | --- | --- | --- | --- | --- | --- |
| intercept | 0.7083 | 0.1016 | 6.9715 | <.0001*** | 0.5092 | 0.9074 |
| *NDS_Dij_*-based steepness | 0.0507 | 0.0821 | 0.6179 | 0.5367 | -0.1101 | 0.2115 |
| Study duration | 0.0114 | 0.1003 | 0.1135 | 0.9096 | -0.1852 | 0.208 |
| Benefit category_direct | 0.1353 | 0.1599 | 0.8465 | 0.3973 | -0.178 | 0.4486 |
| Dispersal pattern_FP | -0.0852 | 0.3088 | -0.276 | 0.7826 | -0.6904 | 0.5199 |
| Dispersal pattern_MFP | 0.1075 | 0.3667 | 0.2932 | 0.7694 | -0.6113 | 0.8263 |
| Social organization_MLS | 0.2468 | 0.5936 | 0.4158 | 0.6776 | -0.9167 | 1.4103 |
| Social organization_MMG | 0.2527 | 0.2871 | 0.8803 | 0.3787 | -0.31 | 0.8155 |
| Study setting_captive | 0.9735 | 0.565 | 1.723 | 0.0849. | -0.1339 | 2.0809 |
| Study setting_provisioned | 0.2615 | 0.2819 | 0.9274 | 0.3537 | -0.2911 | 0.814 |
| Sex category_female | -0.5327 | 0.1552 | -3.4331 | 0.0006*** | -0.8369 | -0.2286 |
| Sex category_mixed | -0.9543 | 0.4925 | -1.9376 | 0.0527. | -1.9197 | 0.011 |

**Table S49** Published dataset: model statistics for the full meta-regression model showing the effect of *NDS_Dij_*-based steepness on the distribution of benefits in relation to dominance rank when the effect size is calculated using individual *NDS_Dij_*-based ordinal ranks (n = 75). The phylogenetic signal (λ) of the model is less than 0.001, which is not statistically different from zero (p = 0.999).

| **Moderator** | **Estimate** | **SE** | ***Z*** | **p** | **Lower 95% CI** | **Upper 95% CI** |
| --- | --- | --- | --- | --- | --- | --- |
| intercept | 0.6866 | 0.0914 | 7.5111 | <.0001*** | 0.5074 | 0.8658 |
| *NDS_Dij_*-based steepness | 0.0579 | 0.0779 | 0.7439 | 0.4569 | -0.0947 | 0.2105 |
| Study duration | 0.0265 | 0.0926 | 0.2861 | 0.7748 | -0.1551 | 0.2081 |
| Benefit category_direct | 0.1071 | 0.1581 | 0.6775 | 0.4981 | -0.2027 | 0.417 |
| Dispersal pattern_FP | -0.1307 | 0.2765 | -0.4727 | 0.6364 | -0.6727 | 0.4113 |
| Dispersal pattern_MFP | 0.033 | 0.3371 | 0.098 | 0.9219 | -0.6276 | 0.6937 |
| Social organization_MLS | 0.5359 | 0.5398 | 0.9927 | 0.3209 | -0.5221 | 1.5938 |
| Social organization_MMG | 0.277 | 0.2687 | 1.0308 | 0.3026 | -0.2497 | 0.8037 |
| Study setting_captive | 0.9133 | 0.5246 | 1.7411 | 0.0817. | -0.1148 | 1.9415 |
| Study setting_provisioned | 0.2113 | 0.2172 | 0.9732 | 0.3304 | -0.2143 | 0.637 |
| Sex category_female | -0.4726 | 0.1488 | -3.1765 | 0.0015** | -0.7642 | -0.181 |
| Sex category_mixed | -0.8901 | 0.4541 | -1.9603 | 0.05* | -1.7801 | -0.0002 |

**Table S50** Published dataset: model statistics for the full meta-regression model showing the effect of *Elo*_rpt_-based steepness on the distribution of benefits in relation to dominance rank when the effect size is calculated using individual *Elo*_rpt_ (n = 75). The phylogenetic signal (λ) of the model is less than 0.001, which is not statistically different from zero (p = 0.999).

| **Moderator** | **Estimate** | **SE** | ***Z*** | **p** | **Lower 95% CI** | **Upper 95% CI** |
| --- | --- | --- | --- | --- | --- | --- |
| Intercept | 0.691 | 0.0977 | 7.07 | <.0001*** | 0.4994 | 0.8825 |
| *Elo*_rpt_-based steepness | 0.1426 | 0.0807 | 1.7663 | 0.0773. | -0.0156 | 0.3009 |
| Study duration | 0.0438 | 0.0967 | 0.4528 | 0.6507 | -0.1457 | 0.2332 |
| Benefit category_direct | 0.0891 | 0.1586 | 0.5619 | 0.5742 | -0.2217 | 0.3999 |
| Dispersal pattern_FP | -0.0374 | 0.2975 | -0.1256 | 0.9001 | -0.6205 | 0.5458 |
| Dispersal pattern_MFP | -0.0086 | 0.3558 | -0.0243 | 0.9806 | -0.706 | 0.6887 |
| Social organization_MLS | 0.5499 | 0.5767 | 0.9535 | 0.3403 | -0.5804 | 1.6801 |
| Social organization_MMG | 0.237 | 0.278 | 0.8527 | 0.3938 | -0.3078 | 0.7819 |
| Study setting_captive | 0.9768 | 0.5472 | 1.7853 | 0.0742. | -0.0956 | 2.0493 |
| Study setting_provisioned | 0.3464 | 0.2619 | 1.3226 | 0.186 | -0.1669 | 0.8596 |
| Sex category_female | -0.578 | 0.1446 | -3.9981 | <.0001*** | -0.8613 | -0.2946 |
| Sex category_mixed | -0.6911 | 0.48 | -1.4396 | 0.15 | -1.632 | 0.2498 |

**Table S51** Published dataset: model statistics for the full meta-regression model showing the effect of *Elo*_rpt_-based steepness on the distribution of benefits in relation to dominance rank when the effect size is calculated using individual *Elo*_rpt_-based ordinal ranks (n = 75). The phylogenetic signal (λ) of the model is less than 0.001, which is not statistically different from zero (p = 0.999).

| **Moderator** | **Estimate** | **SE** | ***Z*** | **p** | **Lower 95% CI** | **Upper 95% CI** |
| --- | --- | --- | --- | --- | --- | --- |
| Intercept | 0.6994 | 0.0952 | 7.346 | <.0001*** | 0.5128 | 0.886 |
| *Elo*_rpt_-based steepness | 0.1363 | 0.0796 | 1.7116 | 0.087. | -0.0198 | 0.2923 |
| Study duration | 0.0313 | 0.0947 | 0.3304 | 0.7411 | -0.1543 | 0.2168 |
| Benefit category_direct | 0.1085 | 0.1581 | 0.6866 | 0.4923 | -0.2013 | 0.4184 |
| Dispersal pattern_FP | -0.0639 | 0.2901 | -0.2203 | 0.8256 | -0.6325 | 0.5047 |
| Dispersal pattern_MFP | 0.0247 | 0.3482 | 0.071 | 0.9434 | -0.6577 | 0.7071 |
| Social organization_MLS | 0.4929 | 0.5644 | 0.8732 | 0.3825 | -0.6134 | 1.5991 |
| Social organization_MMG | 0.2699 | 0.2727 | 0.9899 | 0.3222 | -0.2645 | 0.8044 |
| Study setting_captive | 1.0327 | 0.5386 | 1.9173 | 0.0552. | -0.023 | 2.0884 |
| Study setting_provisioned | 0.3532 | 0.2546 | 1.3871 | 0.1654 | -0.1458 | 0.8521 |
| Sex category_female | -0.5747 | 0.1431 | -4.0167 | <.0001*** | -0.8552 | -0.2943 |
| Sex category_mixed | -0.8891 | 0.4721 | -1.8831 | 0.0597. | -1.8145 | 0.0363 |

**Table S52** Published dataset: model statistics for the full meta-regression model showing the effect of *Elo*_Bayes_-based steepness on the distribution of benefits in relation to dominance rank when the effect size is calculated using individual *Elo*_Bayes_ (n = 75). The phylogenetic signal (λ) of the model is less than 0.001, which is not statistically different from zero (p = 0.999).

| **Moderator** | **Estimate** | **SE** | ***Z*** | **p** | **Lower 95% CI** | **Upper 95% CI** |
| --- | --- | --- | --- | --- | --- | --- |
| intercept | 0.7089 | 0.1007 | 7.0372 | <.0001*** | 0.5114 | 0.9063 |
| *Elo*_Bayes_-based steepness | 0.1217 | 0.0841 | 1.4466 | 0.148 | -0.0432 | 0.2865 |
| Study duration | 0.018 | 0.0993 | 0.1809 | 0.8565 | -0.1766 | 0.2126 |
| Benefit category_direct | 0.0642 | 0.1589 | 0.404 | 0.6862 | -0.2473 | 0.3757 |
| Dispersal pattern_FP | -0.1052 | 0.3046 | -0.3454 | 0.7298 | -0.7022 | 0.4918 |
| Dispersal pattern_MFP | 0.1903 | 0.3696 | 0.5149 | 0.6066 | -0.5341 | 0.9148 |
| Social organization_MLS | 0.1586 | 0.5863 | 0.2705 | 0.7868 | -0.9905 | 1.3077 |
| Social organization_MMG | 0.2236 | 0.2853 | 0.7838 | 0.4332 | -0.3356 | 0.7828 |
| Study setting_captive | 1.0105 | 0.559 | 1.8076 | 0.0707. | -0.0851 | 2.1061 |
| Study setting_provisioned | 0.2736 | 0.2734 | 1.0008 | 0.3169 | -0.2622 | 0.8093 |
| Sex category_female | -0.6164 | 0.1467 | -4.2028 | <.0001*** | -0.9038 | -0.3289 |
| Sex category_mixed | -0.9336 | 0.4891 | -1.9089 | 0.0563. | -1.8921 | 0.025 |

**Table S53** Published dataset: model statistics for the full meta-regression model showing the effect of *Elo*_Bayes_-based steepness on the distribution of benefits in relation to dominance rank when the effect size is calculated using individual *Elo*_Bayes_-based ordinal ranks (n = 75). The phylogenetic signal (λ) of the model is less than 0.001, which is not statistically different from zero (p = 0.999).

| **Moderator** | **Estimate** | **SE** | ***Z*** | **p** | **Lower 95% CI** | **Upper 95% CI** |
| --- | --- | --- | --- | --- | --- | --- |
| intercept | 0.6902 | 0.0945 | 7.3029 | <.0001*** | 0.505 | 0.8754 |
| *Elo*_Bayes_-based steepness | 0.1233 | 0.0808 | 1.526 | 0.127 | -0.0351 | 0.2817 |
| Study duration | 0.0147 | 0.0941 | 0.1564 | 0.8757 | -0.1697 | 0.1991 |
| Benefit category_direct | 0.1152 | 0.1576 | 0.7308 | 0.4649 | -0.1937 | 0.424 |
| Dispersal pattern_FP | -0.1109 | 0.2863 | -0.3872 | 0.6986 | -0.672 | 0.4503 |
| Dispersal pattern_MFP | 0.0979 | 0.3504 | 0.2794 | 0.7799 | -0.5889 | 0.7847 |
| Social organization_MLS | 0.3416 | 0.5555 | 0.615 | 0.5386 | -0.7472 | 1.4304 |
| Social organization_MMG | 0.2374 | 0.2715 | 0.8744 | 0.3819 | -0.2947 | 0.7696 |
| Study setting_captive | 0.8745 | 0.5368 | 1.629 | 0.1033 | -0.1777 | 1.9267 |
| Study setting_provisioned | 0.3179 | 0.2544 | 1.2499 | 0.2113 | -0.1806 | 0.8165 |
| Sex category_female | -0.568 | 0.1428 | -3.9779 | <.0001*** | -0.8479 | -0.2882 |
| Sex category_mixed | -0.7912 | 0.4685 | -1.6886 | 0.0913. | -1.7095 | 0.1271 |

**Table S54** Published dataset: model statistics for the full meta-regression model showing the effect of *DS*_Bayes_-based steepness on the distribution of benefits in relation to dominance rank when the effect size is calculated using individual *DS*_Bayes_ (n = 75). The phylogenetic signal of the model is moderate (λ = 0.156), but not statistically different from zero (p = 0.870).

| **Moderator** | **Estimate** | **SE** | ***Z*** | **p** | **Lower 95% CI** | **Upper 95% CI** |
| --- | --- | --- | --- | --- | --- | --- |
| intercept | 0.7006 | 0.1434 | 4.8865 | <.0001*** | 0.4196 | 0.9815 |
| *DS*_Bayes_-based steepness | 0.0605 | 0.0861 | 0.7024 | 0.4824 | -0.1083 | 0.2293 |
| Study duration | 0.032 | 0.1035 | 0.309 | 0.7574 | -0.1708 | 0.2348 |
| Benefit category_direct | 0.0303 | 0.1608 | 0.1886 | 0.8504 | -0.2848 | 0.3455 |
| Dispersal pattern_FP | -0.0676 | 0.359 | -0.1882 | 0.8507 | -0.7712 | 0.636 |
| Dispersal pattern_MFP | 0.1001 | 0.4003 | 0.25 | 0.8026 | -0.6845 | 0.8846 |
| Social organization_MLS | 0.2628 | 0.6149 | 0.4273 | 0.6691 | -0.9424 | 1.4679 |
| Social organization_MMG | 0.3119 | 0.2934 | 1.063 | 0.2878 | -0.2631 | 0.8869 |
| Study setting_captive | 1.0857 | 0.5865 | 1.851 | 0.0642. | -0.0639 | 2.2353 |
| Study setting_provisioned | 0.3035 | 0.2881 | 1.0536 | 0.2921 | -0.2611 | 0.8681 |
| Sex category_female | -0.5651 | 0.1615 | -3.5003 | 0.0005*** | -0.8816 | -0.2487 |
| Sex category_mixed | -1.0942 | 0.507 | -2.1584 | 0.0309* | -2.0878 | -0.1006 |

**Table S55** Published dataset: model statistics for the full meta-regression model showing the effect of *DS*_Bayes_-based steepness on the distribution of benefits in relation to dominance rank when the effect size is calculated using individual *DS*_Bayes_-based ordinal ranks (n = 75). The phylogenetic signal (λ) of the model is less than 0.001, which is not statistically different from zero (p = 0.999).

| **Moderator** | **Estimate** | **SE** | ***Z*** | **p** | **Lower 95% CI** | **Upper 95% CI** |
| --- | --- | --- | --- | --- | --- | --- |
| intercept | 0.6864 | 0.0921 | 7.452 | <.0001*** | 0.5059 | 0.867 |
| *DS*_Bayes_-based steepness | 0.0756 | 0.0818 | 0.9241 | 0.3554 | -0.0848 | 0.2361 |
| Study duration | 0.0121 | 0.0949 | 0.1276 | 0.8984 | -0.1738 | 0.198 |
| Benefit category_direct | 0.0888 | 0.1588 | 0.559 | 0.5762 | -0.2225 | 0.4 |
| Dispersal pattern_FP | -0.1363 | 0.2785 | -0.4892 | 0.6247 | -0.6822 | 0.4097 |
| Dispersal pattern_MFP | 0.0273 | 0.3361 | 0.0811 | 0.9353 | -0.6314 | 0.686 |
| Social organization_MLS | 0.4879 | 0.5603 | 0.8709 | 0.3838 | -0.6102 | 1.5861 |
| Social organization_MMG | 0.2628 | 0.2725 | 0.9644 | 0.3348 | -0.2713 | 0.797 |
| Study setting_captive | 0.89 | 0.5402 | 1.6474 | 0.0995. | -0.1688 | 1.9488 |
| Study setting_provisioned | 0.2527 | 0.259 | 0.9755 | 0.3293 | -0.255 | 0.7603 |
| Sex category_female | -0.4593 | 0.1557 | -2.9491 | 0.0032** | -0.7646 | -0.1541 |
| Sex category_mixed | -0.8868 | 0.471 | -1.8828 | 0.0597. | -1.8099 | 0.0363 |

**Table S56** Unpublished dataset: model statistics for the full meta-regression model showing the effect of *NDS_Dij_*-based steepness on the distribution of benefits in relation to dominance rank when the effect size is calculated using individual *NDS_Dij_* (n = 78). The phylogenetic signal (λ) of the model is less than 0.001, which is not statistically different from zero (p = 0.999).

| **Moderator** | **Estimate** | **SE** | ***Z*** | **p** | **Lower 95% CI** | **Upper 95% CI** |
| --- | --- | --- | --- | --- | --- | --- |
| intercept | 0.2004 | 0.044 | 4.5538 | <.0001*** | 0.1141 | 0.2866 |
| *NDS_Dij_*-based steepness | -0.0244 | 0.0427 | -0.5716 | 0.5676 | -0.1081 | 0.0593 |
| Study duration | -0.0234 | 0.0396 | -0.5912 | 0.5544 | -0.1009 | 0.0542 |
| Benefit category_direct | 0.0023 | 0.1014 | 0.0231 | 0.9815 | -0.1963 | 0.201 |
| Study setting_provisioned | -0.2705 | 0.1366 | -1.9808 | 0.0476* | -0.5382 | -0.0028 |
| Sex category_female | -0.2505 | 0.0657 | -3.8139 | 0.0001*** | -0.3792 | -0.1218 |
| Sex category_mixed | 0.0958 | 0.1488 | 0.6441 | 0.5195 | -0.1957 | 0.3874 |

**Table S57** Unpublished dataset: model statistics for the full meta-regression model showing the effect of *NDS_Dij_*-based steepness on the distribution of benefits in relation to dominance rank when the effect size is calculated using individual *NDS_Dij_*-based ordinal ranks (n = 78). The phylogenetic signal (λ) of the model is less than 0.001, which is not statistically different from zero (p = 0.999).

| **Moderator** | **Estimate** | **SE** | ***Z*** | **p** | **Lower 95% CI** | **Upper 95% CI** |
| --- | --- | --- | --- | --- | --- | --- |
| intercept | 0.2063 | 0.0464 | 4.4467 | <.0001*** | 0.1154 | 0.2972 |
| *NDS_Dij_*-based steepness | -0.0293 | 0.0428 | -0.6844 | 0.4937 | -0.1133 | 0.0546 |
| Study duration | -0.0284 | 0.0393 | -0.7235 | 0.4694 | -0.1055 | 0.0486 |
| Benefit category_direct | 0.0164 | 0.1023 | 0.1605 | 0.8725 | -0.184 | 0.2169 |
| Study setting_provisioned | -0.2899 | 0.1415 | -2.0484 | 0.0405* | -0.5673 | -0.0125 |
| Sex category_female | -0.3057 | 0.0657 | -4.6539 | <.0001*** | -0.4344 | -0.1769 |
| Sex category_mixed | 0.0324 | 0.1493 | 0.2168 | 0.8283 | -0.2603 | 0.3251 |

**Table S58** Unpublished dataset: model statistics for the full meta-regression model showing the effect of *Elo*_rpt_-based steepness on the distribution of benefits in relation to dominance rank when the effect size is calculated using individual *Elo*_rpt_ (n = 78). The phylogenetic signal (λ) of the model is less than 0.001, which is not statistically different from zero (p = 0.999).

| **Moderator** | **Estimate** | **SE** | ***Z*** | **p** | **Lower 95% CI** | **Upper 95% CI** |
| --- | --- | --- | --- | --- | --- | --- |
| Intercept | 0.2164 | 0.0472 | 4.582 | <.0001*** | 0.1238 | 0.3089 |
| *Elo*_rpt_-based steepness | 0.0123 | 0.036 | 0.3417 | 0.7326 | -0.0582 | 0.0828 |
| Study duration | -0.0281 | 0.0364 | -0.7723 | 0.4399 | -0.0996 | 0.0433 |
| Benefit category_direct | 0.022 | 0.0995 | 0.2207 | 0.8253 | -0.1731 | 0.217 |
| Study setting_provisioned | -0.2418 | 0.14 | -1.7276 | 0.0841. | -0.5162 | 0.0325 |
| Sex category_female | -0.2725 | 0.0647 | -4.2093 | <.0001*** | -0.3994 | -0.1456 |
| Sex category_mixed | 0.0496 | 0.1466 | 0.3385 | 0.735 | -0.2377 | 0.3369 |

**Table S59** Unpublished dataset: model statistics for the full meta-regression model showing the effect of *Elo*_rpt_-based steepness on the distribution of benefits in relation to dominance rank when the effect size is calculated using individual *Elo*_rpt_-based ordinal ranks (n = 78). The phylogenetic signal (λ) of the model is less than 0.001, which is not statistically different from zero (p = 0.999).

| **Moderator** | **Estimate** | **SE** | ***Z*** | **p** | **Lower 95% CI** | **Upper 95% CI** |
| --- | --- | --- | --- | --- | --- | --- |
| Intercept | 0.2172 | 0.0415 | 5.2307 | <.0001*** | 0.1358 | 0.2986 |
| *Elo*_rpt_-based steepness | 0.0006 | 0.0357 | 0.0168 | 0.9866 | -0.0693 | 0.0705 |
| Study duration | -0.031 | 0.036 | -0.8616 | 0.3889 | -0.1016 | 0.0395 |
| Benefit category_direct | 0.0196 | 0.095 | 0.2062 | 0.8366 | -0.1667 | 0.2059 |
| Study setting_provisioned | -0.2509 | 0.1267 | -1.9803 | 0.0477* | -0.4991 | -0.0026 |
| Sex category_female | -0.2777 | 0.0647 | -4.2938 | <.0001*** | -0.4045 | -0.1509 |
| Sex category_mixed | 0.0593 | 0.1442 | 0.4113 | 0.6809 | -0.2233 | 0.3419 |

**Table S60** Unpublished dataset: model statistics for the full meta-regression model showing the effect of *Elo*_Bayes_-based steepness on the distribution of benefits in relation to dominance rank when the effect size is calculated using individual *Elo*_Bayes_ (n = 78). The phylogenetic signal (λ) of the model is less than 0.001, which is not statistically different from zero (p = 0.999).

| **Moderator** | **Estimate** | **SE** | ***Z*** | **p** | **Lower 95% CI** | **Upper 95% CI** |
| --- | --- | --- | --- | --- | --- | --- |
| intercept | 0.2056 | 0.0445 | 4.617 | <.0001*** | 0.1183 | 0.2929 |
| *Elo*_Bayes_-based steepness | -0.0128 | 0.0395 | -0.325 | 0.7452 | -0.0903 | 0.0646 |
| Study duration | -0.0286 | 0.037 | -0.7719 | 0.4402 | -0.1011 | 0.044 |
| Benefit category_direct | 0.0039 | 0.099 | 0.0392 | 0.9687 | -0.1902 | 0.1979 |
| Study setting_provisioned | -0.2608 | 0.1338 | -1.9491 | 0.0513. | -0.5231 | 0.0015 |
| Sex category_female | -0.2456 | 0.0652 | -3.7669 | 0.0002*** | -0.3734 | -0.1178 |
| Sex category_mixed | 0.0716 | 0.1466 | 0.4883 | 0.6253 | -0.2157 | 0.3589 |

**Table S61** Unpublished dataset: model statistics for the full meta-regression model showing the effect of *Elo*_Bayes_-based steepness on the distribution of benefits in relation to dominance rank when the effect size is calculated using individual *Elo*_Bayes_-based ordinal ranks (n = 78). The phylogenetic signal (λ) of the model is less than 0.001, which is not statistically different from zero (p = 0.999).

| **Moderator** | **Estimate** | **SE** | ***Z*** | **p** | **Lower 95% CI** | **Upper 95% CI** |
| --- | --- | --- | --- | --- | --- | --- |
| intercept | 0.211 | 0.0434 | 4.8608 | <.0001*** | 0.1259 | 0.2961 |
| *Elo*_Bayes_-based steepness | -0.0147 | 0.0397 | -0.3707 | 0.7109 | -0.0925 | 0.0631 |
| Study duration | -0.0329 | 0.0376 | -0.8759 | 0.3811 | -0.1065 | 0.0407 |
| Benefit category_direct | 0.0146 | 0.0992 | 0.1471 | 0.883 | -0.1799 | 0.2091 |
| Study setting_provisioned | -0.2746 | 0.132 | -2.0797 | 0.0375* | -0.5334 | -0.0158 |
| Sex category_female | -0.2635 | 0.0653 | -4.0378 | <.0001*** | -0.3914 | -0.1356 |
| Sex category_mixed | 0.0547 | 0.1468 | 0.3725 | 0.7095 | -0.2331 | 0.3424 |

**Table S62** Unpublished dataset: model statistics for the full meta-regression model showing the effect of *DS*_Bayes_-based steepness on the distribution of benefits in relation to dominance rank when the effect size is calculated using individual *DS*_Bayes_ (n = 78). The phylogenetic signal (λ) of the model is less than 0.001, which is not statistically different from zero (p = 0.999).

| **Moderator** | **Estimate** | **SE** | ***Z*** | **p** | **Lower 95% CI** | **Upper 95% CI** |
| --- | --- | --- | --- | --- | --- | --- |
| intercept | 0.19 | 0.0507 | 3.7454 | 0.0002*** | 0.0906 | 0.2894 |
| *DS*_Bayes_-based steepness | -0.0317 | 0.0437 | -0.725 | 0.4684 | -0.1173 | 0.0539 |
| Study duration | -0.019 | 0.0398 | -0.4786 | 0.6323 | -0.0969 | 0.0589 |
| Benefit category_direct | -0.0232 | 0.1098 | -0.2111 | 0.8328 | -0.2383 | 0.192 |
| Study setting_provisioned | -0.2785 | 0.1518 | -1.8352 | 0.0665. | -0.5759 | 0.0189 |
| Sex category_female | -0.223 | 0.0653 | -3.4134 | 0.0006*** | -0.3511 | -0.095 |
| Sex category_mixed | 0.0566 | 0.1549 | 0.3657 | 0.7146 | -0.2469 | 0.3601 |

**Table S63** Unpublished dataset: model statistics for the full meta-regression model showing the effect of *DS*_Bayes_-based steepness on the distribution of benefits in relation to dominance rank when the effect size is calculated using individual *DS*_Bayes_-based ordinal ranks (n = 78). The phylogenetic signal (λ) of the model is less than 0.001, which is not statistically different from zero (p = 0.999).

| **Moderator** | **Estimate** | **SE** | ***Z*** | **p** | **Lower 95% CI** | **Upper 95% CI** |
| --- | --- | --- | --- | --- | --- | --- |
| intercept | 0.184 | 0.0535 | 3.4368 | 0.0006*** | 0.0791 | 0.2889 |
| *DS*_Bayes_-based steepness | -0.0374 | 0.0436 | -0.8581 | 0.3908 | -0.1228 | 0.048 |
| Study duration | -0.0251 | 0.0392 | -0.6396 | 0.5224 | -0.102 | 0.0518 |
| Benefit category_direct | -0.0074 | 0.1099 | -0.0673 | 0.9464 | -0.2229 | 0.2081 |
| Study setting_provisioned | -0.2799 | 0.1574 | -1.7786 | 0.0753. | -0.5884 | 0.0285 |
| Sex category_female | -0.2664 | 0.0653 | -4.0807 | <.0001*** | -0.3944 | -0.1385 |
| Sex category_mixed | 0.0092 | 0.1552 | 0.0593 | 0.9527 | -0.295 | 0.3134 |

**Appendix 1** The published studies included in our dataset and the corresponding measures of fitness-related benefits used for the meta-regression analysis.

| **Species** | **Study setting** | **Sex category** | **Fitness-related benefit** | **Number of effect sizes obtained** | **Number of matrices available** | **Reference** |
| --- | --- | --- | --- | --- | --- | --- |
| *Macaca sylvanus* | provisioned | M | Mating success | 1 | 0 ^a^ | (Berghänel et al. 2010) |
| *Rhinopithecus roxellana* | provisioned | F | Mating success | 1 | 1 | (He et al. 2013) |
| *Alouatta palliata* | wild | M | Mating success | 2 | 2 | (Corewyn 2015) |
| *Macaca thibetana* | provisioned | M | Mating success | 1 | 1 | (Xia et al., 2015) |
| *Pan paniscus* | wild | M | Mating success | 1 | 1 | (Surbeck et al. 2011) |
| *Eulemur fulvus mayottensis* | captive | Mixed | Feeding success | 1 | 1 | (Roeder and Fornasieri 1995) ^b^ |
| *Cercocebus torquatus atys* | wild | F | Feeding success | 1 | 1 | (Range and Noë 2002) |
| *Papio anubis* | wild | F | Feeding success | 1 | 1 | (Barton and Whiten 1993) ^b^ |
| *Colobus polykomos* | wild | F | Fecundity | 1 | 1 | (Korstjens et al. 2002) |
| *Erythrocebus patas* | wild | M | Mating success | 1 | 1 | (Chism and Rogers 1997) |
| *Macaca nigra* | wild | M | Mating success | 1 | 1 | (Reed et al. 1997) |
| *Cercopithecus mitis* | wild | F | Feeding success | 1 | 1 | (Payne et al. 2003) |
| *Semnopithecus entellus* | provisioned | F | Infant survival | 1 | 9 ^c^ | (Borries et al. 1991) |
| *Semnopithecus entellus* | provisioned | F | Fecundity | 1 |  |  |
| *Callithrix jacchus* | wild | M | Mating success | 2 | 2 | (Digby 1995a; Digby 1999) |
| *Callithrix jacchus* | wild | F | Fecundity | 1 | 1 | (Digby 1995a, 1995b) |
| *Callithrix jacchus* | wild | F | Infant survival | 1 |  |  |
| *Callithrix jacchus* | wild | F | Fecundity | 1 | 1 |  |
| *Callithrix jacchus* | wild | F | Infant survival | 1 |  |  |
| *Callithrix jacchus* | wild | F | Fecundity | 1 | 1 |  |
| *Callithrix jacchus* | wild | F | Infant survival | 1 |  |  |
| *Chlorocebus pygerythrus* | wild | F | Feeding success | 1 | 1 | (Isbell and Pruetz 1998; Isbell et al. 1999) |
| *Erythrocebus patas* | wild | F | Feeding success | 1 | 1 |  |
| *Pan troglodytes schweinfurthii* | wild | M | Mating success | 1 | 1 | (Newton-Fisher 2004) |
| *Piliocolobus tephrosceles* | wild | M | Mating success | 1 | 1 | (Struhsaker and Leland 1985) |
| *Papio anubis* | wild | M | Mating success | 1 | 1 | (Harding 1980) |
| *Cebus olivaceus* | wild | F | Feeding success | 1 | 1 | (Robinson 1981) |
| *Papio anubis* | wild | M | Mating success | 1 | 2 ^c^ | (Sapolsky 1983) |
| *Papio anubis* | wild | M | Mating success | 1 | 1 | (Strum 1982) |
| *Macaca sylvanus* | wild | M | Mating success | 1 | 1 | (Taub 1980; Taub 1982) |
| *Chlorocebus pygerythrus* | wild | F | Feeding success | 2 | 1 | (Whitten 1983) |
| *Chlorocebus pygerythrus* | wild | F | Fecundity | 1 |  |  |
| *Chlorocebus pygerythrus* | wild | F | Mating success | 1 |  |  |
| *Chlorocebus pygerythrus* | wild | F | Infant survival | 1 |  |  |
| *Chlorocebus pygerythrus* | wild | F | Feeding success | 1 | 1 |  |
| *Chlorocebus pygerythrus* | wild | F | Fecundity | 1 |  |  |
| *Chlorocebus pygerythrus* | wild | F | Mating success | 1 |  |  |
| *Chlorocebus pygerythrus* | wild | F | Infant survival | 1 |  |  |
| *Cercopithecus mitis* | wild | F | Infant survival | 1 | 1 | (Cords 2000) |
| *Pan troglodytes schweinfurthii* | wild | M | Mating success | 1 | 2 ^c^ | (Nishida and Hosaka 1996) |
| *Papio anubis* | wild | M | Mating success | 2 | 2 | (Smuts 1985) |
| *Pan troglodytes verus* | wild | M | Mating success | 1 | 1 | (Boesch and Boesch-Achermann 2000) |
| *Cercocebus galeritus* | wild | M | Mating success | 1 | 1 | (Mwamende 2009) |
| *Cercocebus galeritus* | wild | F | Mating success | 1 | 1 |  |
| *Papio cynocephalus* | wild | F | Mating success | 1 | 1 | (Collins 1981) |
| *Papio cynocephalus* | wild | M | Mating success | 2 | 2 |  |
| *Callithrix jacchus* | provisioned | Mixed | Feeding success | 3 | 3 | (De la Fuente et al. 2019) |
| *Pan troglodytes schweinfurthii* | provisioned | M | Feeding success | 1 | 1 | (Houle and Wrangham 2021) |
| *Pan troglodytes schweinfurthii* | provisioned | F | Feeding success | 1 | 1 |  |
| *Rhinopithecus roxellana* | provisioned | M | Feeding success | 1 | 1 | (Guo et al., 2020) |
| *Macaca thibetana* | provisioned | M | Mating success | 1 | 1 | (Li et al. 2015) |
| *Macaca thibetana* | provisioned | F | Mating success | 1 | 1 |  |
| *Rhinopithecus bieti* | provisioned | M | Feeding success | 1 | 1 | (Xia et al., 2017) |
| *Macaca thibetana* | provisioned | M | Mating success | 1 | 1 | (Xia, 2013) |
| *Pan paniscus* | captive | Mixed | Feeding success | 1 | 1 | (Vervaecke et al. 1999) |
| *Pan paniscus* | captive | Mixed | Feeding success | 1 | 1 | (Vervaecke et al. 2000) |
| *Gorilla gorilla gorilla* | captive | F | Feeding success | 1 | 2 ^c^ | (Scott and Lockard 1999, 2006) |
| *Alouatta palliata* | wild | F | Mating success | 1 | 1 | (Jones 1980; Hilpert and Jones 2005) |
| *Macaca leonina* | wild | M | Fecundity | 1 | 1 | (Trébouet 2019) |
| *Macaca leonina* | wild | M | Mating success | 1 | 1 |  |
| *Macaca assamensis* | wild | M | Mating success | 1 | 1 | (Ostner et al. 2011; Sukmak et al. 2014) ^d^ |
| *Macaca assamensis* | wild | M | Fecundity | 1 |  |  |
| *Macaca assamensis* | wild | M | Mating success | 1 | 1 |  |
| *Macaca assamensis* | wild | M | Fecundity | 1 |  |  |
| *Macaca assamensis* | wild | M | Fecundity | 2 | 2 |  |
| *Macaca assamensis* | wild | F | Feeding success | 2 | 2 | (Heesen et al. 2013) ^b, d^ |

^a^ The study presenting information for *NDS_Dij_*-based steepness only instead of the original matrix of aggressive interactions.

^b^ Studies presenting more than one measure of individual feeding success.

^c^ Studies presenting more than one matrix of aggressive interactions for calculating hierarchy steepness.

^d^ The matrices of aggressive interactions for calculating hierarchy steepness are provided by the authors (Schülke and Ostner) of the publication.

**Appendix 2** The unpublished data included in our dataset and the corresponding measures of fitness-related benefits used for the meta-regression analysis.

| **Species** | **Study setting** | **Sex category** | **Fitness-related benefit** | **Number of effect sizes obtained** | **Number of matrices available** | **Principal investigator (s)** |
| --- | --- | --- | --- | --- | --- | --- |
| *Macaca mulatta* | provisioned | M | Mating success | 4 | 4 | Kaburu, McCowan, Beisner, Bliss-Moreau, Balasubramaniam, Marty |
| *Macaca mulatta* | provisioned | M | Feeding success | 4 |  |  |
| *Macaca mulatta* | provisioned | F | Mating success | 4 | 4 |  |
| *Macaca mulatta* | provisioned | F | Feeding success | 4 |  |  |
| *Macaca radiata* | provisioned | M | Mating success | 2 | 2 | Balasubramaniam, Arlet, Marty, McCowan, Beisner, Bliss-Moreau, Kaburu |
| *Macaca radiata* | provisioned | M | Feeding success | 2 |  |  |
| *Macaca radiata* | provisioned | F | Mating success | 2 | 2 |  |
| *Macaca radiata* | provisioned | F | Feeding success | 2 |  |  |
| *Macaca fascicularis* | provisioned | M | Mating success | 4 | 4 | Marty, McCowan, Beisner, Bliss-Moreau, Balasubramaniam, Kaburu |
| *Macaca fascicularis* | provisioned | M | Feeding success | 4 |  |  |
| *Macaca fascicularis* | provisioned | F | Mating success | 4 | 4 |  |
| *Macaca fascicularis* | provisioned | F | Feeding success | 4 |  |  |
| *Macaca sylvanus* | provisioned | M | Mating success | 2 | 2 | Konečná |
| *Macaca sylvanus* | provisioned | F | Mating success | 2 | 2 |  |
| *Macaca sylvanus* | provisioned | M | Mating success | 1 | 1 | García-Nisa and Kendal |
| *Macaca sylvanus* | provisioned | M | Feeding success | 1 |  |  |
| *Macaca sylvanus* | provisioned | F | Mating success | 1 | 1 |  |
| *Macaca sylvanus* | provisioned | F | Feeding success | 1 |  |  |
| *Macaca sylvanus* | provisioned | M | Feeding success | 1 | 1 |  |
| *Macaca sylvanus* | provisioned | F | Feeding success | 1 | 1 |  |
| *Macaca sylvanus* | provisioned | Mixed | Feeding success | 2 | 2 |  |
| *Macaca nigra* | wild | M | Mating success | 2 | 2 | Micheletta and Duboscq |
| *Macaca nigra* | wild | F | Mating success | 1 | 1 |  |
| *Macaca nigra* | wild | F | Fecundity | 1 |  |  |
| *Macaca nigra* | wild | F | Mating success | 1 | 1 |  |
| *Macaca nigra* | wild | F | Fecundity | 1 |  |  |
| *Macaca nigra* | wild | M | Fecundity | 4 | 4 |  |
| *Macaca nigra* | wild | F | Fecundity | 3 | 3 |  |
| *Macaca mulatta* | provisioned | M | Fecundity | 6 | 6 | Brent |
| *Macaca mulatta* | provisioned | F | Fecundity | 7 | 7 |  |

**Supplementary material references**

Barton RA, Whiten A. 1993. Feeding competition among female olive baboons, *Papio anubis*. Anim Behav. 46(4):777–789. doi:10.1006/anbe.1993.1255.

Berghänel A, Schülke O, Ostner J. 2010. Coalition formation among Barbary macaque males: the influence of scramble competition. Anim Behav. 80(4):675–682. doi:10.1016/j.anbehav.2010.07.002.

Boesch C, Boesch-Achermann H. 2000. The Chimpanzees of the Taï-Forest: Behavioural Ecology and Evolution. 1st ed. Oxford University Press.

Borries C, Sommer V, Srivastava A. 1991. Dominance, age, and reproductive success in free-ranging female hanuman langurs (*Presbytis entellus*). Int J Primatol. 12(3):231–257. doi:10.1007/BF02547586.

Chism J, Rogers W. 1997. Male competition, mating success and female choice in a seasonally breeding primate (*Erythrocebus patas*). Ethology. 103(2):109–126. doi:10.1111/j.1439-0310.1997.tb00011.x.

Collins DA. 1981. Social behaviour and patterns of mating among adult yellow baboons (*Papio c. cynocephalus*. L. 1766) [Ph.D. thesis]. University of Edinburgh.

Cords M. 2000. Agonistic and affiliative relationships in a blue monkey group. In: Old World monkeys. Vol. 17. Cambridge, New York: Cambridge University Press. p. 453–479.

Corewyn LC. 2015. Dominance, access to females, and mating success among coresident male mantled howlers (*Alouatta palliata*) at La Pacifica, Costa Rica. Am J Primatol. 77(4):388–400. doi:10.1002/ajp.22355.

De la Fuente MF, Schiel N, Bicca-Marques JC, Caselli CB, Souto A, Garber PA. 2019. Balancing contest competition, scramble competition, and social tolerance at feeding sites in wild common marmosets (*Callithrix jacchus*). Am J Primatol. 81(4):e22964. doi:10.1002/ajp.22964.

Digby LJ. 1995a. Social organization in a wild population of *Callithrix jacchus*: II. Intragroup social behavior. Primates. 36(3):361–375. doi:10.1007/BF02382859.

Digby LJ. 1995b. Infant care, infanticide, and female reproductive strategies in polygynous groups of common marmosets (*Callithrix jacchus*). Behav Ecol Sociobiol. 37(1):51–61. doi:10.1007/BF00173899.

Digby LJ. 1999. Sexual behavior and extragroup copulations in a wild population of common marmosets (*Callithrix jacchus*). Folia Primatol (Basel). 70(3):136–145. doi:10.1159/000021686.

Guo S, He S, Zhang H, Bai R, Zhang S, Hou R, Grueter CC, Chapman CA, Dunn DW, Li B. 2020. Male social rank and food competition in a primate multi-level society. Am J Phys Anthropol. 173(4):630–642. doi:10.1002/ajpa.24141.

Harding RSO. 1980. Agonism, ranking, and the social behavior of adult male baboons. Am J Phys Anthropol. 53(2):203–216. doi:10.1002/ajpa.1330530205.

He H, Zhao H, Qi X, Wang X, Guo S, Ji W, Wang C, Wei W, Li B. 2013. Dominance rank of adult females and mating competition in Sichuan snub-nosed monkeys (*Rhinopithecus roxellana*) in the Qinling Mountains, China. Chin Sci Bull. 58(18):2205–2211. doi:10.1007/s11434-012-5649-2.

Heesen M, Rogahn S, Ostner J, Schülke O. 2013. Food abundance affects energy intake and reproduction in frugivorous female Assamese macaques. Behav Ecol Sociobiol. 67(7):1053–1066. doi:10.1007/s00265-013-1530-9.

Hilpert AL, Jones CB. 2005. Possible costs of radio-tracking a young adult female mantled howler monkey (*Alouatta palliata*) in deciduous habitat of Costa Rican tropical dry forest. J Appl Anim Welf Sci. 8(3):227–232. doi:10.1207/s15327604jaws0803_6.

Houle A, Wrangham RW. 2021. Contest competition for fruit and space among wild chimpanzees in relation to the vertical stratification of metabolizable energy. Anim Behav. 175:231–246. doi:10.1016/j.anbehav.2021.03.003.

Isbell LA, Pruetz JD. 1998. Differences between vervets (*Cercopithecus aethiops*) and patas monkeys (*Erythrocebus patas*) in agonistic interactions between adult females. Int J Primatol. 19(5):837–855. doi:10.1023/A:1020393329574.

Isbell LA, Pruetz JD, Lewis M, Young TP. 1999. Rank differences in ecological behavior: A comparative study of patas monkeys (*Erythrocebus patas*) and vervets (*Cercopithecus aethiops*). Int J Primatol. 20(2):257–272. doi:10.1023/A:1020574504017.

Jones CB. 1980. The functions of status in the mantled howler monkey, *Alouatta palliata* GRAY: Intraspecific competition for group membership in a folivorous neotropical primate. Primates. 21(3):389–405. doi:10.1007/BF02390468.

Korstjens AH, Sterck EHM, Noë R. 2002. How adaptive or phylogenetically inert is primate social behaviour? A test with two sympatric colobines. Behaviour. 139(2–3):203–225. doi:10.1163/156853902760102654.

Li Z, Li J, Xia D, Zhu Y, Wang X, Zhang D. 2015. Mating strategies of subordinate males in Tibetan macaques (*Macaca thibetana*) at Mt. Huangshan, China (in Chinese). Acta Theriol Sin. 35(1):29–39.

Mwamende KA. 2009. Social organisation, ecology and reproduction in the Sanje mangabey (*Cercocebus sanjei*) in the Udzungwa Mountains National Park, Tanzania [Master’s thesis]. Victoria University of Wellington.

Newton-Fisher NE. 2004. Hierarchy and social status in Budongo chimpanzees. Primates. 45(2):81–87. doi:10.1007/s10329-003-0064-6.

Nishida T, Hosaka K. 1996. Coalition strategies among adult male chimpanzees of the Mahale Mountains, Tanzania. In: McGrew WC, Marchant LF, Nishida T, editors. Great Ape Societies. Cambridge: Cambridge University Press. p. 114–134.

Ostner J, Heistermann M, Schülke O. 2011. Male competition and its hormonal correlates in Assamese macaques (*Macaca assamensis*). Horm Behav. 59(1):105–113. doi:10.1016/j.yhbeh.2010.10.017.

Payne HFP, Lawes MJ, Henzi SP. 2003. Competition and the exchange of grooming among female samango monkeys (*Cercopithecus mitis erythrarchus*). Behaviour. 140(4):453–471. doi:10.1163/156853903322127931.

Range F, Noë R. 2002. Familiarity and dominance relations among female sooty mangabeys in the Taï National Park. Am J Primatol. 56(3):137–153. doi:10.1002/ajp.1070.

Reed C, O’Brien TG, Kinnaird MF. 1997. Male social behavior and dominance hierarchy in the Sulawesi crested black Macaque (*Macaca nigra*). Int J Primatol. 18(2):247–260. doi:10.1023/A:1026376720249.

Robinson JG. 1981. Spatial structure in foraging groups of wedge-capped capuchin monkeys *Cebus nigrivittatus*. Anim Behav. 29(4):1036–1056. doi:10.1016/S0003-3472(81)80057-7.

Roeder J-J, Fornasieri I. 1995. Does agonistic dominance imply feeding priority in lemurs? a study in *Eulemur fulvus mayottensis*. Int J Primatol. 16(4):629–642. doi:10.1007/BF02735285.

Sapolsky RM. 1983. Endocrine aspects of social instability in the olive baboon (*Papio anubis*). Am J Primatol. 5(4):365–379. doi:10.1002/ajp.1350050406.

Scott J, Lockard JS. 1999. Female dominance relationships among captive western lowland gorillas: Comparisons with the wild. Behaviour. 136(10–11):1283–1310. doi:10.1163/156853999500721.

Scott J, Lockard JS. 2006. Captive female gorilla agonistic relationships with clumped defendable food resources. Primates. 47(3):199–209. doi:10.1007/s10329-005-0167-3.

Smuts BB. 1985. Chapter 7 Male-male competition for mates. In: Smuts BB, editor. Sex and friendship in baboons. New York: Aldine Publishing Company. p. 122–157.

Struhsaker TT, Leland L. 1985. Infanticide in a Patrilineal Society of Red Colobus Monkeys. Z Für Tierpsychol. 69(2):89–132. doi:10.1111/j.1439-0310.1985.tb00139.x.

Strum SC. 1982. Agonistic dominance in male baboons: an alternative view. Int J Primatol. 3(2):175–202. doi:10.1007/BF02693494.

Sukmak M, Wajjwalku W, Ostner J, Schülke O. 2014. Dominance rank, female reproductive synchrony, and male reproductive skew in wild Assamese macaques. Behav Ecol Sociobiol. 68(7):1097–1108. doi:10.1007/s00265-014-1721-z.

Surbeck M, Mundry R, Hohmann G. 2011. Mothers matter! Maternal support, dominance status and mating success in male bonobos (*Pan paniscus*). Proc R Soc B Biol Sci. 278(1705):590–598. doi:10.1098/rspb.2010.1572.

Taub DM. 1980. Testing the ‘agonistic buffering’ hypothesis - I. The dynamics of participation in the triadic interaction. Behav Ecol Sociobiol. 6(3):187–197. doi:10.1007/BF00569200.

Taub DM. 1982. Sexual behavior of wild Barbary macaque males (*Macaca sylvanus*). Am J Primatol. 2(1):109–113. doi:10.1002/ajp.1350020112.

Trébouet F. 2019. Male Reproductive Strategies in Wild Northern Pig-Tailed Macaques (*Macaca leonina*): Testing the Priority-of-Access Model [Ph.D. thesis]. [Carbondale, US]: Southern Illinois University.

Vervaecke H, de Vries H, van Elsacker L. 1999. An experimental evaluation of the consistency of competitive ability and agonistic dominance in different social contexts in captive bonobos. Behaviour. 136(4):423–442. doi:10.1163/156853999501405.

Vervaecke H, de Vries H, van Elsacker L. 2000. Dominance and its behavioral measures in a captive group of bonobos (*Pan paniscus*). Int J Primatol. 21(1):47–68. doi:10.1023/A:1005471512788.

Whitten PL. 1983. Diet and dominance among female vervet monkeys (*Cercopithecus aethiops*). Am J Primatol. 5(2):139–159. doi:10.1002/ajp.1350050205.

Xia D. 2013. Exchange/interchange of grooming and group stability in Tibetan Macaques (*Macaca thibetana*) (in Chinese) [Ph.D. thesis]. [Anhui, China]: Anhui University.

Xia D, Li J, Sun B, Weed JL, Kyes RC. 2015. Evaluation of fecal testosterone, rank and copulatory behavior in wild male *Macaca thibetana* at Huangshan, China. Pak J Zool. 47(5):1445–1454.

Xia W, Hu J, Ren B, He X, Kuang P, Zhong T, Li D. 2017. Dominance hierarchy for one-male units in a group of provisioned black-and-white snub-nosed monkeys (*Rhinopithecus bieti*) (in Chinese). Acta Theriol Sin. 37(4):371–378. doi:10.16829/j.slxb.201704007.
