# Supplementary material for: Relationship between dominance hierarchy steepness and rank-relatedness of benefits in primates
Source: Behav Ecol. 2024 Aug 13;35(5):arae066. doi: 10.1093/beheco/arae066 (PMC11347755; doi:10.1093/beheco/arae066)
Supplement: arae066_suppl_Supplementary_Material_S2 [file arae066_suppl_supplementary_material_s2.docx]

**Online questionnaire sent to researchers to collect unpublished data**

1. **What is your name?**

1. **What is your email address (so I can contact you with updates on the study)?**

1. **What is the scientific name of your study species (please fill in the survey as many times as the number of species you have data on)?**

1. **What is the location of your study group?**

1. **What are the conditions of your study group?**

Captive animals

Free-ranging and provisioned

Free-ranging

1. **What is the approximate duration of the study for which you have data?**

|  | Please enter figures here |
| --- | --- |
| Length of data collection (in months) |  |
| Total observation time (in hours) |  |

1. **Do you have matrices of aggression and/or displacement for your study animals?**

Yes

No

1. **If you answered 'yes' above, on which sex do you have those matrices?**

Females only

Males only

Females and males together

Females and males in separate matrices

1. **Do you have data on any fitness measure?**

Yes

No

1. **If you have data on any fitness measure, please specify what measure(s) you have data on (e.g. feeding success, mating success, infant survival or fecundity)? You can list here all the measures you have data on**

1. **On which sex do you have those data on fitness measures?**

Females only

Males only

Females and males together

Females and males separately

1. **Are your data in matrices and on fitness measures on the same study animals, and collected during the same study period?**

Yes

No

1. **If you are interested in collaborating on this project, how quickly could you send us your data? Please add a date (in the format day:month:year)**

1. **Do you have any additional comment (e.g. details on fitness measure) or question?**
